# The impact of HIV and tuberculosis interventions on South African adult tuberculosis trends, 1990-2019: a mathematical modeling analysis

**DOI:** 10.1016/j.ijid.2022.07.047

**Published:** 2022-09

**Authors:** Mmamapudi Kubjane, Muhammad Osman, Andrew Boulle, Leigh F. Johnson

**Affiliations:** 1 Centre for Infectious Disease Epidemiology and Research, School of Public Health and Family Medicine, University of Cape Town, Cape Town, South Africa; 2 Desmond Tutu TB Centre, Department of Paediatrics and Child Health, Faculty of Health Sciences, Stellenbosch University, Cape Town, South Africa; 3 School of Human Sciences, Faculty of Education, Health and Human Sciences, University of Greenwich, London, United Kingdom; 4 Western Cape Provincial Department of Health, Cape Town, South Africa

**Keywords:** Mathematical modeling, Tuberculosis, Human immunodeficiency virus, Tuberculosis programmatic interventions, South Africa

## Abstract

**Objectives:**

To quantify the South African adult tuberculosis (TB) incidence and mortality attributable to HIV between 1990 and 2019 and to estimate the reduction in TB incidence due to directly observed therapy, antiretroviral therapy (ART), isoniazid preventive therapy, increased TB screening, and Xpert MTB/RIF.

**Methods:**

We developed a dynamic TB transmission model for South Africa. A Bayesian approach was used to calibrate the model to South African-specific data sources. Counterfactual scenarios were simulated to estimate TB incidence and mortality attributable to HIV and the impact of interventions on TB incidence.

**Results:**

Between 1990 and 2019, 8.8 million (95% confidence interval [CI] 8.3-9.3 million) individuals developed TB, and 2.1 million (95% CI 2.0-2.2 million) died from TB. A total of 55% and 69% of TB cases and mortality were due to HIV, respectively. Overall, TB screening and ART substantially reduced TB incidence by 28.2% (95% CI 26.4-29.8%) and 20.0% (95% CI 19.2-20.7%), respectively, in 2019; other interventions had minor impacts.

**Conclusion:**

HIV has dramatically increased TB incidence and mortality in South Africa. The provision of ART and intensification of TB screening explained most recent declines in TB incidence.

## Highlights

• We developed a tuberculosis (TB) transmission dynamic model for South Africa.
• We quantified TB incidence and mortality attributable to HIV between 1990 and 2019.
• We estimated the impact of TB programmatic interventions on TB incidence between 1996 and 2019.
• A significant proportion of TB incidence and mortality was attributable to HIV.
• TB screening and antiretroviral therapy led to substantial TB incidence reductions.

## Introduction

South Africa is ranked among the top 20 high tuberculosis (TB) burden countries (World Health Organization, 2019). The TB epidemic grew rapidly in the early 1990s, primarily driven by HIV (Abdool Karim *et al.*, 2009). HIV infection is the strongest individual-level TB risk factor, increasing the risk of progression to TB disease and reactivation of latent TB infection, worsening treatment outcomes and increasing mortality (Lawn *et al.*, 2009). Although the effect of HIV on TB has been established, very few studies have quantified its population-level effect on incidence and mortality over time. South Africa has implemented TB control interventions, including directly observed therapy (DOTS), which was scaled up in 1996 (World Health Organization, 1994). This strategy had multiple components, including directly observed treatment, political commitment, improved microscopy services, surveillance and monitoring, and quality treatment (World Health Organization, 1994). Other interventions, which were scaled up in the mid-2000s, included the provision of isoniazid preventive therapy (IPT) and antiretroviral therapy (ART) for HIV-positive individuals (Churchyard *et al.*, 2014b; Lawn *et al.*, 2011). Declines in TB notifications and mortality from 2008 have largely been attributed to ART, which was made widely available during the mid-2000s (Dye and Williams, 2019; Loveday *et al.*, 2019; Surie *et al.*, 2018). This is supported by the established individual-level effectiveness of ART in reducing TB incidence and mortality (Lawn *et al.*, 2006; Ross *et al.*, 2021). In addition, IPT also reduces the risk of developing TB in people living with HIV (Ayele *et al.*, 2015; Ross *et al.*, 2021). However, few studies have shown the population-level effect of IPT.

Substantial effort has also been invested in identifying TB cases in South Africa. Between 2004 and 2012, the annual number of microbiological TB tests performed doubled (Nanoo *et al.*, 2015). Xpert MTB/RIF was introduced in 2011 to replace smear microscopy (Di Tanna *et al.*, 2019; Theron *et al.*, 2014b). Although earlier modeling studies anticipated substantial health benefits from Xpert MTB/RIF implementation compared with microscopy (Menzies *et al.*, 2012), clinical trials have found minimal or no impact on TB mortality (Di Tanna *et al.*, 2019). To understand these dynamics better, modeling studies with detailed diagnostic algorithms that account for empirical treatment are required.

There have been no formal analyses to quantify the contribution of the abovementioned TB interventions to the declining TB trends. Such evaluations are essential in assessing which South African TB program components are most critical to decreasing TB incidence. Therefore, we sought to (i) describe the South African TB epidemic trends between 1990 and 2019; (ii) assess the burden of TB attributable to HIV; and (iii) assess the impact of TB interventions, including DOTS, increased TB screening, Xpert MTB/RIF as an additional first-line diagnostic tool replacing smear microscopy, IPT, and ART on TB incidence.

## Methods

We developed an age- and sex-structured deterministic compartmental model of TB and HIV for the South African adult population (aged 15 years and older). The core TB states are modeled following conventions described by previous studies (Menzies *et al.*, 2012). Transitions between states include TB infection, progression to TB disease, natural recovery, diagnosis and treatment initiation, death, and treatment cure. Age- and sex-specific relative risks were applied to rates of progression to TB disease to capture age and sex differences in TB risk factors.

**Figure 1:**
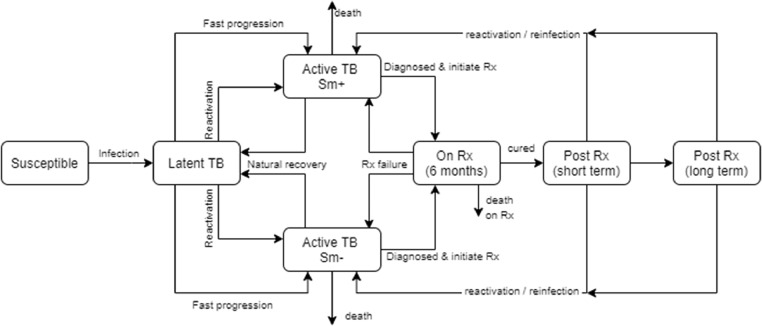
The TB natural history model structure Rx = treatment. Sm+ = smear-positive. Sm- = smear-negative.

After cure by TB treatment, two post-treatment states dependent on time since cure are defined: short term (within 6 months after cure) and long term (6 or more months after cure). In both states, individuals are at risk of reinfection, whereas in the short term, individuals are assumed to have a high likelihood of relapse (den Boon *et al.*, 2007). The natural history parameters are described in Table 1. The model structure is shown in Figure 1. A detailed description of the model is provided in the supplementary material. The risk of infection depends on the mean contact rates, age- and sex-mixing patterns (Dodd *et al.*, 2016), the probability of transmission per contact, and the prevalence of infectious TB.

**Table 1:** Key model parameters.

Parameter description	Mean	Standard deviation	Varied / fixed	Section described in supplementary
The proportion of incident TB cases in HIV-negative adults that are smear-positive	0.51		Fixed	3
TB transmission probability per contact per day (if an infectious individual is smear-positive)	0.0025	0.0025	Varied	4
Relative rate of infectivity smear-negative compared to smear-positive	0.206		Fixed	4
The annual rate of reactivation in HIV-negative individuals	0.00148		Fixed	5
The proportion of individuals experiencing fast progression	0.112		Fixed	5
Reduction in TB incidence in previously infected individuals if HIV-negative	0.79		Fixed	5
Relative rate of immunity to TB per 100-cell increase in CD4	1.1		Fixed	5
Relative rate of TB incidence per 100-cell increase in CD4	0.703		Fixed	5
Annual natural recovery rate in smear-positive TB, HIV-negative individuals	0.075		Fixed	5
Annual natural recovery rate in smear-negative TB, HIV-negative individuals	0.224		Fixed	5
Smear-negative TB mortality (untreated)	0.049		Fixed	5
Smear-positive TB mortality (untreated)	0.196		Fixed	5
Relative rate of TB incidence on ART (controlling for CD4)	0.81	0.05	Varied	5
Prevalence of cough >2 weeks duration in individuals with smear-negative TB	0.198		Fixed	6
Ratio of symptoms in patients with smear-positive compared to smear-negative TB	3.03		Fixed	6
The annual rate of health-seeking in males with smear-negative TB	2.14	0.49	Varied	6
The annual rate of health-seeking in males in the general population	1.15	0.5	Varied	6
The annual rate of health-seeking in males due to TB-like symptoms	0.22	0.15	Varied	6
The proportion of active TB cases seeking treatment who are treated empirically if no microbiological test is done	0.125	0.144	Varied	6
The proportion of smear-negative TB cases who are treated empirically if they initially screened negative on smear test	0.333	0.236	Varied	6
Relative rate of empirical treatment if not seeking treatment because of TB symptoms	0.5	0.289	Varied	6
Relative rate empirical treatment if symptoms are not due to TB	0.5	0.289	Varied	6
Reduction in empiric treatment after a negative screen due to Xpert MTB/RIF	0.5	0.18	Varied	6
Relative rate of health-seeking in women, compared to men	1.55	0.17	Varied	6
Relative rate of health-seeking in HIV-positive compared to HIV-negative individuals	3	1	Varied	6
Relative rate of screening in TB patients seeking treatment for TB symptoms, compared to those seeking treatment for other conditions: initial^(a)^	8.71	2.5	Varied	6
Relative rate of screening in TB patients seeking treatment for TB symptoms, compared to those seeking treatment for other conditions: ultimate^(a)^	4	1.2	Varied	6
Probability of cure if a patient dropped out before completing TB treatment	0.65		Fixed	7
Increase in TB mortality rate per 10-year increase in age	1.4		Fixed	7
The annual mortality rate in HIV-negative individuals receiving TB treatment^(b)^	0.192		Fixed	7
The relative rate of TB mortality per 50-cells increase in CD4 count if HIV+	0.95		Fixed	7
Relative rate of TB mortality if on ART	0.55	0.08	Varied	7
Increase in TB risk if previously experienced TB	3.03		Fixed	8
Rate of relapse in short-term post-treatment state	0.1		Fixed	8
Increase in TB incidence due to alcohol abuse	1.94^(c)^		Fixed	10
Increase in TB incidence due to diabetes (HbA1c > 6.5%)	2.59^(c)^		Fixed	10
Increase in TB risk if currently smoking	0.47^(c)^		Fixed	10
Increase in TB risk per 10-year increase in the duration of smoking	0.38^(c)^		Fixed	10
Increase in TB risk due to low BMI	0.8^(c)^		Fixed	10

ART=antiretroviral therapy;

BMI=body mass index; HbA1c= Glycated hemoglobin. TB=tuberculosis.

All rates are annual rates unless specified otherwise. The supplementary material in the indicated sections provides further descriptions and references for the model parameters. (a): This is a time-varying parameter. The initial rate applies up to 2005, the ultimate rate applies from 2012, with linear interpolation over the intervening years (2006-2011). (b): Applies when most people get treated in the very advanced stages of disease (i.e., when screening rates are close to zero). (c): A value of 1.94, for example, is equivalent to a relative risk of 2.94, comparing individuals with the exposure to individuals in the baseline category (supplementary material).

The Thembisa HIV model forms the HIV component of the model (Johnson and Dorrington, 2020). Thembisa is a compartmental model of the South African HIV epidemic, designed to answer policy questions relating to HIV prevention and treatment. The model is age- and sex-structured, and the HIV epidemic is simulated dynamically from 1985. Subpopulations who are HIV-positive are further stratified by HIV testing history, CD4 count, and duration since ART initiation. The model also captures changes in the ART guidelines over time (Johnson and Dorrington, 2020) and is calibrated to South African data. HIV is assumed to affect the TB natural history parameters. These HIV effects are modeled as relative risks, which vary by CD4 count and duration since ART initiation (Table 1).

IPT is modeled for individuals with latent tuberculosis infection who are HIV-positive and eligible as per guidelines (Department of Health, 2013). Uptake depends on CD4 count, ART duration, and latent TB status. IPT uptake started in 2010; the number of IPT initiators was obtained from the District Health Information System. Assumptions on IPT duration, completion, and efficacy are in the supplementary material.

The assumed health-seeking patterns in the model are based on South African studies. We assumed different health facility attendance rates for individuals: (i) with TB, attending health facilities due to TB-related symptoms; (ii) without TB, attending due to other health conditions; and (iii) without TB, attending due to TB-like symptoms. We assumed females were more likely to seek care than males (Horton *et al.*, 2016), and individuals who are HIV-positive had higher health-seeking rates than individuals who are HIV-negative (Corbett *et al.*, 2003). Individuals who are smear-positive experience more TB symptoms than individuals who are smear-negative (Onozaki *et al.*, 2015). We consider smear microscopy and Xpert MTB/RIF as the first-line diagnostic tools, accounting for the phased implementation of Xpert MTB/RIF from 2011 onwards. To estimate the numbers of true- and false-positive TB diagnoses, we specified the sensitivity and specificity of these tests. After initial negative test results, we assumed a proportion of individuals were followed up for a second test by culture.

The model also allows treatment initiation in a proportion of individuals who do not have laboratory-confirmed TB (empirical treatment). Health-seeking parameters and rates of TB screening are estimated through calibration by fitting the model to the number of microbiological tests performed (Nanoo *et al.*, 2015) and number of cases treated. Once individuals start the 6-month TB treatment course, the following outcomes are considered: cure, failure, discontinuation, and death (with the rates of cure and failure depending on treatment discontinuation rates). Treatment outcome assumptions are based on the electronic TB treatment register (ETR.net) data for drug-susceptible TB and are shown in the supplementary material.

### Calibration targets and data sources

A Bayesian approach was used to calibrate the model. Prior distributions were set to represent uncertainty in key model parameters (Table 1). Four main data sources were used for the calibration targets. First are the sex-stratified recorded numbers of TB deaths from the vital register for 1997-2016. These mortality data were adjusted for misclassification and under-reporting (supplementary material). Second, we relied on ETR.net for the numbers of individuals initiating treatment (2004-2016), deaths on treatment (2004-2016), and HIV prevalence in individuals on treatment (2008-2016). Third, we relied on the National Institute for Communicable Diseases for the number of microbiological tests performed (2004-2012) and positive TB diagnoses (2004-2019) (Nanoo *et al.*, 2015). Lastly, we used the 2018 national TB prevalence survey to calibrate the prevalence of active TB disease (Department of Health, 2021). For the calibration process, likelihood functions were defined to represent the goodness of fit to each calibration target, allowing for possible under- or over-reporting in the vital register and the ETR.net data.

We simulated posterior distributions numerically using incremental mixture importance sampling, that is, using importance sampling to draw a sample of parameter combinations from regions of the parameter space that yield the highest likelihood values (Raftery and Bao, 2010). The means for the model estimates were calculated over 1000 posterior samples, and 95% confidence intervals (CIs) were calculated by taking the 2.5th and the 97.5th percentiles of the posterior sample (supplementary materials).

We performed a sensitivity analysis to assess how the model inputs that varied in the calibration process were correlated with the estimated TB incidence and mortality for 2019.

### Model experiments to assess the impact of HIV and programmatic interventions over time

To quantify the effects of HIV, DOTS, increased TB screening, Xpert MTB/RIF, ART, and IPT on TB incidence and mortality, we ran the scenarios A-H described in Table 2. Each of the counterfactual scenarios B-H was compared with baseline scenario A to assess the change in TB incidence and mortality attributable to the relevant factor.

**Table 2:** Model experiments to assess the impact of HIV and programmatic interventions over time

Scenario	Model scenario descriptions	Parameters used
A	The baseline scenario represents the interventions currently in place: • DOTS was introduced in 1996, • smear microscopy as the dominant diagnostic tool before 2011, with Xpert MTB/RIF gradually implemented from 2011, • public-sector ART scale-up from 2004, • implementation of IPT from 2010.	• We assumed the relative rate of treatment discontinuation was 0.48 under DOTS (Pasipanodya and Gumbo, 2013). • Xpert MTB/RIF is assumed to be more sensitive than microscopy, but is associated with reduced empirical treatment. • ART is assumed to reduce TB incidence and mortality, through both direct effects on viral load, and indirect effects on CD4 count (Table 1). • IPT is assumed to reduce TB incidence by 52% in latently-infected adults (Ayele *et al.*, 2015).
B	To assess the burden of TB attributable to HIV, we simulated a scenario with no HIV infection.	HIV transmission probabilities were set to zero, so that there was no HIV epidemic.
C	To assess the impact of DOTS, we simulated a scenario without DOTS.	Treatment discontinuation rates held constant (no reduction due to DOTS).
D	To assess the impact of IPT, we simulated a scenario where no IPT is implemented.	The number of HIV-infected individuals initiated on isoniazid preventive therapy in each year was set to zero.
E	To assess the impact of ART, we simulated a scenario where there is no ART.	Annual numbers of ART initiations are set to zero.
F	To assess the impact of scaling up TB screening, we simulated a scenario where testing rates after 2004 remain the same as the 2004 rates.	Screening rates calculated from numbers of microbiological TB tests performed in 2004 are assumed to apply in all subsequent years.
G	To assess the impact of the introduction of Xpert MTB/RIF, we simulated a scenario where Xpert MTB/RIF was not introduced.	Numbers of microbiological TB tests performed by year are unchanged, but all testing is assumed to be based on microscopy.
H	To assess what would have happened without any programmatic changes, we simulated a scenario without any interventions in C to G.	Including all changes described in C-G.

ART = antiretroviral therapy; DOTS = directly observed therapy; IPT = isoniazid preventive therapy.

## Results

**Figure 2:**
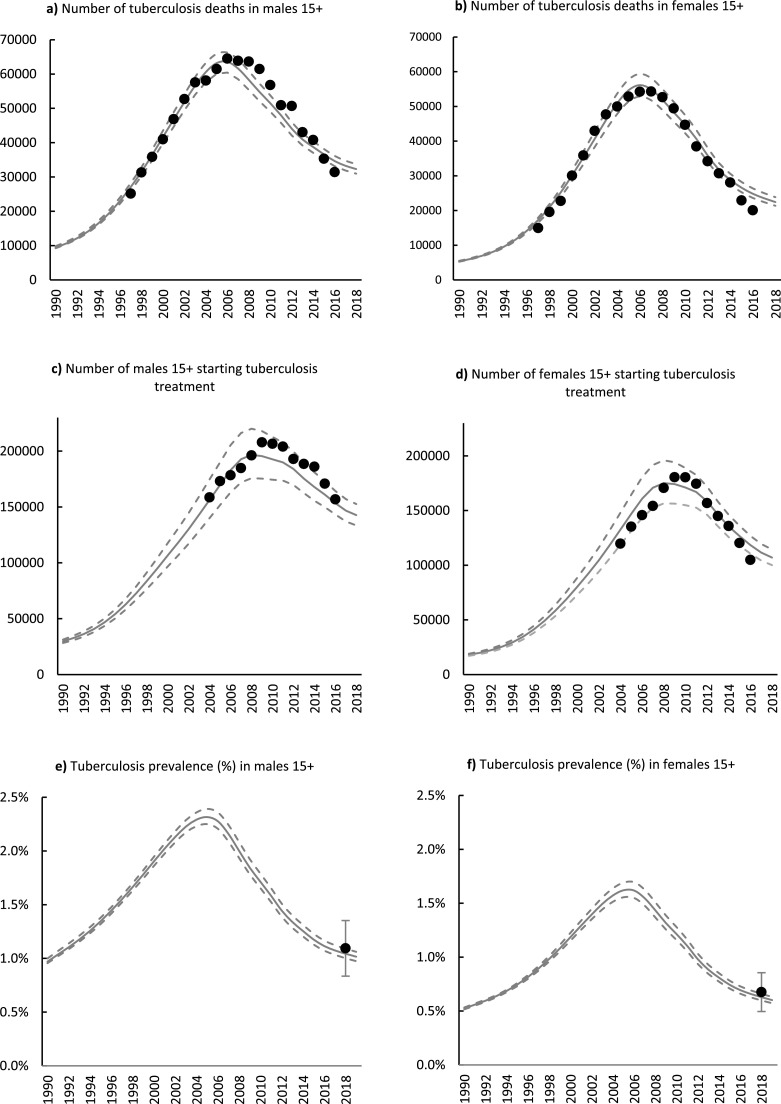
Estimated adult TB trends and calibration data by sex, 1990-2019. Gray solid lines represent model estimates, and dashed lines represent 95% confidence intervals. Black dots represent adjusted recorded mortality in 2a and 2b; people initiating treatment recorded on the electronic TB treatment register in 2c and 2d, and the national TB prevalence with 95% confidence intervals around point estimates in 2e and 2f.

The estimated number of TB deaths for males and females was consistent with the recorded number of deaths. The estimated deaths rapidly increased from 1994, peaked in 2006, followed by a decline to 31,000 (95% CI 30,000-33,000) and 21,000 (95% CI 21,000-22,000) in 2019, in males and females, respectively (Figures 2a and 2b). The model estimates for the numbers of people starting treatment were slightly inconsistent with the data. Before 2010, the model overestimated the number of females initiating treatment. After 2008, the model underestimated the number of males initiating treatment (Figures 2c and 2d). The estimated TB prevalence was reasonably close to the results of the 2018 TB prevalence survey; in 2019, the estimated TB prevalence was 1.02% (95% CI 0.97-1.06%) and 0.6% (95% CI 0.52-0.57%) in males and females, respectively (Figures 2e and 2f).

**Figure 3:**
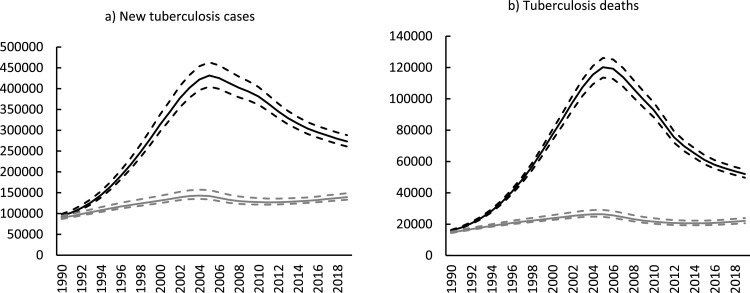
Impact of HIV on TB incidence and mortality, 1990-2019. The solid gray line represents the counterfactual scenario where there is no HIV assumed in the model. The solid black line represents the baseline scenario where HIV is present. The dashed lines represent 95% confidence intervals.

In the counterfactual scenario, without HIV, the model estimated that TB incidence and mortality would have remained relatively low (Figure 3), although still high enough for South Africa to be classified as a high TB burden country.

In the presence of HIV, the number of incident TB cases and deaths increased rapidly during the early 1990s and peaked in the mid-to-late 2000s, followed by declines until 2019 (Figure 3). The model estimated 273,000 (95% CI 261,000-288 000) new cases and 52,000 (95% CI 50,000-55,000) deaths in 2019. Over the 10-year period from 2009-2019, the percentage reduction in new TB cases and deaths was 30.4% (95% CI 29.4-31.5%) and 47.7% (95% CI 46.2-49.1%), respectively.

Cumulatively, between 1990 and 2019, there were 8,800,000 (95% CI 8,300,000-9,300,000) new TB cases and 2,100,000 (95% CI 2,000,000-2,200,000) TB deaths. Overall, 55.4% (95% CI 54.7-56.1%) of new TB cases and 68.5% (95% CI 67.0-69.7%) of TB deaths are attributable to HIV over the 1990-2019 period. A total of 57% of the new TB cases were in individuals who are HIV-positive, and 69% of TB deaths were in individuals who are HIV-positive.

**Figure 4:**
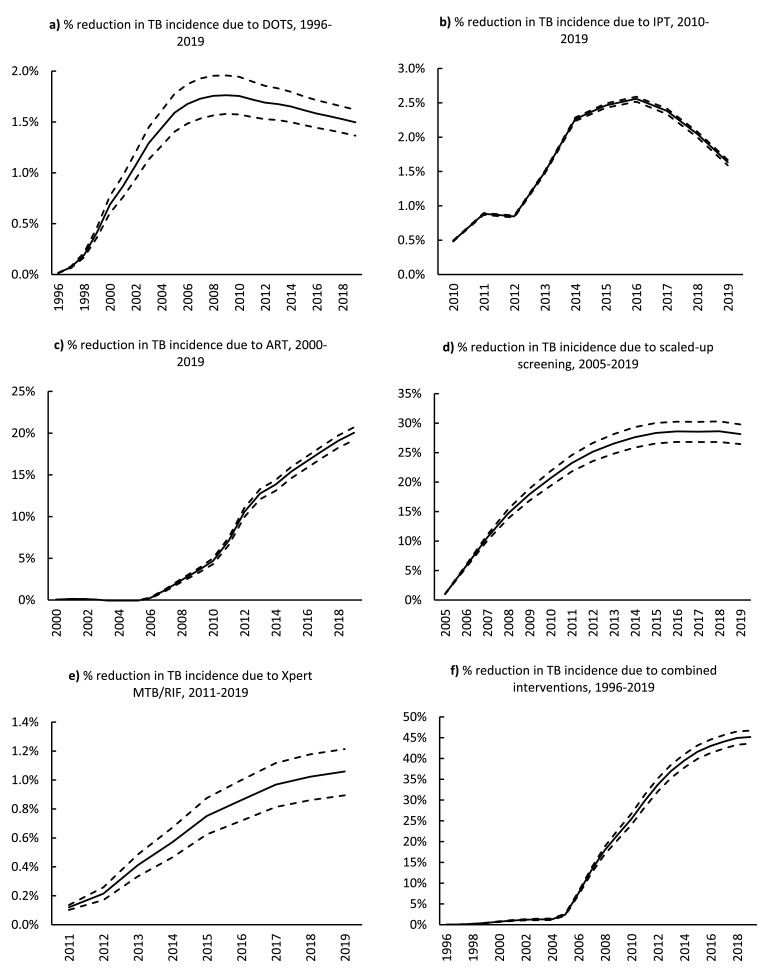
The impact of programmatic interventions on TB incidence: a) DOTS, b) IPT, c) ART, d) scaled-up TB screening, e) Xpert MTB/RIF, and f) all interventions combined. Solid lines represent the estimated mean reductions in TB incidence. All dashed lines represent the 95% confidence intervals. ART=antiretroviral therapy. DOTS=Directly Observed Therapy; IPT=isoniazid preventive therapy.

Reductions in TB incidence due to DOTS and IPT were small (<3%) in all years (Figures 4a and 4b). On the other hand, the reduction in TB incidence due to ART was evident from 2006. The impact of ART increased monotonically until 2019, with a reduction of 20.0% (95% CI 19.2-20.7%) in TB incidence (Figure 4c).

The reduction in TB incidence due to screening consistently increased from 2005-2019, reaching a maximum of 28.2% (95% CI 26.4-29.8%) (Figure 4d). On the other hand, the reduction in TB incidence due to Xpert MTB/RIF was very low (<1.3%) for all years (Figure 4e). However, between 2011 and 2019, Xpert MTB/RIF reduced the number of individuals without TB who initiated TB treatment by 56% (counterfactual: 52,000 vs baseline: 23,000). In addition, Xpert MTB/RIF also reduced the number of individuals starting treatment on an empirical basis by 28% (counterfactual: 65,000 vs baseline: 46,000).

All interventions combined (DOTS, IPT, ART, scaled-up screening, and Xpert MTB/RIF) contributed to a 45.2% (95% CI 43.6-46.7%) reduction in TB incidence in 2019 (Figure 4f).

Apart from DOTS and IPT, most interventions had a greater impact on TB mortality than on TB incidence (supplementary material, Figure 17). For example, reductions in mortality in 2019 due to interventions were 37.6% (95% CI 35.8-39.5%) for ART, 37.9% (95% CI 37.1-38.6%) for scaled-up TB screening, and 3.2% (95% CI 2.4-3.8%) for Xpert MTB/RIF. All interventions combined (DOTS, IPT, ART, scaled-up screening, and Xpert MTB/RIF) led to a 63.1% (95% CI 61.1-64.4%) reduction in TB mortality in 2019.

Most of the model input parameters had the expected relationships with the outcomes (shown by correlation coefficients and scatter plots). However, because several parameters were varied simultaneously in the calibration process, some counterintuitive associations need to be interpreted in terms of correlations between model parameters (for further discussion, see section 18 of the Supplementary materials).

## Discussion

HIV has had a devastating impact on TB incidence and mortality. Between 1990 and 2019, 8.8 million South Africans developed TB, and 2.1 million lives were lost. HIV caused 55% (4.8 million) of the TB cases and 69% (1.4 million) of TB deaths. We also showed that interventions implemented by the South African TB program have led to notable reductions in TB incidence, with ART and increased screening contributing to most of the decline. Our model also showed that the other interventions—DOTS, IPT, and Xpert MTB/RIF—had modest impacts on TB incidence. For most of the interventions (increased screening, ART, and Xpert MTB/RIF), the impact on TB mortality was proportionately greater than the impact on TB incidence (Supplementary material).

Although HIV is the strongest driver of the TB epidemic, our model estimated that even in the absence of HIV, TB incidence in South Africa would remain high. This was demonstrated in the no-HIV counterfactual scenario, in which there were an estimated 235 cases per 100,000 population in 2019. This rate is much higher than the estimated TB incidence for industrialized regions, such as Europe and America (World Health Organization, 2019). The high TB burden in the HIV-negative population indicates other underlying factors that drive the epidemic (i.e., low rates of diagnosis and risk factors that increase susceptibility to TB disease).

The provision of ART substantially impacted TB incidence; in 2019, it led to a 20% reduction. The benefits of ART in reducing incidence (Dye and Williams, 2019; Nanoo *et al.*, 2015; Surie *et al.*, 2018) depend on CD4 count and duration of ART (Lawn *et al.*, 2011)— individuals who are HIV-positive who initiate ART earlier at higher CD4 counts and stay on ART for longer experience the greatest benefits of ART. In the model, the effect of ART on reducing TB incidence increased during the mid-2000s, when access to ART expanded in South Africa. Over time, the CD4 count threshold at which individuals can start ART has increased (Meintjes *et al.*, 2017), and average ART durations have increased, consequently contributing to the substantial reduction in the population-level TB incidence.

Intensified TB screening also led to significant declines in TB incidence. Between 2005 and 2012, South Africa scaled-up efforts to identify TB cases and testing rates doubled (Nanoo *et al.*, 2015). As a result, there were rapid reductions in TB incidence owing to increased TB screening during this period. In 2019, increased screening led to a 28% reduction in TB incidence.

The reasons for DOTS having minimal impact on TB may include high ongoing *Mycobacterium tuberculosis* transmission rates, high prevalence of substantial risk factors, such as HIV, which increase progression to disease, and the emergence of resistant TB (De Cock and Chaisson, 1999; Wood *et al.*, 2011). Lastly, we have only considered one component of the DOTS strategy; considering other aspects could have led to a larger impact. Nonetheless, our findings of the minimal impact of DOTS align with studies that suggested that DOTS would have minimal impact in settings with a high HIV burden (Dowdy and Chaisson, 2009; Dye *et al.*, 1998).

The small population-level impact of IPT on TB incidence in individuals who are HIV-positive is consistent with other epidemiologic analyses, which attribute the limited impact to the low implementation of IPT in South Africa (Dye and Williams, 2019). However, other reasons may be because this HIV-positive population has a high risk of progression to disease or because IPT does not necessarily cure latent *Mycobacterium tuberculosis* infection in individuals who are HIV-positive (Houben *et al.*, 2014). In addition, there appears to be minimal protection from IPT after IPT discontinuation or completion. Thus, the short duration of IPT protection might explain the relatively modest population-level impact in South Africa (Churchyard *et al.*, 2014a). Another possible reason may be that ART eligibility criteria have changed over time. In recent years, more people have started ART at higher CD4 counts. Those starting ART at higher CD4 counts stand to benefit less from IPT.

Our findings regarding the small effect of Xpert MTB/RIF on TB incidence align with studies that found no significant effect of Xpert MTB/RIF on TB mortality (Di Tanna *et al.*, 2019). It has been suggested that reductions in empirical treatment offset the positive effect of Xpert MTB/RIF (Theron *et al.*, 2014a). The introduction of Xpert MTB/RIF has increased the number of microbiologically confirmed diagnoses; however, this has not equated to more new diagnoses because many cases were diagnosed empirically before adopting Xpert MTB/RIF. In addition, there was more culture testing in those testing negative under microscopy than under Xpert MTB/RIF (McCarthy *et al.*, 2016). Another modeling group, which had initially estimated a substantial positive impact of Xpert MTB/RIF on health outcomes, conducted a reanalysis accounting for empirical treatment and the sensitivity and specificity of diagnostic algorithms (Menzies *et al.*, 2015). The revised analysis found a reduction in the benefits, with 70% fewer disability-adjusted life years averted due to Xpert MTB/RIF (Menzies *et al.*, 2015).

We implemented a detailed diagnostic algorithm that estimated true- and false-positives from microbiological diagnoses. We also considered the proportions of individuals who initiate treatment empirically, as informed by South African pragmatic trials and operational studies. As a result, we showed that Xpert MTB/RIF has indeed led to a reduction in the number of individuals without TB who started treatment (by 56%) and reduced the number of individuals who started treatment on an empirical basis (by 28%). Xpert MTB/RIF possibly has other benefits, such as reducing the time to diagnosis and time to treatment initiation; however, we did not model the effect of Xpert MTB/RIF on these endpoints. Nonetheless, we assumed that loss to follow-up before treatment initiation was lower when Xpert MTB/RIF compared with smear microscopy testing was used (supplementary material). We did not model other benefits of Xpert MTB/RIF, such as the ability to detect drug-resistant TB (Di Tanna *et al.*, 2019).

This study was subject to several limitations. First, we only considered the adult population (aged 15 years and older). Second, owing to the lack of data on the national rollout of Xpert MTB/RIF, we relied on expert opinion regarding Xpert MTB/RIF implementation. Third, it was difficult to quantify the extent of empirical treatment before introducing Xpert MTB/RIF due to the lack of data and studies to inform our assumptions. Fourth, our model does not distinguish between symptomatic and asymptomatic TB. However, we made assumptions about the prevalence of symptoms for modeling screening algorithms. Fifth, our model did not fit the number of treatment initiations data very well, particularly in the earlier years of the ETR data, before 2010. The model estimates for treatment initiations peaked earlier than the ETR data, for both males and females, and it overestimated the treatment initiations in males. This may be because, in earlier years, there was greater under-reporting in the ETR data. Lastly, this analysis only focused on the past impact of interventions implemented in South Africa. Thus, we did not explore the potential impact of new interventions or improvements to current interventions.

To the best of our knowledge, this is the first comprehensive retrospective assessment of the impact of HIV and multiple TB interventions on the South African TB burden at a national level. This study demonstrated the tremendous effect HIV has had on TB incidence and mortality. However, even in the HIV-negative population, the TB incidence remains unacceptably high. The South African TB program has made notable efforts that have led to a significant reduction in TB incidence and mortality. Further modeling studies are needed to identify the changes to current programs that are required to accelerate these reductions in future.

## Author contributions

MK, LJ, and AB contributed to the study conceptualization, analysis, and interpretation of the results. LJ and MK wrote the code for the mathematical model. LJ and AB were the study supervisors. MO curated the electronic tuberculosis register data and contributed to interpretation of results. MK wrote the first manuscript draft, and all authors critically reviewed versions of the manuscript and agreed on the final version to be submitted for publication. All data used in this study were drawn from publicly available data sources, which are described and reported in the supplementary material.

## Funding

MK received PhD funding from the Fogarty International Center of the National Institutes of Health (D43 TW010559), the South African Department of Science and Technology/National Research Foundation Centre of Excellence in Epidemiological Modelling and Analysis, and the International Epidemiology Databases to Evaluate AIDS (National Institutes of Health, UO1AI069924). LJ was supported by the Bill and Melinda Gates Foundation (019496). The content of this manuscript is solely the responsibility of the authors and does not necessarily represent the views of the funders.

## Ethical approval statement

Not applicable. All data used in this study were drawn from publicly available data sources which are described and reported in the supplementary material.

## Declaration of competing interests

The authors have no competing interests to declare.

## Appendix Supplementary materials

Image, application 1
