# Supplementary material for: The impact of HIV and tuberculosis interventions on South African adult tuberculosis trends, 1990-2019: a mathematical modeling analysis
Source: Int J Infect Dis. 2022 Sep;122:811–9. doi: 10.1016/j.ijid.2022.07.047 (PMC9439958; doi:10.1016/j.ijid.2022.07.047)
Supplement: Supplementary file 1 [file mmc1.docx]

**The impact of HIV and tuberculosis interventions on South African adult tuberculosis trends, 1990-2019: a mathematical modelling analysis**

**[Supplementary material]**

Mmamapudi Kubjane^1^, Muhammad Osman^2,3^, Andrew Boulle^1,4^, Leigh F. Johnson^1^

^1^Centre for Infectious Disease Epidemiology and Research, School of Public Health and Family Medicine, University of Cape Town, South Africa.

^2^Desmond Tutu TB Centre, Department of Paediatrics and Child Health, Faculty of Health Sciences, Stellenbosch University, Cape Town, South Africa.

^3^School of Human Sciences, Faculty of Education, Health and Human Sciences, University of Greenwich, United Kingdom.

^4^Western Cape Provincial Department of Health, South Africa

List of contents

[1. Overview 5](#_Toc108581370)

[2. Initial conditions 7](#_Toc108581371)

[2.1 Latent tuberculosis infection 7](#_Toc108581372)

[2.2 Initial active tuberculosis profiles 8](#_Toc108581373)

[2.3 The initial proportion of individuals previously treated for active tuberculosis 9](#_Toc108581374)

[3. Estimating the proportion of new active tuberculosis cases that are smear-positive 9](#_Toc108581375)

[4. Modelling tuberculosis transmission 9](#_Toc108581376)

[4.1 The transmission probability per daily contact 10](#_Toc108581377)

[4.2 Infectiousness in smear-positive and smear-negative active TB 10](#_Toc108581378)

[4.3 Social mixing patterns 10](#_Toc108581379)

[5. Modelling the tuberculosis natural history and the effects of human immunodeficiency virus and antiretroviral therapy 11](#_Toc108581380)

[5.1 Progression to active tuberculosis disease: fast progression and reactivation 11](#_Toc108581381)

[5.2 Partial immunity 12](#_Toc108581382)

[5.3 Natural recovery (untreated tuberculosis) 13](#_Toc108581383)

[5.4 Untreated active tuberculosis mortality 13](#_Toc108581384)

[6. Modelling the tuberculosis diagnostic pathway 14](#_Toc108581385)

[6.1 Modelling tuberculosis health-seeking patterns 16](#_Toc108581386)

[6.2 Specimens submitted for microbiological testing 18](#_Toc108581387)

[6.3 Sensitivity and specificity of diagnostic algorithms 20](#_Toc108581388)

[6.4 Treatment initiation and initial loss to follow-up 22](#_Toc108581389)

[6.5 Modelling empirical treatment 22](#_Toc108581390)

[7. Modelling tuberculosis treatment and treatment outcomes 24](#_Toc108581391)

[7.1 Modelling cure and failure 28](#_Toc108581392)

[7.2 Modelling treatment discontinuation 28](#_Toc108581393)

[7.3 Modelling tuberculosis deaths on treatment 28](#_Toc108581394)

[8. Tuberculosis recurrence 31](#_Toc108581395)

[8.1 Short-term post-treatment 31](#_Toc108581396)

[8.2 Long-term post-treatment 31](#_Toc108581397)

[9. Modelling the effect of isoniazid preventative therapy 32](#_Toc108581398)

[9.1 Isoniazid preventative therapy initiation by LTBI/TST status 32](#_Toc108581399)

[9.2 Isoniazid preventative therapy initiation by CD4 count 32](#_Toc108581400)

[9.3 Isoniazid preventative therapy initiation by ART status 32](#_Toc108581401)

[9.4 Isoniazid preventative therapy completion/drop-out 33](#_Toc108581402)

[9.5 Effectiveness of isoniazid preventative therapy 33](#_Toc108581403)

[10. Modelling the effect of tuberculosis risk factors on tuberculosis incidence 34](#_Toc108581404)

[10.1 Poorly controlled diabetes (HbA1c > 6.5%) 34](#_Toc108581405)

[10.2 Underweight (BMI <18.5 kg/m²) 34](#_Toc108581406)

[10.3 Tobacco smoking 35](#_Toc108581407)

[10.4 Alcohol abuse 35](#_Toc108581408)

[10.5 Combined effect of TB risk factors 36](#_Toc108581409)

[11. Calibration data sources and defining likelihoods 37](#_Toc108581410)

[11.1 The likelihood for recorded number of tuberculosis deaths 38](#_Toc108581411)

[11.2 The likelihood for expected tuberculosis deaths in people living with HIV 41](#_Toc108581412)

[11.3 The likelihood for recorded number of tuberculosis cases initiated on treatment 43](#_Toc108581413)

[11.4 The likelihood for tuberculosis deaths in the electronic tuberculosis treatment register 44](#_Toc108581414)

[11.5 The likelihood for HIV prevalence in the electronic tuberculosis register 44](#_Toc108581415)

[11.6 The likelihood for the numbers of microbiological tuberculosis tests performed 45](#_Toc108581416)

[11.7 The likelihood for the prevalence of bacteriologically confirmed active tuberculosis 45](#_Toc108581417)

[11.8 Generating posterior distributions 46](#_Toc108581418)

[12. Results from model calibration 47](#_Toc108581419)

[12.1 Comparison of prior and posterior distributions 47](#_Toc108581420)

[12.2 Calibration graphs 48](#_Toc108581421)

[13. Comparison with other model estimates 55](#_Toc108581422)

[14. Additional figures: impact of interventions on tuberculosis mortality 59](#_Toc108581423)

[15. Additional figures: effect of Xpert MTB/RIF on diagnostic pathway outcomes 59](#_Toc108581424)

[16. Sensitivity analysis for latent TB prevalence assumptions 61](#_Toc108581425)

[17. Additional figures: TB incidence and mortality by different model experiments 62](#_Toc108581426)

[18. Additional figures: Sensitivity analysis – correlation coefficients for model input parameters and model outcomes 63](#_Toc108581427)

[19. Additional figures: Sensitivity analysis – pairwise correlations between model parameters 65](#_Toc108581428)

[20. Additional figures: Sensitivity analysis – scatter plots of estimated number of new TB cases and deaths and model parameters 66](#_Toc108581429)

[21. References 72](#_Toc108581430)

**List of tables**

[Table 1: Changes in average CD4 count after ART initiation 6](#_Toc108581431)

[Table 2: Latent tuberculosis prevalence in HIV-negative individuals 7](#_Toc108581432)

[Table 3: Initial prevalence of tuberculosis by age and sex in an HIV-negative population 8](#_Toc108581433)

[Table 4: Initial prevalence (%) of previous tuberculosis 9](#_Toc108581434)

[Table 5: Age- and sex-stratified proportions of social contacts in different age and sex groups, and mean contact rates 11](#_Toc108581435)

[Table 6: Proportions of patients with TB symptoms who are screened and submit sputum specimens 18](#_Toc108581436)

[Table 7: Recorded numbers of laboratory confirmed tuberculosis cases by year 20](#_Toc108581437)

[Table 8: Utilisation of GeneXpert MTB/RIF as a first-line diagnostic test in South Africa 20](#_Toc108581438)

[Table 9: Assumptions on follow-up tests by culture 21](#_Toc108581439)

[Table 10: Assumed sensitivity and specificity of diagnostic tests by smear-status 22](#_Toc108581440)

[Table 11: Definitions of data used for treatment outcomes as per the electronic tuberculosis register 24](#_Toc108581441)

[Table 12: Electronic Tuberculosis Register Treatment outcomes by year (2004 – 2016) sex and HIV-status expressed as proportions 26](#_Toc108581442)

[Table 13: Electronic Tuberculosis Register Treatment outcomes by year (2004 – 2016), sex and HIV-status expressed as rates 27](#_Toc108581443)

[Table 14: Average treatment discontinuation and death rates by HIV status, based on the ETR 28](#_Toc108581444)

[Table 15: Odds ratios for mortality in the 2+ and 3+ categories, relative to the <2+ category 29](#_Toc108581445)

[Table 16: Isoniazid Preventative Therapy eligibility, requirement, and duration 32](#_Toc108581446)

[Table 17: Number of HIV-infected new eligible individuals initiated on isoniazid preventative therapy by province 33](#_Toc108581447)

[Table 18: Age- and sex-specific prevalence (%) of risk factors: HbA1c > 6.5%, underweight, alcohol abuse and tobacco smoking 34](#_Toc108581448)

[Table 19: Cumulative age- and sex- effect of selected tuberculosis risk factors HbA1c > 6.5%, underweight, smoking and alcohol abuse 36](#_Toc108581449)

[Table 20: Summary of model parameters with prior means and standard deviations, posterior means and 95% confidence intervals from the previous calibration analsyis, and summary of those varried in this present analysis 37](#_Toc108581450)

[Table 21: Number of tuberculosis deaths and adjustments 40](#_Toc108581451)

[Table 22: Expected number of tuberculosis deaths in people living with HIV 41](#_Toc108581452)

[Table 23: Number of tuberculosis cases initiated on treatment by year, sex and HIV status 43](#_Toc108581453)

[Table 24: Recorded numbers of microbiological tuberculosis test performed by year 45](#_Toc108581454)

[Table 25: Comparison of prior and posterior distributions for model parameters 47](#_Toc108581455)

[Table 26: Comparison of 2019 tuberculosis disease burden estimates by the three models: Thembisa, IHME and WHO 56](#_Toc108581456)

[Table 27: Comparison of 2019 tuberculosis disease burden estimates by the three models, by sex: Thembisa, IHME and WHO 56](#_Toc108581457)

[Table 28: General differences between Thembisa TB/HIV, the Institute for Health Metrics and Evaluation and the World Health Organization for 2019 TB burden estimates 58](#_Toc108581458)

[Table 29: The effect of Xpert MTB/RIF on laboratory diagnoses and treatment initiations 59](#_Toc108581459)

[Table 30: Correlation coefficients for model input parameters varied in the calibration process, and specified outcomes 64](#_Toc108581460)

[Table 31: Pair-wise correlation coefficients between model parameters 65](#_Toc108581461)

**List of figures**

[Figure 1: The tuberculosis natural history model structure 5](#_Toc108681735)

[Figure 2: The Thembisa HIV model structure 6](#_Toc108681736)

[Figure 3: Illustration of the modelled tuberculosis diagnostic pathway 14](#_Toc108681737)

[Figure 4: Simplified diagnostic algorithm considered in the model 21](#_Toc108681738)

[Figure 5: Flow for cleaning mortality data from the vital register 39](#_Toc108681739)

[Figure 7: Recorded number of tuberculosis deaths (adjusted) and model estimated deaths in males 15+ 50](#_Toc108681740)

[Figure 8: Recorded number of tuberculosis deaths (adjusted) and model estimated deaths in females 15+ years 50](#_Toc108681741)

[Figure 9: Expected tuberculosis deaths in people living with HIV and model estimated tuberculosis deaths in HIV-positive individuals 51](#_Toc108681742)

[Figure 10: Recorded number of tuberculosis cases initiated on treatment (adjusted) and model estimated tuberculosis cases on treatment in males 15+ years 51](#_Toc108681743)

[Figure 11: Recorded number of tuberculosis cases initiated on treatment (adjusted) and model estimated tuberculosis cases on treatment in females 15+ years 52](#_Toc108681744)

[Figure 12: Proportion of tuberculosis deaths recorded in the electronic tuberculosis treatment register and model estimates for proportion of tuberculosis deaths on treatment for males 15+ years 52](#_Toc108681745)

[Figure 13: Proportion of tuberculosis deaths recorded in the electronic tuberculosis treatment register and model estimates for proportion of tuberculosis deaths for females 15+ years on treatment 53](#_Toc108681746)

[Figure 14: HIV prevalence in the electronic tuberculosis register and model estimated HIV prevalence in adults on treatment 53](#_Toc108681747)

[Figure 15: The numbers of microbiological tuberculosis tests performed and model estimates for microbiological tests performed 54](#_Toc108681748)

[Figure 16: The prevalence of bacteriologically confirmed active tuberculosis and model estimated prevalence of tuberculosis in males 15+ years 54](#_Toc108681749)

[Figure 17: The prevalence of bacteriologically confirmed active tuberculosis and model estimated prevalence of tuberculosis in females 15+ years 55](#_Toc108681750)

[Figure 18: The impact of programmatic interventions on tuberculosis deaths. 59](#_Toc108681751)

[Figure 19: Adult TB trends under different assumptions about the effect of age on initial LTBI prevalence 62](#_Toc108681752)

[Figure 20: TB incidence and mortality by different counterfactual scenarios 63](#_Toc108681753)

[Figure 21: Scatter plots to show the relationship between estimated new TB cases and model parameters 67](#_Toc108681754)

[Figure 22: Scatter plots to show the relationship between estimated TB mortality and model parameters 70](#_Toc108681755)

## Overview

We developed an age- and sex-structured deterministic compartmental model of the TB and HIV epidemics for the South African adult population (ages 15 years and older). The core TB states were modelled following conventions used by previous modelling studies (1–3). We considered the following epidemiological states: susceptible, latent tuberculosis infection (LTBI), active TB smear-positive, active TB smear-negative, receiving treatment, and two post-TB treatment states – recent (six months after treatment) and long-term (more than six months after treatment), as shown in Figure 1.

Figure 1: The tuberculosis natural history model structure


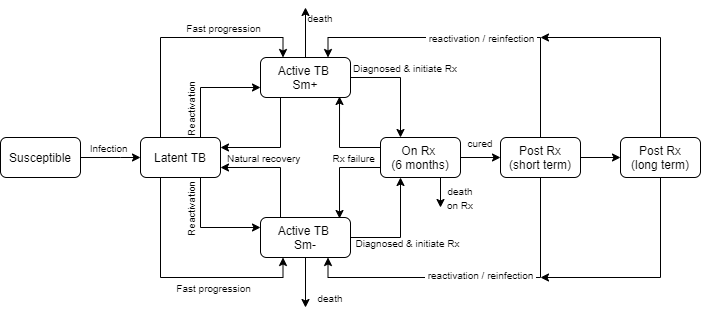


TB = tuberculosis. Rx = treatment. Sm+ = smear-positive. Sm- = smear negative.

Although the model produces estimates for individuals with 15 years and older, it simulates TB transmission and the natural history from age 10 years. We assume a fixed proportion of children are latently infected with TB on reaching age 10. The prevalence of LTBI is based on an age-dependent function described in **section 2**, and the LTBI prevalence at the age of ten years is $F_{ltbi}\left( 10 \right)=0.47$, which is where we then start simulating the potential for disease progression and transmission.

The TB transmission process and associated parameters are described in **section 4**. Following infection, individuals progress to TB disease through fast progression or remain infected with latent TB and may develop TB disease through reactivation or reinfection (described in **section 5**). Individuals with LTBI have partial immunity, which affords them a reduced risk of fast progression to TB disease following reinfection. Individuals with TB disease were further categorised by smear status – smear-positive or smear-negative. In these TB disease states, individuals may die or recover naturally and return to the LTBI state. Active TB individuals can also be linked to TB care, which depends on attending a health facility, being screened, being diagnosed (empirically or microbiologically), and ultimately initiating treatment; the TB diagnosis and treatment initiation process are explained in **section 6**. Complete treatment is assumed to last an average of six months. Those on treatment can experience the following outcomes: a) cure; b) failure, after which they will return to the active TB states; c) discontinuation of treatment, of which a proportion will return to TB disease state and the remainder to the recovered state; and d) death. These treatment outcomes are further described in **section 7**. Following treatment completion, we consider two post-TB treatment states that depend on the time since treatment cure/completion, which account for recurrent TB episodes through reinfection and relapse (described in **section 8**).

To incorporate the effect of HIV and ART, the TB model was integrated into the Thembisa HIV model. The Thembisa model simulates the South African population, by age and sex, at monthly time steps from 1985 (4). The structure of the HIV model is given in Figure 2, and a detailed description of the HIV model (Thembisa 4.3) has been published (5). Briefly, in this model, HIV-infected sub-populations are stratified by HIV testing history, CD4 count and antiretroviral treatment duration. For those diagnosed with HIV, there is an indicator for ART initiation, and the CD4 count levels are used to represent the HIV stage or baseline CD4 at which individuals initiated ART. For those not on ART yet, the CD4 compartment represents their CD4 count at that given point (*current CD4*). For those on ART, the duration that they have been on ART treatment is also tracked, and for each duration compartment an average CD4 count is calculated, which depends on the baseline CD4 count (5). The descriptions of how HIV and ART affect the TB natural history (**section 5**); and health-seeking patterns (**section 6**) are provided in the respective sections.

Additionally, in the model, we have incorporated the effect of isoniazid preventative therapy (described in **section 9)**. We have also explored the effect of selected TB risk factors on TB incidence in **section 10**, as these are particularly relevant in representing sex differences in TB incidence. **Section 11** describes the approach and data sources we used to calibrate the model. Before this current analysis, an initial calibration analysis was performed to identify the transmission and natural history parameters that best fit the South African TB data (details in section 11). The parameters varied in this current analysis were mostly related to the effects of HIV, ART and control interventions; these are described in detail in section 11, Table 20. In **section 12,** the model calibration results are presented.

**Section 13** compares the Thembisa TB/HIV model estimates to those produced by the World Health Organisation (WHO) and the Institute for Health Metrics and Evaluation (IHME). Lastly, in **sections 14** to **section 20** we present additional figures to support this current analysis.

Figure 2: The Thembisa HIV model structure

*
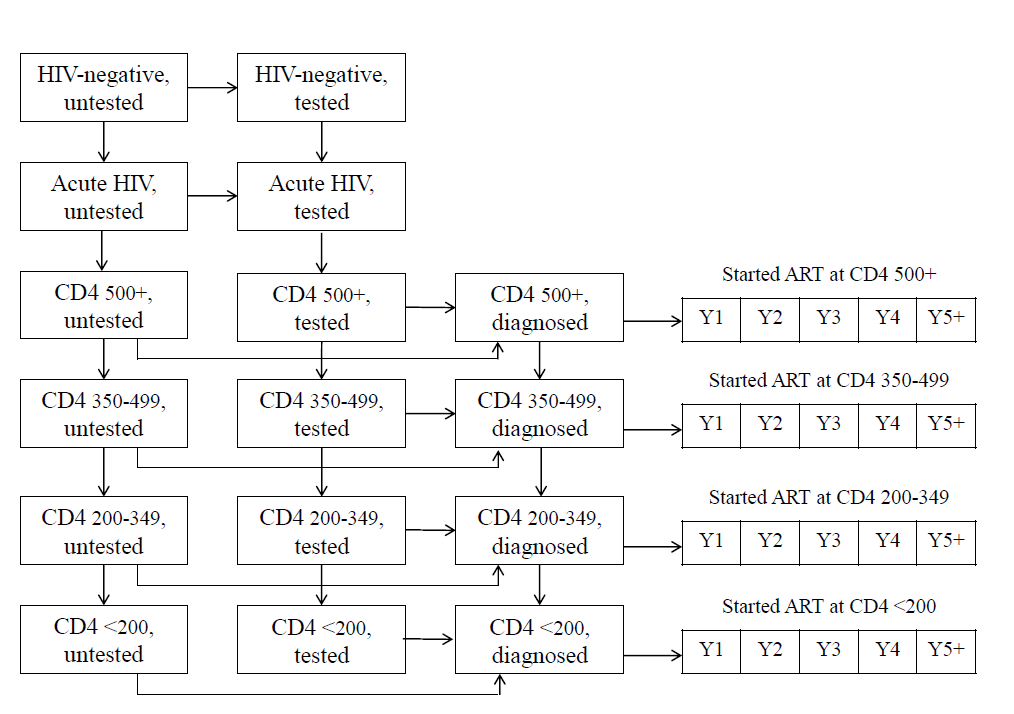
*

Figure from Johnson & Dorrington (5)

In the Thembisa HIV model, an average CD4 count in HIV-positive individuals who are not on ART is assumed for each of four CD4 stages, and is given in the first column in the table below (Table 1). CD4 counts in HIV-positive individuals receiving ART are dependent on the baseline CD4 count when they initiated ART and the number of years they have been receiving ART. These are shown in Table 1 below, and have been described and derived previously (6).

For the purpose of setting later assumptions about the effect of HIV on TB, it is necessary to assume an average CD4 count in the HIV-negative population. We set the average CD4 count in the HIV-negative population at 1000 cells/μl, based on Williams *et al*., who reported a range of CD4 levels between 699 and 1244 cells/μl (7). We assumed CD4 counts in patients on ART do not rise above 1000.

Table 1: Changes in average CD4 count after ART initiation

| **Years since ART initiation** | | | | | | |
| --- | --- | --- | --- | --- | --- | --- |
|  | **Not on ART** | **1** | **2** | **3** | **4** | **+5** |
| **Baseline CD4 <200** | 100 | 297 | 383 | 426 | 486 | 512 |
| **Baseline CD4 200-349** | 275 | 450 | 539 | 600 | 703 | 770 |
| **Baseline CD4 350-499** | 425 | 564 | 658 | 738 | 882 | 987 |
| **Baseline CD4 500+** | 610 | 705 | 805 | 909 | 1000 | 1000 |

Source: Johnson & Dorrington (6)

## Initial conditions

This section describes the assumptions we made about the proportion of the population with LTBI and active TB at the start of the simulation in 1985. All these initial assumptions are made for an HIV-negative population because HIV prevalence was very low in 1985.

### Latent tuberculosis infection

In the model, we set the initial LTBI prevalence in the HIV-negative population using data from the CORTIS study (8). In this study, the QuantiFERON-TB Gold Interferon Gamma Release Assay (IGRA) assays were used to detect LTBI (Table 2) (8). However, we note that the relatively low sensitivity of IGRAs implies a high likelihood of missing true IGRA positives. Therefore, to approximate the proportion of true LTBI individuals given these imperfect diagnostic tools and the lack of a standard gold test for latent infection, we adjust the given prevalence using the sensitivity and specificity of QuantiFERON-TB Gold.

Table 2: Latent tuberculosis prevalence in HIV-negative individuals

| **Age Group** | **N** | **Unadjusted IGRA+ prevalence** $\boldsymbol{p}_{\mathbf{1}}$ | **Adjusted prevalence** $\boldsymbol{p}_{\mathbf{2}}$ |
| --- | --- | --- | --- |
|  |  |  |  |
| 15-24 | 1249 | 703 (56.3) | 70.7 |
| 25-34 | 1062 | 748 (70.4) | 89.7 |
| 35-44 | 379 | 271 (71.5) | 91.2 |
| 45+ | 223 | 164 (73.5) | 93.9 |
| Total | 2913 | 1886 (64.7) | 86.4 |

Source: CORTIS study (8). <https://www.clinicaltrials.gov/ct2/show/NCT02735590>

We suppose the unadjusted LTBI prevalence, as measured by IGRA (Table 2), can be expressed as

$p_{1}=p_{2}Se+(1- p_{2})(1-Sp)$,

where $p_{2}$ is the adjusted (true) LTBI prevalence, and $Se$ and $Sp$ are the sensitivity and specificity of QuantiFERON-TB Gold, respectively. The assumed sensitivity and specificity of QuantiFERON-TB Gold were 0.78 and 0.96 respectively (9). Then we have

${p_{2}=(p}_{1}-(1-Sp))/(Se+Sp -1$).

We fitted an exponential function to the adjusted prevalence $p_{2}$, and thus the prevalence of LTBI can be given by the expression dependent on age: $F_{ltbi}\left( age \right)=0.98\left( 1-e^{-0.066age} \right)$.

Limitations associated with using these IGRA-based data include a greater likelihood to underestimate the proportion of true IGRA-positives because of the low sensitivity of the tool. Another limitation of using these IGRA-based data to initiate the LTBI profiles in the model is that these are recent data and are possibly not a true reflection of LTBI prevalence in 1985. Nonetheless, we justify this with the fact that the proportions were from an HIV-negative population (and hence may represent the era where HIV prevalence was very low in South Africa) (8). Also, the study was conducted across multiple sites in South Africa and thus giving a better representation of the country’s LTBI prevalence. In comparison, other existing earlier data on LTBI prevalence were conducted in smaller and specific population group (i.e., adolescents, gold miners, health care workers) (10–14) making them less representative nationally. Additionally, most of these earlier studies relied on TST, which has poor specificity, especially given the fact that South Africa is a country with high BGC vaccination coverage.

### Initial active tuberculosis profiles

There is also limited data on the prevalence of active TB earlier than 1990. The available TB burden data from reports only dates from the early 2000s. We used WHO estimates to initialise the prevalence of TB in our model. The TB prevalence in 1990 was estimated to be 490 per 100 000 (15). The initial TB prevalence was set in 1985 in an HIV-negative population. We used a Cape Town-based population-based study that assessed the 2009 age-specific TB notification rates (TB incidence) stratified by HIV status (16) to set the assumed relative levels of TB prevalence by age.

As with most routinely collected data, the notification data used to estimate the proportion of active TB cases is likely to be incomplete or affected by misclassification of TB status or duplication of individuals. Also, these data only represent one geographical setting - Western Cape. However, to initiate the model, we do not expect these initial assumptions to affect model outputs significantly.

As the literature suggests, males carry a higher burden of TB compared to females. The male to female ratio of the TB burden ranges between 1.5 and 2.1 across different geographical regions (17–19). In the model, we set the initial prevalence of TB by age and sex, represented by the proportions in Table 3.

These were computed using the following data: 1) the estimated 1990 South African TB prevalence by the WHO (15), 2) the Cape Town TB prevalence in an HIV-uninfected population (16) and 3) the ratio of male to female TB prevalence (20). For the age groups 15 – 75 years, we assume the prevalence of active TB in males to be 50% higher than that in females (20). We assume that the overall prevalence $\bar{p}=0.5 \left( p_{0}+p_{1} \right)$, where $p_{0}$ is the prevalence in males and $p_{1}$ is the prevalence in females. We then have $p_{0}= 1.5\times\bar{p}/(0.5\times(1 + 1.5))$ and $p_{1}=\bar{p}/(0.5\times(1 + 1.5))$.

Table 3: Initial prevalence of tuberculosis by age and sex in an HIV-negative population

| Age | HIV negative  Population, a) | Number of active TB cases, b) | The proportion of active TB, c) | % TB prevalence in males, p_0_ | % TB prevalence in females, p_1_ |  |
| --- | --- | --- | --- | --- | --- | --- |
| 15–19 | 301447 | 988 | 0.004 | 0.005 | 0.003 |  |
| 20–24 | 306210 | 1693 | 0.007 | 0.008 | 0.006 |  |
| 25–29 | 287160 | 1381 | 0.006 | 0.007 | 0.005 |  |
| 30–34 | 251725 | 1015 | 0.005 | 0.006 | 0.004 |  |
| 35–39 | 224038 | 1042 | 0.006 | 0.007 | 0.005 |  |
| 40–45 | 179524 | 1053 | 0.007 | 0.009 | 0.006 |  |
| 45–49 | 171395 | 1076 | 0.008 | 0.009 | 0.006 |  |
| 50–54 | 156238 | 829 | 0.007 | 0.008 | 0.005 |  |
| 55–59 | 126738 | 571 | 0.006 | 0.007 | 0.005 |  |
| 60–64 | 104718 | 315 | 0.004 | 0.005 | 0.003 |  |
| 65–69 | 77231 | 195 | 0.003 | 0.004 | 0.003 |  |
| 70–74 | 52120 | 72 | 0.002 | 0.002 | 0.001 |  |
| 75+ | 62485 | 97 | 0.002 | 0.002 | 0.002 |  |
| Total | 3241508 | 12508 | 0.005 | 0.006 | 0.004 |  |

a) and b) are based on the Wood *et al* study (16).

c = b/a × (0.0049÷0.0039)=$\bar{p};$ 0.0049 is the 1990 WHO prevalence of TB (15); and 0.0039 (= 12508/32 241 508) is the average TB prevalence from the Wood *et al,* study (16). $p_{0}=\frac{3\bar{p}}{2.5}$, $p_{1}=\frac{2\bar{p}}{2.5}$.

### The initial proportion of individuals previously treated for active tuberculosis

The initial proportions of individuals previously treated for active TB were based on self-reported data on prior TB diagnosis from the South African Demographic and Health Survey 1998 (SADHS) (21). The initial prevalence is specified by age and sex as reported in the SADHS (Table 4).

Table 4: Initial prevalence (%) of previous tuberculosis

| **Age category** | **Males** | **Females** |
| --- | --- | --- |
| 15-25 | 0.8 | 1.1 |
| 25-34 | 2.1 | 1.8 |
| 35-44 | 4.1 | 2.0 |
| 45-54 | 5.2 | 2.6 |
| 55-64 | 4.1 | 2.2 |
| 65 and older | 4.4 | 3.1 |
| Overall | 2.9 | 2.0 |

## Estimating the proportion of new active tuberculosis cases that are smear-positive

Our model requires assumptions about the proportion of incident TB cases that are smear-positive, but most studies report the proportion of newly diagnosed/treated TB cases that are smear-positive. We expect the proportion of treated active TB cases that are smear-positive to be higher than the proportion of incident TB cases that are smear-positive due to the delays in TB diagnosis being greater for smear-negative TB than for smear-positive TB.

We used the Gupta *et al*. study to set the proportions of incident TB cases that are smear-positive, by HIV status (22). The study assessed how HIV and CD4 count affected TB disease site, smear status and overall laboratory confirmation of TB cases. The study was based on the 2009 electronic TB register for Cape Town. From this study, the proportion of HIV-negative individuals who were smear-positive was 0.523; among HIV-positive individuals, the proportion was 0.326. The proportion of HIV-negative individuals with no laboratory confirmation for TB diagnosis was 0.374 (22). Given these data, we previously set the prior mean for the proportion of smear-positive individuals at 0.52. We further supposed an upper limit of 0.84 (=0.523/(1 – 0.374)), where 0.374 is the proportion of those with unconfirmed smear status. This upper limit was based on assuming that the smear-positive proportion in those with missing smear results is the same as that in those with recorded smear results. As such, we assigned a Beta (12.46;11.50) prior distribution, with a mean of 0.52 and standard deviation of 0.1, for this parameter. In this current analysis, we fixed this parameter at 0.51, which was the posterior (mean=0.51, 95% CI 0.48 – 0.54) in a previous calibration analysis. Lastly, we set the proportion of smear-positives in HIV-positive patients to be 0.62 times that in HIV-negative individuals (0.326/0.523) (22).

## Modelling tuberculosis transmission

We define the force of infection ($\lambda_{ly}$) as the daily rate at which a susceptible individual of sex $l$ in age group $y$ gets infected at each step. The number of susceptible individuals who get infected in each time step is represented by the expression below:

$${N_{ly}\lambda}_{ly}=\sum_{g} \sum_{x} {c_{gx}\rho}_{gxly}\sum_{i\epsilon\left\{ Ω_{gx} \right\}} I_{i}\beta$$

Where $c_{gx}$ is the frequency of contacts per day (daily rate) that individuals of sex $g$ and age group $x$ have with other individuals (Table 5). This crude contact rate is obtained directly from data on the frequency of close contacts in a South African study (23). Also obtained directly from social mixing pattern data, $\rho_{gxly}$ represents the proportion of contacts between an individual of sex $g$ and age $x$, with individuals of sex *l* and age *y* (Table 5). Next, $I_{i}$ represents the relative infectiousness of individual $i$, which depends on smear positivity; $Ω_{gx}$ is the subset of the active TB population with age group *x* and sex $g$. Then $N_{ly}$ is the number of individuals of sex *l* and age *y.* Lastly, $\beta$ represents the probability of transmission per contact if a smear-positive individual has contact with a susceptible individual.

### The transmission probability per daily contact

We allow for the transmissibility of smear-positive TB to change over time, because as treatment delays reduce, we would expect fewer TB patients to progress to the more advanced (and more infectious) disease stages prior to treatment. To model transmission, we define $\beta\left( t \right)$ to be the transmission probability per daily contact between a smear-positive TB case and a susceptible individual in year $t$*,* and $\beta\left( 0 \right)$ is the corresponding average transmission probability in the period before 2000. The prior distribution assigned to represent the uncertainty in this transmission parameter $\beta\left( 0 \right)$ is a Gamma (1; 400) distribution with mean = 0.0025 and standard deviation = 0.0025. Although this parameter value is set arbitrarily, it was chosen based on whether the chosen value yielded reasonable transmission rates. In the initial model fitting, the parameter value was set at 0.02, however it yielded implausibly high rates of transmission, and so we selected a prior distribution with a lower mean but high variance.

We define $r_{i}$ as the ratio of the minimum infectivity (when the treatment delay is zero) to the baseline infectivity (given the treatment delay in the period before 2000). $S\left( t \right)$ represents the average smear-positive treatment delay, approximated as $U\left( t \right)/R(t)$, where $U\left( t \right)$ is the number of untreated smear-positive TB cases at the start of year *t* and $R(t)$ is the number of smear-positive TB patients who are treated in year $t$.$S\left( 0 \right)$ represents the average smear-positive treatment delay before 2000.

Because we lack data on smear grade distributions in South Africa before 2000, we conservatively estimated $r_{i}$ based on the smear grade distribution observed by Singla *et al.* (24) in an Indian population with poor treatment access. In this study, the distribution of smear-grades in TB patients were 27% for smear-grade <2+; 25% for smear-grade 2+ and 48% for smear-grade 3+ (24). We assumed the same relative levels of infectiousness by smear grade as measured by Acuña-Villaorduña *et al*., who estimated that infectivity increased by 1.45 times and 4.25 times in patients with smear grades 2+ and smear grades 3+ respectively, compared to TB patients with smear grade 1+ (25). Then we have $r_{i} = 1/(0.27 + 0.25 \times1.45 + 0.48 \times4.25) = 0.37$, on the assumption that if there was no treatment delay all smear-positive TB cases would have smear-grade <2+.

To allow this transmission probability to change with disease severity (indicated by smear-grade distributions), we assume that for $t \geq2000$,

$\beta\left( t \right)=\beta\left( 0 \right)\left( 1-\left( 1-r_{i} \right)\left[ 1-S\left( t-1 \right)/S\left( 0 \right) \right] \right)$.

### Infectiousness in smear-positive and smear-negative active TB

We rely on evidence from Andrews *et al*., who estimated the relative infectivity of smear-negative compared with smear-positive individuals to be 0.22 (95% CI 0.16-0.32) (26). We define $I_{i}$ as the relative infectiousness of individual $i$ and previously specified a Beta (41.73; 147.9) prior distribution for the uncertainty around the relative infectiousness of smear-negative TB, with a mean of 0.22 and standard deviation of 0.03 (26). In a previous calibration analysis, we estimated the posterior mean and 95% confidence interval for this parameter at 0.206 (95% CI 0.196 – 0.218). In this analysis, we fixed this parameter at this mean of 0.206.

### Social mixing patterns

The primary data source for the social mixing parameters in the model is the Dodd *et al*. study (27). The data from this study were based on a social contact survey conducted in eight communities in the Western Cape. In the study, interviewees reported contacts that occurred approximately 24 hours before the interviews. We chose these data because they provide both an age and gender stratification. However, the data is limited to age groups >18 years (28). Therefore, for ages 10-18 years, we use data from Johnstone-Robertson *et al*. (29). This Johnstone-Robertson study was also conducted in the Western Cape, although the setting was limited to one township and the data were not differentiated by sex. Based on several social mixing studies, the majority of close contacts in this age group occur in school settings among children of similar age groups (29–31). For simplicity, we assumed the sex structure in the age groups 10-18 years (Johnstone-Robertson *et al*. data (29)) would be similar to that observed in the 19-25-year age group (Dodd *et al*. data (27)).

The average contact rates across the age groups in the Johnstone-Robertson *et al.* study (29) were higher than those observed in Dodd *et al.* (27) study. This may be due to differences in study settings and definitions of "close contacts". In the Dodd *et al*. study, close contacts were defined as contacts involving a face-to-face conversation longer than a greeting and within an arm's reach (27). In Johnstone-Robertson *et al*. study, they were defined as contacts involving physical touch or those that involved a two-people conversation with three or more words in the physical presence of another person without physical touch (29). The former definition is more restrictive than the latter, hence limiting the number of contacts recorded.

In order to have consistency in the mixing patterns in our assumptions (which are based on the Dodd *et al*. study (27)) and those observed in Johnstone-Robertson *et al.* study, we applied the ratio of mean contacts for 10-17-year-olds to 18-25 year- olds in the Johnstone-Robertson *et al.* study to the 18-25-year-old group in Dodd et al (29). That is, the ratio 19.0 /15.9 = 1.2 multiplied by our study's average contact rate for the 18-25 years age group. The resulting contact rates and social mixing proportions are in Table 5 .

Table 5: Age- and sex-stratified proportions of social contacts in different age and sex groups, and mean contact rates

|  |  | **Female contactees** | | | | | **Male contactees** | | | | |  |
| --- | --- | --- | --- | --- | --- | --- | --- | --- | --- | --- | --- | --- |
|  | **Age** | **0-4** | **5_12** | **13-25** | **26-45** | **>45** | **0-4** | **5_12** | **13-25** | **26-45** | **>45** | **Mean** |
| **Female** | **10-17** | 0.016 | 0.042 | 0.494 | 0.124 | 0.021 | 0.015 | 0.027 | 0.169 | 0.081 | 0.012 | 6.84 |
|  | **18-25** | 0.052 | 0.073 | 0.269 | 0.168 | 0.09 | 0.049 | 0.046 | 0.092 | 0.11 | 0.05 | 5.70 |
|  | **26-45** | 0.058 | 0.083 | 0.155 | 0.238 | 0.07 | 0.051 | 0.058 | 0.089 | 0.145 | 0.05 | 5.40 |
|  | **>45** | 0.029 | 0.066 | 0.162 | 0.194 | 0.1 | 0.046 | 0.062 | 0.13 | 0.117 | 0.09 | 5.30 |
| **Male** | **10-17** | 0.016 | 0.029 | 0.225 | 0.091 | 0.017 | 0.015 | 0.040 | 0.438 | 0.115 | 0.015 | 6.60 |
|  | **18-25** | 0.024 | 0.032 | 0.155 | 0.119 | 0.08 | 0.022 | 0.045 | 0.302 | 0.15 | 0.07 | 5.50 |
|  | **26-45** | 0.026 | 0.042 | 0.123 | 0.166 | 0.07 | 0.029 | 0.04 | 0.124 | 0.294 | 0.09 | 4.70 |
|  | **>45** | 0.037 | 0.047 | 0.117 | 0.144 | 0.12 | 0.03 | 0.058 | 0.13 | 0.184 | 0.13 | 4.70 |

## Modelling the tuberculosis natural history and the effects of human immunodeficiency virus and antiretroviral therapy

### Progression to active tuberculosis disease: fast progression and reactivation

Following infection with LTBI, a proportion of individuals progresses directly to active TB disease (‘fast progression’). We specified the value of this proportion of fast progressors at 0.1 to be consistent with previous modelling studies (32–34).

The remainder of individuals are assumed to stay latently infected and may develop active TB disease at an annual rate through reactivation. This rate might be estimated as 0.0024, based on assuming 0.0866 as the rate of fast progression in the first year after infection and 0.028 as the relative risk of progression in the fifth year, as estimated by Vynnycky & Fine $(0.0024=0.0866\times0.028)$ (1). A prior distribution to represent the uncertainty around this reactivation rate parameter was previously specified as a Gamma (4; 1666.7) distribution. The corresponding mean and standard deviation were 0.0024 and 0.0012 respectively. In a previous calibration analysis, we estimated the posterior mean and 95% confidence interval at 0.00148 (95% CI 0.0014 – 0.00155). In this analysis, we fixed this parameter at this mean of 0.00148.

To model the effect of HIV on progression to TB disease, we consider the following parameters: 1) $\theta,$ the relative rate of TB incidence per 100 cells/μl increase in CD4 count; and 2) $\pi$ relative rate of TB incidence on ART after controlling for CD4 count.

To set the effect of CD4 count on TB disease risk ($\theta$), we use the meta-analysis by Ellis *et al*., which estimated that an increase in 100 CD4 cells/μl was associated with a 30% reduction in TB risk (IRR 0.70, 95% CI 0.53 – 0.86) (35). As such, we previously set a Beta (19.52; 7.97) prior distribution to represent the uncertainty around this $\theta$ parameter. In this analysis, we fixed this parameter at mean of 0.703. This is based on the posterior mean and 95% CI (0.703, 95% CI 0.693 – 0.712) we estimated in a previous calibration analysis.

Secondly, we use assume HIV viraemia has an effect on developing TB disease, independent of CD4 count. That is, a person with untreated HIV might be at increased risk of TB disease when compared to an HIV-negative person with the same CD4. Because we cannot measure this parameter directly, we use, as a proxy, the effect of ART on TB disease (independent of CD4) since ART generally suppresses viraemia (36,37). We set the relative rate of TB incidence while on ART, controlling for CD4 count ($\pi$), based on Fenner *et al*.’s study which estimated that unsuppressed viral load (1000-9999 compared to <1000) was associated with an increased risk of TB disease (adjusted RR 1.23, 95% CI 1.08 – 1.41) (37). Using this study (Fenner *et al*.), the assumed protective effect of ART is $\pi=1/1.23=0.81$, and a Beta (49.05; 11.51) prior distribution is specified for the uncertainty around this parameter.

In the model, the relative effect of HIV on TB incidence in HIV-positive individuals who are not on ART is represented by:

$H\left( s \right)={\pi^{-1}\theta}^{(a-s)/100}$,

and the relative effect of HIV on TB incidence in HIV-positive individuals on ART is represented by:

$A\left( s,d \right)=\theta^{\left( a - s_{d} \right)/100}$,

where

$a$: average CD4 count in HIV-negative individuals

$s$: average CD4 count in HIV-positive individuals not on ART (for a given CD4 compartment)

$s_{d}$: CD4 count for those on ART at the treatment duration $d$

$\theta:$ relative rate of TB incidence per 100-cell increases in CD4 count

$\pi$: Relative rate of TB incidence on ART (controlling for CD4).

### Partial immunity

In the model, we assume that individuals with LTBI have partial immunity and that the relative risk of developing TB is 0.21 if they are re-infected. This relative risk of developing TB is based on Andrews *et al*., who showed that individuals with LTBI had a 79% reduced risk of developing TB disease (95% CI 70-86%) (38). Therefore, we fixed this parameter for partial immunity in HIV-negative individuals (represented by parameter $y$) at a mean of 0.79.

There is a lack of data regarding partial immunity in HIV-positive individuals and the effect of ART. We extrapolate from assumptions made in the modelling study by Menzies *et al*. who assumed a 0.25 (range: 0.14 – 0.39) reduction in the risk of developing TB disease among HIV-positive individuals with CD4 counts > 350 cells/μl, and no partial immunity for the those with CD4 count < 350 cells/μl (34). In our model, the effect of HIV on partial immunity is modelled as a function of CD4 count. We set $p$, the relative rate of partial immunity against TB disease per 100-cell increase in CD4 for HIV-positive individuals at 1.1. This relative rate is applied to the parameter ($y$) representing the reduction in TB incidence in previously infected HIV-negative individuals in the model.

This partial immunity effect for HIV-positive individuals is

$I\left( s \right)={yp}^{\left( s- a \right)/100}$,

where

$a:$ average CD4 count in HIV-negative individuals.

$s:$ the current CD4 count in HIV-positive individuals

$p$: relative rate of partial immunity against TB per 100-cell increase in CD4 count, for HIV-positive individuals

$y$: the reduction in TB incidence in latently infected individuals who are HIV-negative.

### Natural recovery (untreated tuberculosis)

Studies from the pre-antibiotic era have shown that a certain proportion of patients do not die in the absence of treatment and presumably recover naturally without treatment (39). Based on Tiemersma *et al.'*s review of studies conducted during the pre-chemotherapy era, case fatality rates among untreated TB cases were estimated to be 0.7 among smear-positive and 0.2 among smear-negative individuals; the duration of active TB disease was estimated at $3.3$ years (39). As these case fatality rates represent cumulative mortality risks, we convert them into annual mortality rates in the model. For the annual mortality rate in smear-negative $(\mu_{smn})$ and smear-positive ($\mu_{smp}$) individuals, and for the annual natural recovery rate in smear-negative ($n_{smn}$) and smear-positive individuals ($n_{smp}$), we have the following relationships: duration of smear-positive TB disease is $1/( \mu_{smp}+ n_{smp})=3.3$, and the same duration is assumed for smear-negative TB. Similarly the case fatality ratio for smear-positive TB is $\mu_{smp}/ (\mu_{smp}+ n_{smp})=0.7$ and $\mu_{smn}/ (\mu_{smn}+ n_{smn})=0.2.$ Given these expressions, for smear-positive individuals, we get $\mu_{smp}=0.212\mathrm{and} n_{smp}=0.091$; for smear-negative individuals, we have $\mu_{smn}=0.061$ and $n_{smn}=0.242$ (39).

For HIV-negative individuals, we previously specified a Gamma (20.25; 225) prior distribution with mean of 0.09 and standard deviation of 0.02 for the uncertainty around the rates of natural recovery among smear-positive ($n_{smp}$). For natural recovery among smear-negative, we specified a Gamma (23.04; 96) prior distribution with mean 0.24 and standard deviation of 0.05 ($n_{smn}$). In a previous calibration analysis, we estimated the posterior means and 95% confidence intervals for the parameters $n_{smp}$ and $n_{smn}$ at 0.075 (95% CI 0.067 – 0.081) and 0.224 (95% CI 0.198 – 0.247), respectively. In this analysis, we fixed $n_{smp}$ and $n_{smn}$ at these posterior means of 0.075 and 0.224 respectively.

There is limited evidence on how HIV and ART affect natural recovery. As with partial immunity, we specified the relative rate of natural recovery dependent on CD4 count, extrapolating from a previous modelling study by Menzies et al. who assumed an annual rate of recovery of 0.2 (range: 0.15 – 0.025) in those who were HIV-negative, 0.1 (range: 0.06 – 0.16) in those with CD4 counts > 350 (34), and zero in those with a CD4 count of 350 and less.

In the model, the rate of natural recovery is modelled as follows:

$$R\left( s,d \right)={nv}^{\left( s- a \right)/100}$$

$v:$ relative rate of natural recovery per 100-cell increase in CD4 for HIV-positive individuals. We set $v=1.2$ to be roughly consistent with Menzies *et al* (34).

$n:$ natural recovery rates in smear-positive ($n_{smp})$ or smear-negative ($n_{smn}$) individuals who are HIV-negative.

$s:$ the current CD4 count in HIV-positive individuals.

### Untreated active tuberculosis mortality

As described in the section on Natural recovery, the untreated annual mortality rates in HIV-negative individuals with TB can be estimated as $\mu_{smp}=0.212$ and $\mu_{smn}=0.061$ for smear-positive and smear-negative TB respectively (39). We previously specified a prior distribution for smear-positive mortality that was Gamma (25; 117.9) with mean $0.212$ and standard deviation of 0.042. For smear-negative mortality, we similarly specified a prior distribution that was Gamma (25; 409.8), with a mean of $0.061$ and standard deviation of $0.012$.

Based on a previous calibration analysis, the resulting posterior means and 95% confidence intervals for $\mu_{smp}$ and $\mu_{smn}$ were 0.196 (95% CI 0.174 – 0.221) and 0.049 (95% CI 0.046 – 0.052), respectively. In this current analysis, we fixed $\mu_{smp}$ and $\mu_{smn}$ at these posterior means of 0.196 and 0.049, respectively. These rates relate to HIV-negative individuals aged 55. The effects of HIV, ART, and age on TB mortality are explained in section 7.3 (due to the lack of data on the effect of these covariates in untreated TB, we make the same assumption about these factors for treated and untreated TB mortality).

## Modelling the tuberculosis diagnostic pathway

The path to TB diagnosis and care involves multiple steps; in the model, we consider the following:

- health-seeking rates, which vary by symptoms, smear status, HIV status and sex
- the proportion of sputum samples submitted for microbiological testing
- sensitivity and specificity of diagnostic algorithms
- treatment initiation and initial loss to follow-up (before treatment initiation) following a microbiological confirmation
- empirical treatment.

Treatment outcomes are further described in section 7.

Figure 3 summarises these steps above. At the entry point into the care cascade and treatment pathway, we consider health-seeking behaviours in people with active TB disease as well as health seeking unrelated to TB in the general population. We define $\gamma_{m}\left( a, g,s,t \right)$ as the rate of health-seeking for individuals of smear status $a$ (0 = smear-negative, 1 = smear-positive), of sex $g$ (0=male, 1=female) and HIV stage $s$ (as defined in Thembisa 4.1) at any time $t$. Here $m=1$ represents active TB individuals seeking treatment for TB symptoms, $m=2$ represents individuals in the general population who are seeking treatment for other conditions and $m=3$ represents individuals in the general population seeking treatment for TB- like symptoms. The health-seeking rates are defined such that for any $t$, $\gamma_{1}\left( a,g,s,t \right)$, $\gamma_{2}\left( g,s,t \right)$ and $\gamma_{3}\left( g,s,t \right)$ depend on smear status, sex, and HIV status.

Figure 3: Illustration of the modelled tuberculosis diagnostic pathway


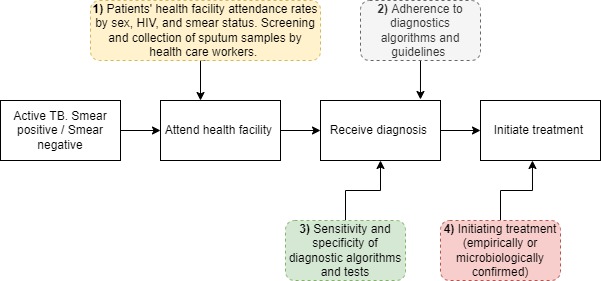


We also assume some prevalence ($v_{A}(a)$) of TB symptoms in the active TB population seeking care for other conditions, for smear status $a$ and we assume $v_{G}$ to be the prevalence of respiratory symptoms in the general (non-active TB) population. Following interaction with health facility workers, some individuals will be requested to submit their sputum samples for testing. Thus, we let $p_{1}\left( t \right)$ represent the proportion of individuals with active TB disease, seeking treatment for their TB symptoms, who submit their samples to be tested microbiologically; and $p_{2}\left( t \right)$ the proportion of individuals with TB-like respiratory symptoms, seeking treatment for other conditions, who get microbiologically tested.

We allow the diagnosis of TB to depend on the sensitivity ($Se\left( a,s,t \right)$) and specificity ($Sp\left( t \right)$) of the diagnostic algorithm for smear status $a$ and HIV state $s$, at time $t$. We consider the sensitivity and specificity of the first line microbiological diagnostic tests – GeneXpert MTB/RIF and smear microscopy – as well as culture (assumed to be 100%). The diagnostic algorithm is given by:

Equation 1: Diagnostic algorithm sensitivity

$$Se\left( a,t,s \right)=M\left( t \right)\left[ Se_{M}\left( a \right)+\left( 1-Se_{M}\left( a \right) \right)C_{M}\left( s,t \right) \right]$$

$$+ \left( 1-M\left( t \right) \right)\left[ Se_{G}\left( a \right) +\left( 1-Se_{G}\left( a \right) \right)C_{G}\left( a,s,t \right) \right]$$

And

Equation 2: Diagnostic algorithm specificity

$Sp\left( t \right)=M\left( t \right)Sp_{M}+\left( 1-M\left( t \right) \right)Sp_{G}$

where $Se_{M}\left( a \right)$ and $Se_{G}\left( a \right)$ represent the sensitivity of smear microscopy and GeneXpert MTB/RIF in active TB patients, respectively; $Sp_{M}$ and $Sp_{G}$ represent the specificity of smear microscopy and GeneXpert MTB/RIF, respectively. Given the recommendations that negative test results after an initial test require follow-up testing (particularly in the case of previously treated and HIV-positive cases (40,41)), we allow individuals to be followed up for a second test.

To represent the national-level implementation of the specific diagnostic tools, we define $M\left( t \right)$ to be the proportion of TB suspects who are initially tested by smear microscopy in year t and the remainder ($1-M\left( t \right)$) are tested by GeneXpert MTB/RIF initially. We then define $C_{G}\left( a,s,t \right)$ to represent the proportion of active TB cases followed-up for further culture testing after an initial negative GeneXpert MTB/RIF test; $C_{M}\left( s,t \right)$ is the proportion of active smear-negative TB cases who have further culture testing done after initial negative microscopy tests.

The estimated number of microbiological tests performed yearly $T\left( t \right)$ is given by

Equation 3: Number of microbiological tests

$T\left( t \right)$= $\sum_{g} \sum_{s} N\left( g,s,t \right)\left( \gamma_{2}\left( g,s,t \right)v_{G}p_{2}\left( t \right)+\gamma_{3}\left( g,s,t \right)p_{1}\left( t \right) \right)+ \sum_{a} \sum_{g} \sum_{s} A(a,g,s,t)\left( \gamma_{2}\left( g,s,t \right)v_{A}\left( a \right)p_{2}\left( t \right)+\gamma_{1}\left( a,g,s,t \right)p_{1}\left( t \right) \right)$

where $N\left( g,s,t \right)$ is the number of adults in the general population (not having active TB) and $A(a,g,s,t)$ is the number of active TB cases at time $t$.

Following a positive diagnosis, some patients get lost before treatment initiation. Therefore, we let $L(t)$ be the proportion of individuals lost to initial follow-up. The number of people with a positive laboratory diagnosis is represented by $R_{1}\left( t \right)$ in Equation 4 and the number of people with a positive diagnosis who initiate treatment is estimated as $R_{1}\left( t \right)(1-L(t))$.

Equation 4: Number of positive diagnoses

$R_{1}\left( t \right)=$

$\sum_{g} \sum_{s} N\left( g,s,t \right)\left( \gamma_{2}\left( g,s,t \right)v_{G}p_{2}\left( t \right)+\gamma_{3}\left( g,s,t \right)p_{1}\left( t \right) \right)\left( 1-Sp\left( t \right) \right)+ \sum_{a} \sum_{g} \sum_{s} A\left( a,g,s,t \right)\left( \gamma_{2}\left( g,s,t \right)v_{A}\left( a \right)p_{2}\left( t \right)+\gamma_{1}\left( a,g,s,t \right)p_{1}\left( t \right) \right)Se\left( a,t,s \right)$

### Modelling tuberculosis health-seeking patterns

#### The prevalence of tuberculosis symptoms by smear status

We set the prevalence of TB-related respiratory symptoms based on studies that report TB symptoms for the active or non-active TB populations (where the studied populations are at least generalisable). Based on the review by Onozaki *et al*. (42) of TB prevalence surveys in Asian populations, the average prevalence of TB symptoms in bacteriologically confirmed TB cases was 43% (42). A positive symptom screen was defined as a cough of more than two to three weeks or blood in sputum. A Zambia/South Africa TB and AIDS Reduction (ZAMSTAR) study also surveyed respiratory symptoms (43). Among individuals with TB, the proportion of people experiencing persistent cough for more than two weeks was 20.5%, compared to 5.2% among those without TB (43). Den Boon *et al.* in a Cape Town based study conducted in 2002, estimated the prevalence of TB symptoms to be 48% in patients with active TB (60% among smear-positive active TB patients and 22% among smear-negative individuals) (44).

We consider the prevalence observed in the ZAMSTAR (43) and the Den Boon *et al.* (44) studies to be likely over-estimates of the prevalence of symptoms in smear-negative TB ($v_{A}(0)$) because smear-negative TB is less symptomatic than smear-positive TB. To represent the uncertainty around this parameter we previously specified a Beta (3;12) prior with mean of 0.2 and standard deviation of 0.1. In this current analysis, we fixed this parameter at 0.198, which was estimated from a previous calibration analysis (mean = 0.198, 95% CI 0.149 – 0.263). Then we set $v_{G}$ the prevalence of respiratory symptoms in the general non-active TB population at 5.2% based on the ZAMSTAR study (43).

We use the review of prevalence surveys conducted in Asia between 1990 – 2012 by Onozaki *et al*., to estimate the ratio of the prevalence of TB symptoms in smear-positive individuals compared to smear-negative individuals with TB (42). From this review, the prevalence ratio of TB symptoms in smear-positive individuals compared to smear-negative individuals differed between countries and ranged between 1.0 and 2.72; the average was 1.62 (42). From the countries in which more than one survey was included (Cambodia, Philippines, Korea), the ratio declined over time, suggesting that the ratios might be lower as screening and treatment services improve. In the Den Boon *et al.* study, this ratio was at 2.7 (42,44)*.*

In the model, we define $f$ to be the ratio of symptoms in smear-positive compared to in smear-negative individuals. To represent the uncertainty around $f$ we previously specified a Gamma (19.36; 8.8) prior with mean 2.2 and standard deviation of 0.5. In this analysis, we fixed $f$ at 3.03, which was estimated in a previous calibration analysis (mean = 3.03, 95% CI 2.74 – 3.23).

#### Health-seeking patterns by sex

To model the effect of sex, we define $z$ to be the relative rate of health-seeking in females compared to males. We use the male-to-female ratio for prevalence-to-notification ratio estimated in the systematic review and meta-analysis by Horton *et al.* (19) to estimate sex differences on the pathway to TB care and TB testing. This ratio was estimated at 1.55 (19). To represent the uncertainty around this parameter $z$, we assign a Gamma (83.13; 53.63) prior distribution with a mean of $1.55$ and a standard deviation of 0.17.

#### Health-seeking patterns by HIV status

We assume that the attendance rate for HIV-infected individuals will be higher compared to that of HIV-uninfected individuals as HIV-infected individuals are more likely to be engaged with health care facilities and possibly because, in individuals with active TB, the development of symptoms may be more rapid than in HIV-negative individuals (45). We relied on Corbett *et al* and the South African National prevalence survey which suggested higher health-seeking in HIV-positive individuals than HIV-negative individuals (45,46).

We define $h$ to be the relative rate of health seeking in HIV-infected individuals (compared to HIV-uninfected individuals). We expect *h>*1. To represent the uncertainty around this parameter *h*, assign a Gamma (9;3) prior distribution with a mean of 3.0 and a standard deviation of 1 (45,46).

#### Setting overall health-seeking rates

For any time $t$, we define the health-seeking rates such that they depend on the variables:

- sex ($g$): male sex is represented by $g=0$; female $g=1$ $.$
- HIV status ($s$): HIV-uninfected status is represented by $s= 0$; HIV-infected by $s>0$.
- smear status ($a$): smear-negative TB represented by $a =0$; smear-positive by $a=1$.

The definitions of the health-seeking rates are below (***i.*** *–* ***iii.***), and as defined earlier:

- $f$ is the ratio of symptoms in smear-positive compared to in smear-negative individuals;
- $z$ is the relative rate of health-seeking in females compared to males;
- $h$ is the relative rate of health seeking in HIV-infected individuals (compared to HIV-uninfected individuals).

##### Health-seeking rate for active TB population seeking treatment for TB symptoms ($\boldsymbol{\gamma}_{\boldsymbol{1}}\left( \boldsymbol{a,g,s,t} \right)$)

We let $\gamma_{1}$ represent the rate of health-seeking for HIV-negative men with smear-negative active TB, seeking treatment for TB symptoms, and define $\gamma_{1}\left( a, g,s,t \right)$, the health-seeking rate for adults with active TB as below.

For smear-negative individuals ($a=0$),

$$\gamma_{1}\left( a, g,s,t \right)=\left\{ \begin{matrix} \gamma_{1} \text{for }g=0 \text{and} s=0, \forall t \\ \gamma_{1}z \text{for }g=1 \text{and} s=0, \forall t \\ \begin{matrix} \gamma_{1}h \text{for }g=0 \text{and} s>0, \forall t \\ \gamma_{1}zh \text{for }g=1 \text{and} s>0, \forall t \end{matrix} \end{matrix} \right.$$

For smear-positive individuals ($a=1$),

$$\gamma_{1}\left( a,g,s,t \right)=\left\{ \begin{matrix} \gamma_{1}f \text{for }g=0 \text{and} s=0, \forall t \\ \gamma_{1}zf \text{for }g=1 \text{and} s=0, \forall t \\ \begin{matrix} \gamma_{1}hf \text{for }g=0 \text{and} s>0, \forall t \\ \gamma_{1}zhf \text{for }g=1 \text{and} s>0, \forall t \end{matrix} \end{matrix} \right.$$

Based on the interquartile range of 1.3–3.4 prevalent infections per notification in men, from the review by Horton *et al.* (19), we have annual notification rates of between 0.29 (= 1/3.4) and 0.77 (= 1/1.3). Because many TB cases who seek treatment do not get screened, we estimate the rate of health-seeking by dividing the notification rate by the % screening, which ranges between 3% and 49% in South African studies (47–49). By taking the median of the South African studies estimates as 24%, we get the resulting range in the rates of health-seeking: 1.21 (=0.29/0.24) to 3.08 (=0.77/0.24). We specify a Gamma (19,07;8.91), prior distribution with mean = 2.14 and standard deviation = 0.49 per annum to represent the uncertainty around $\gamma_{1}$.

##### Health-seeking rate for the general population seeking treatment for other conditions ($\boldsymbol{\gamma}_{\boldsymbol{2}}\left( \boldsymbol{g,s,t} \right)$)

Second, we define the health-seeking rate for adults in the general population as

$$\gamma_{2}\left( g,s,t \right)=\left\{ \begin{matrix} \gamma_{2} \text{for }g=0 \text{and} s=0, \forall t \\ \gamma_{2}z \text{for }g=1 \text{and} s=0, \forall t \\ \begin{matrix} \gamma_{2}h \text{for }g=0 \text{and} s>0, \forall t \\ \gamma_{2}zh \text{for }g=1 \text{and} s>0, \forall t \end{matrix} \end{matrix} \right.$$

where $\gamma_{2}$ represents the rate of health seeking in HIV-negative men in the general population. We estimate this based on the average public health facilities attendance from the SADHS (21,50). This represents individuals who attend health facilities for other health conditions. The average attendance rates from the 1998 and 2003 SADHS reports were 0.186 and 0.2 per month, respectively. We take the average of these and get 0.193 per month, which gives an annual rate of 2.3 (= 0.193 $\times$ 12). We set this as an upper bound so $\gamma_{2}< 2.3$ because of telescoping bias – when surveyed, people tend to report things as happening more recently than they actually happened. Also, we would expect this to be an upper bound because the base rate ($\gamma_{2}$) applies to men who are HIV-negative, and we expect HIV-negative men to have lower rates of health seeking (generally) than women and people living with HIV. We specify a Gamma (5.29;4.6) prior distribution with mean = 1.15 and standard deviation = 0.5 to represent the uncertainty in $\gamma_{2}$.

##### Health-seeking rate for the general population (no TB) seeking treatment for TB-like symptoms $\boldsymbol{\gamma}_{\boldsymbol{3}}\left( \boldsymbol{g,s,t} \right)$

Third, we define the health-seeking rate for the general population without TB, seeking treatment for TB-like symptoms as

$$\gamma_{3}\left( g,s,t \right)=\left\{ \begin{matrix} \gamma_{3} \text{for }g=0 \text{and} s=0, \forall t \\ \gamma_{3}z \text{for }g=1 \text{and} s=0, \forall t \\ \begin{matrix} \gamma_{3}h \text{for }g=0 \text{and} s>0, \forall t \\ \gamma_{3}zh \text{for }g=1 \text{and} s>0, \forall t \end{matrix} \end{matrix} \right.$$

where $\gamma_{3}$ represents the rate at which HIV-negative men in the general population seek treatment for TB-like symptoms that are not due to TB. There are limited studies that show the proportions of individuals from the general population who attend health facilities due to TB-like symptoms only. We expect $\gamma_{2}>\gamma_{3}$– that is, the rate of health facility attendance ($\gamma_{2})$ will be greater than that for TB-like symptoms ($\gamma_{3}).$ Based on the South African General Household Survey in 2011 (51) on use of health facilities, 9.6% of South Africans reported being ill or injured in the previous month, and 77.5% of these reported consulting a health worker. This suggests an annual health-seeking rate of approximately 0.89 (= 0.096 $\times$ 0.775 $\times$ 12) (51). Of the people who were ill or injured in the month before the survey, 63.7% reported having flu or acute respiratory tract infections; and 2.9% reported having suffered from TB or severe cough with blood. From this, we approximate a lower bound of 0.0258 (= 0.029 $\times$ 0.89) and an upper bound of 0.593 (= (0.637 + 0.029) $\times$ 0.89), recognising that there is substantial overlap between TB symptoms and flu/acute respiratory symptoms (although it is unlikely that all flu/respiratory symptoms would be attributable to TB). We specify a Gamma (2.15;9.78) prior distribution with mean = 0.22 and standard deviation = 0.15 to represent the certainty around $\gamma_{3}.$

### Specimens submitted for microbiological testing

To estimate the proportions of individuals seeking treatment who are screened and have their sputum samples collected by health care workers for microbiological testing, we use estimates of the proportions reported in the few available empirical studies from South Africa (47–49). We allow these proportions to depend on patients’ reasons for attending the health facility, i.e., TB symptoms or other health conditions reported by patients (Table 6). Although all these studies rely heavily on patients' recall and may lead to biased assumptions, they give an idea of what happens at the health facility level. The Kweza *et al*. study was conducted across Eastern Cape district facilities, whereas the Claassens *et al*. study was limited to two primary health care facilities in one sub-district in the Western Cape. The Claassens *et al.* study also had a low response rate, suggesting it is less generalisable (48). The Chihota *et al.* study was a pragmatic cluster randomised trial assessing whether health care worker practice in examining people with TB symptoms changed when the initial test for TB switched from smear microscopy to GeneXpert MTB/RIF, so the results were presented separately for the two trial arms.

Table 6: Proportions of patients with TB symptoms who are screened and submit sputum specimens

|  |  | **Reason for attending a health facility** | |  | |  |
| --- | --- | --- | --- | --- | --- | --- |
| Study | | **TB symptoms** $\boldsymbol{p}_{\mathbf{1}}\mathbf{(}\boldsymbol{t}\mathbf{)}$ | **Other reasons** $\boldsymbol{p}_{\mathbf{2}}\mathbf{(}\boldsymbol{t}\mathbf{)}$ | | **Ratio** $\boldsymbol{r}\mathbf{=}\boldsymbol{p}_{\mathbf{1}}\mathbf{(}\boldsymbol{t}\mathbf{)/}\boldsymbol{p}_{\mathbf{2}}\mathbf{(}\boldsymbol{t}\mathbf{)}$ | |
| Claassens et al. (2013) (48) | | 0.028 | 0.003 | | 10.750 | |
| Chihota et al. (2015) (49)  GeneXpert MTB/RIF | | 0.491 | 0.154 | | 3.94 | |
| Microscopy | | 0.299 | 0.136 | | 2.195 | |
| Kweza et al. (2018) (47) | | 0.181 | 0.037 | | 4.893 | |

In these three studies (47–49), individuals were classified as attending health facilities due to TB-related (respiratory) symptoms if they reported having any symptoms, including cough, loss of weight, fever, and night sweats. This is a less specific definition than we considered in defining symptomatic TB for the prevalence of TB symptoms ($v_{A}, v_{G}$). As such, we note that the TB symptoms prevalence we use is an under-estimate of the true proportion of cases that could be screened.

We defined $r(t)= p_{1}(t)/p_{2}(t)$ to be the ratio of microbiological testing in symptomatic individuals seeking treatment for TB symptoms compared to those seeking treatment for other reasons (who coincidentally have TB-like symptoms) in year t. We let $p_{1}\left( t \right)$ represent the proportion of individuals with active TB seeking treatment for their TB symptoms who get tested microbiologically, and $p_{2}\left( t \right)$ the proportion of individuals with TB-like respiratory symptoms seeking treatment for other conditions who get microbiologically tested*.*

There is limited data and evidence to show how $r(t)$ has changed over time. However, the $r(t)$ estimates in Table 6 are highest when screening rates are lowest, suggesting that $r(t)$ has declined as the intensity of screening has increased. We used a piecewise-linear function to represent the change in $r(t)$ over time. First, we assume a constant ratio up to 2005. For this period, the ratio $r(t)$ was estimated by a fitting function ($\exp\left( 2.04 -10.84\times p_{2}(t) \right)+1$) for the relationship between the ratio and the screening rates in Table 6, which gives 8.71 when $p_{2}(t)$ = 0. We assigned a Gamma (12.14; 1.394) prior distribution with a mean of 8.71 and a standard deviation of 2.5 for $r(t)$ in the period up to 2005 (i.e., assuming screening rates were close to zero in the period before 2005). We then linearly interpolated between the 2006 and 2011 ratios for the intervening years; and assigned $r(t)$ a Gamma (11.11; 2.78) prior distribution with a mean of 4 and a standard deviation of 1.2 for the period from 2012. The latter prior distribution is based on the studies summarized in Table 6, which were mostly conducted in the period after 2011.

Then $p_{1}\left( t \right)$ will be estimated from $R_{1}\left( t \right)$, the total number of positive tests in year $t$, by rearranging the terms in Equation 4. We relied on the National Institute for Communicable Diseases data (2004-2019) for the numbers of microbiological diagnoses (52). Then, $p_{1}\left( t \right)$ was estimated as in Equation 5. We relied on the National Institute for Communicable Diseases data (2004-2019) for the numbers of microbiological diagnoses shown in Table 7 (52). We assumed the testing rates in 1985 (when the model was initiated) were half of the 2004 testing rates; and assumed that for the period 1985-2004, the screening rates increased linearly.

Equation 5: proportion of individuals with active TB who seek treatment for their TB symptoms who get tested microbiologically

$$p_{1}\left( t \right)= R_{1}\left( t \right)\left( \sum_{g} \sum_{s} N\left( g,s,t \right)\left( \gamma_{2}\left( g,s,t \right)v_{G}r\left( t \right)^{-1}+\gamma_{3}\left( g,s,t \right) \right)\left( 1-Sp\left( t \right) \right)+ \sum_{a} \sum_{g} \sum_{s} A\left( a,g,s,t \right)\left( \gamma_{2}\left( g,s,t \right)v_{A}\left( a \right)r\left( t \right)^{-1}+\gamma_{1}\left( a,g,s,t \right) \right)Se\left( a,t,s \right) \right)^{-1}$$

Table 7: Recorded numbers of laboratory confirmed tuberculosis cases by year

| **Year** | **Estimated number of microbiologically confirmed TB cases** |
| --- | --- |
| 2004 | 307385 |
| 2005 | 345694 |
| 2006 | 385496 |
| 2007 | 395907 |
| 2008 | 422134 |
| 2009 | 321558 |
| 2010 | 320125 |
| 2011 | 348400 |
| 2012 | 321206 |
| 2013 | 313013 |
| 2014 | 298389 |
| 2015 | 289136 |
| 2016 | 262454 |
| 2017 | 249090 |
| 2018 | 261743 |
| 2019 | 232483 |

Sources: Data for years 2004-2008 obtained from Nanoo et al (52) and adjusted for 10% probable overcounting. Data for years 2009-2019 obtained from the National Institute for Communicable Diseases (NICD) dashboard: <https://mstrweb.nicd.ac.za/MicroStrategy/asp/Main.aspx?Server=NICDSANDMSTRI01&Project=Surveillance&Port=0&evt=2048001&src=Main.aspx.2048001&documentID=4236FF364F683E2F257DA5AC647F5BA6&currentViewMedia=1&visMode>

### Sensitivity and specificity of diagnostic algorithms

In the model, we assumed sputum smear microscopy was the first-line diagnostic test before 2010. From 2011, we assume a gradual phase-in of GeneXpert MTB/RIF as the recommended first-line test (53). We let $M\left( t \right)$ be the proportion of individuals tested microbiologically by smear microscopy in year t. The remainder ($1-M\left( t \right)$) is the fraction who have an initial test by GeneXpert MTB/RIF. Thus $M\left( t \right)$ varies with time depending on GeneXpert MTB/RIF utilisation as the first-line tool for diagnosing TB in South Africa. For 2010 and years before, we assume the level of GeneXpert MTB/RIF utilisation was 0% and gradually phased in over time. These proportions were set to be the same as those assumed by Sumner *et al*. and Hippner *et al*. based on expert opinion (54,55) (Table 8).

Table 8: Utilisation of GeneXpert MTB/RIF as a first-line diagnostic test in South Africa

| Year | % GeneXpert MTB/RIF used ($\boldsymbol{1}-\boldsymbol{M}\left( \boldsymbol{t} \right))$ |
| --- | --- |
| 2010 and before | 0 |
| 2011 | 22 |
| 2012 | 43 |
| 2013 | 65 |
| 2014 | 73 |
| 2015 | 80 |
| 2016 and onwards | 80 |

Figure 4: Simplified diagnostic algorithm considered in the model


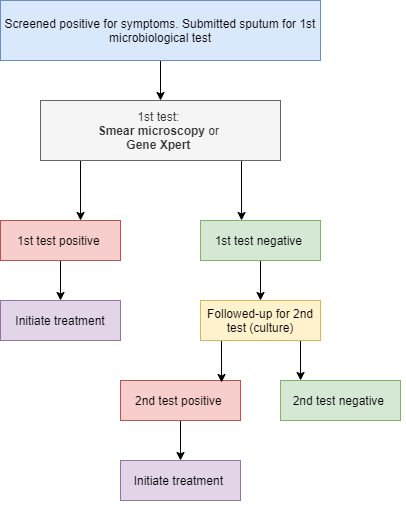

Note: second tests by culture mostly apply to HIV-positive individuals who are suspected to have TB disease and those with a history of TB treatment, but who test negative at the initial test.

As per the national TB guidelines (2008, 2009 and 2014), following a negative test result, it is recommended that some patients (i.e., HIV-positive individuals who are suspected to have TB disease and those with a history of TB treatment) be followed up for second tests by culture (40,53,56). In the model, we let the proportion of active TB cases of any smear status $a$, who have further culture tests performed after an initial negative GeneXpert MTB/RIF test, be represented by $C_{G}\left( a,s,t \right)$. $C_{M}\left( s,t \right)$ represents the proportion of smear-negative active TB cases with further culture testing after an initial negative smear microscopy test. These proportions of individuals followed up for second tests are based on studies which have evaluated follow-up tests by culture (Table 9) (57–59).

Table 9: Assumptions on follow-up tests by culture

| **Initial test** | **HIV status** | **Data sources description** | **Parameter value** |
| --- | --- | --- | --- |
| Smear negative | HIV-negative | Based on the ~30% fraction of retreatment cases from various studies conducted in the Western Cape, and a roughly 80% rate of culture testing in retreatment cases who are smear-negative and HIV-negative (58,59). | $C_{M}\left( s=0,t \right)$ = 0.24 |
|  | HIV-positive | Based on the average of the low rate (28%) in the McCarthy *et al* study (58) and the high rate (80%) in the Naidoo *et al* study (59). | $C_{M}\left( s>0,t \right):$ Before 2006: = 0.24.  In 2006: = 0.4.  After 2007: = 0.55. |
| GeneXpert MTB/RIF negative | HIV-negative | Based on the ~30% fraction of retreatment cases and ~50% rate of culture testing in retreatment cases who are smear-negative and HIV-negative (59). | $C_{G}\left( a,s=0,t \right)$ = 0.15 |
|  | HIV-positive | The average of the low rate (11%) in the McCarthy *et al* (58) study and the high rate (50%) in the Naidoo *et al* study (59). | $C_{G}\left( a,s>0,t \right)$ = 0.3 |

We further let a TB diagnosis depend on the sensitivity ($Se\left( a,t,s \right)$) and specificity ($Sp\left( t \right)$) of the diagnostic algorithm for smear status $a$, at time $t$ and HIV status $s$ (as defined in equations 1 and 2). $Se_{M}\left( a \right)$ and $Se_{G}\left( a \right)$ represent the sensitivity of smear microscopy and GeneXpert MTB/RIF in active TB patients, respectively; $Sp_{M}$ and $Sp_{G}$ represent the specificity of smear microscopy and GeneXpert MTB/RIF, respectively. The culture test is assumed to be 100% sensitive and 100% specific. The assumed values for the other tests are shown in Table 10 (60–63).

Table 10: Assumed sensitivity and specificity of diagnostic tests by smear-status

|  | **Value** | **Source** |
| --- | --- | --- |
| Sensitivity of GeneXpert MTB/RIF (${Se}_{G}\left( a \right)$) |  |  |
| Smear-positive (${Se}_{G}\left( 1 \right)$) | 0.98 | Horne 2019 (63) |
| Smear-negative (${Se}_{G}\left( 0 \right)$) | 0.67 | Horne 2019 (63) |
| Sensitivity of smear microscopy (${Se}_{M}\left( a \right)$) |  |  |
| Smear-positive (${Se}_{M}\left( 1 \right)$) | 1 | Assumed |
| Smear-negative (${Se}_{M}\left( 0 \right)$) | 0 | Assumed |
| Specificity of tests |  |  |
| GeneXpert MTB/RIF $(Sp_{G})$ | 0.995 | Horne 2019; Parker 2019 (63,64) |
| Microscopy $(Sp_{M})$ | 0.98 | Steingart 2006 (65) |

### Treatment initiation and initial loss to follow-up

Following a positive diagnosis, some individuals are lost to follow-up before initiating treatment – this is referred to as initial loss to follow-up (ILTFU). The definition of ILTFU depends on the follow-up period at which individuals would be regarded as lost to follow-up. In most studies (66,67), individuals are classified as lost to initial follow-up if they do not initiate treatment one to six months after receiving a positive diagnosis and do not get recorded in the TB treatment registers maintained at health facilities.

A systematic review of 16 South African studies reported a pooled estimate of 19.4% (95% CI 14.4 – 24.3%) of ILTFU (66). However, there was high variation among the studies included in this review (I^2^ = 95.85%). Factors associated with initial loss to follow-up include diagnostic tools – the longer the turnaround to test results, the more likely it is to lose patients on the treatment pathway. Because GeneXpert MTB/RIF is a point of care test with a quicker turnaround, a lower ILTFU is generally observed with its use. The average proportion of initial loss to follow-up in studies where GeneXpert MTB/RIF was used was 14.2%. In study settings where smear microscopy was used, the average proportion of initial loss to follow-up was 21.6% (66).

In another earlier review, Macpherson *et al.* (2014) estimated an initial loss to follow up of 18.0% (95% CI 13.0 – 22.0%) based on studies from African countries (67). Only one study in this review (Botha *et al.* 2008 (68)) traced patients who were initially lost to follow-up. In this study, 58 bacteriologically confirmed TB cases were initially classified LTFU. Upon follow-up of these individuals, 24.1% (n=14) had died, 44.8% (n=26) could not be traced and 31.0% (n=18) were traced (68). Of these 18 that were traced, 11 were found to have started treatment late, and seven were traced but had not started treatment for various reasons. This study showed that based on the definition used by various studies, particularly those with a short follow-up period, there is a chance of misclassifying a certain proportion of individuals who initiate treatment as lost to follow-up. That is, the proportion of initial loss to follow-up may be overestimated.

In the model, we set $L(t)$, the proportion of initial loss to follow-up in year t, based on the systematic review of South African studies, adjusted for the likely over-estimation identified by Botha *et al.* We adjust by the fraction of ILTFU cases that eventually get back into the health system and on treatment. Based on the Botha *et al.* study still, and assuming that those who were not traced were as likely to be on treatment as those who were traced (non-differential with respect to initiating treatment), we can assume the actual initial loss to follow-up is (14 + 7) / (58 – 26) = 66%. We apply this factor of 0.66 to the assumed initial loss to follow-up. So, when Xpert is used, $L\left( t \right)=0.142\times0.66= 0.094$ and when smear microscopy was used, $L\left( t \right)=0.216\times0.66=0.143$*.* The number of individuals who start treatment after a diagnosis is a shown in Equation 4.

### Modelling empirical treatment

Empirical TB treatment – the administration of TB treatment to individuals being assessed for TB disease who do not have laboratory confirmation of TB – is recommended by WHO guidelines in resource-limited settings when ambulant HIV- radiography findings compatible with TB do not respond to broad-spectrum antimicrobial therapy (69). In South Africa, clinicians are recommended to initiate HIV-positive individuals on TB treatment when two sputum smear microscopy tests or a single sputum GeneXpert MTB/RIF is negative for TB, chest radiograph findings are compatible with TB, and symptoms do not respond to antibiotics (56). Empirical treatment is more beneficial in situations where the probability of active TB before a test is high, and when the diagnostic test used is less sensitive (56). As such, in some situations, even when the guidelines recommend it, clinicians initiate patients on TB treatment on an empirical basis.

In our model, we account for individuals who initiated treatment on an empirical basis through the calibration process among the people who initiated treatment. In modelling empirical treatment, we make an implicit allowance for extrapulmonary TB (as it is difficult to diagnose and the majority of extrapulmonary TB cases are not diagnosed based on microbiological investigation (70)).

First, we define $Y$ as the proportion of active TB cases seeking treatment for TB symptoms who get treated empirically before any microbiological test is conducted. This proportion is based on two studies. The first study is by Pepper *et al*., which found the proportion to be 13% in Khayelitsha (based on a large sample in 2007-9 before GeneXpert MTB/RIF was introduced) (71). The second is by Pronyk *et al*., estimating a much higher rate (38%) in Agincourt, based on a relatively small number of cases (72). However, these studies may overestimate the true proportion. For example, if TB cases that come to the clinic with symptoms but never get screened are included in the denominator, the proportion will be lower. This bias would be more likely when there are lower treatment rates (i.e., as in the Pronyk *et al.* study conducted in a rural area before the rollout of major TB programmes)[[1]](#footnote-2). Therefore, the average of these two studies (25%) is assumed as the upper bound. We then specify a Uniform (0;0.25) prior distribution for Y, with a mean of 0.125 and standard deviation of 0.144.

Second, we let $Z$ represent the proportion of smear-negative active TB cases seeking treatment for TB symptoms, treated empirically after an initial negative smear. In the period before GeneXpert MTB/RIF (i.e., before 2011), the proportion of all treated TB cases treated empirically was between 30% and 45% (73–75). It is nonetheless difficult to quantify the parameter based on the available data, and therefore we assign a non-informative prior, U(0;0.667) to represent the uncertainty in Z, with a mean of 0.33 and standard deviation of 0.236.

Thirdly, we let $R$ be the relative rate of empirical treatment in people with respiratory symptoms that are not due to TB, compared to people who do have TB. This $R$ parameter is difficult to estimate from published studies due to a lack of evidence. However, we expect it to be $<1$ since chest radiography is often used to exclude patients who do not have TB, and many empirically treated patients are only started on treatment if they fail to respond to an initial short course of broad-spectrum antibiotics. Based on van ‘t Hoog *et al*.'s meta-analysis, the sensitivity of chest X-rays in detecting TB was 95% based on the studies that evaluated chest X-rays in patients who had symptoms; the specificity was 55% (76). This suggests that if chest X-rays tested everyone with symptoms, the value of $R$ would be 0.47 (= (1 – 0.55)/0.95). However, this might be an overestimate because not everyone is examined with chest X-rays; therefore, we specified a non-informative prior U(0,1), (mean=0.5; standard deviation=0.236) to be conservative.

Fourthly, we define $H$ as the relative rate of empirical treatment in patients who have TB symptoms (but came to the clinic for other reasons) relative to patients who came to the clinic because of TB-like symptoms. This parameter is also difficult to estimate from the literature. We expect that people who came to the clinic for other reasons would, on average, have less severe disease than those who came because of TB-like symptoms. As such, we assume health workers would perhaps be more cautious about putting them on treatment without microbiological confirmation. Therefore, we would expect $H$ to be $<1$ and again we assigned a non-informative prior (Uniform (0, 1), (mean=0.5; standard deviation=0.236) to be conservative.

Lastly, we define $G$ to be the extent to which the introduction of GeneXpert MTB/RIF reduces empirical treatment after an initial negative test result. (We assume that the introduction of GeneXpert MTB/RIF does not cause any change in the rate of empirical treatment in people who have never been tested, i.e., it modifies Z but not Y). Hermans *et al*. showed that the proportion of patients empirically treated dropped from 32% in 2010 (before the introduction of GeneXpert MTB/RIF) to 18% in 2014 (75). Rees *et al*. observed a more modest reduction in empirical treatment from 37.5% in 2012 to 29.4% in 2015 (77). We thus set $G$, the reduction in empirical treatment after a negative screen due to GeneXpert MTB/RIF at 0.5 based on these studies.

These $Y, Z, R$ and H parameters will be estimated by calibrating the model to the number of active TB cases that are initiating treatment as recorded in the ETR and the numbers of microbiological diagnoses. Although the difference between the two gives a crude measure of the extent of empirical treatment, a limitation is that there is limited empirical data to calibrate the model.

## Modelling tuberculosis treatment and treatment outcomes

In South Africa, the standard TB treatment for drug susceptible TB lasts for six months, consisting of two phases. The first (intense) phase lasts two months, and the second (continuation) phase lasts four months (40). The commonly reported treatment outcomes include default, success (cure and completion), failure and death. The definitions are as in Table 11. These outcomes are reported as percentages in the District Health Barometer, and the primary data source is the ETR.

Table 11: Definitions of data used for treatment outcomes as per the electronic tuberculosis register

| **Symbol** | **Description** | **Source** |
| --- | --- | --- |
| $m$ | Treatment completion: Proportion of patients whose smear (or culture) was positive at the beginning of treatment and have completed treatment but does not have negative smear /culture in the last month of treatment and on at least one previous occasion more than 30 days before. The numerator is the number of patients who have completed treatment as per definition, and the denominator is the total number of TB patients on treatment that year (41). | Electronic tuberculosis register (40,41). |
| $c$ | Cured: Proportion of patients whose smear (or culture) was positive at treatment initiation and is smear/ culture-negative in the last month of treatment and on at least one previous occasion more than 30 days before. The numerator is the number of patients who are cured as per definition, and the denominator is the total number of TB patients on treatment that year (41). |
| $f$ | Treatment failure: Proportion of patients whose baseline smear (or culture) was positive and remained or becomes positive again at five months or later during treatment. This also includes patients with no significant clinical improvement and no significant weight gain after 4-5 months of treatment. A clinician establishes the diagnosis of the failure. The numerator is the number of patients who have failed treatment as defined, and the denominator is the total number of TB patients on treatment that year (41). |
| $d_{1}$ | Death: Proportion of patients who die for any reason during TB treatment. The numerator is the number of patients who die on treatment as defined, and the denominator is the total number of TB patients on treatment that year. |
| $d_{2}$ | Treatment discontinuation/default: Proportion of patient whose treatment was interrupted for two consecutive months or more during the treatment course. The numerator is the number of patients who have treatment interruption as per definition, and the denominator is the total number of TB patients on treatment that year (41). |

Note: definitions do not explicitly describe how people get classified if they start treatment without a positive microbiological test.

We model four possible outcomes: cure ($\kappa$) and treatment failure ($\gamma$) after treatment completion, treatment discontinuation ($\delta$) and death on treatment ($\mu$). These outcomes are estimated from the annual percentages reported by the ETR (Table 12). The ETR outcomes are defined differently from our model, as in the ETR treatment success is defined as a combination of cure and completion of the treatment course, whereas we consider 'cure after treatment completion’ and also allow for cure in some patients who discontinue treatment. Based on the six-month treatment duration, we convert the percentages (Table 12) from the ETR into annual rates that correspond to model parameters shown in Table 13.

Patients classified as cured in ETR (with bacteriological confirmation) would have completed the 6-months treatment course. For those classified as ‘completed treatment’, it is uncertain whether they have been cured. Using the ETR proportions, we estimate rates to represent transitions out of the 6-months treatment state. From the proportion of treatment success, we use the proportion of those cured ($c$) to estimate a cure rate ($\kappa$). This $\kappa$ represents a transition to the recovered state after 6-months of treatment. Following treatment failure ($\gamma$), individuals are assumed to move back to active TB states, assuming the same distribution of smear status as for new active TB cases.

Among those who experience treatment discontinuation, at annual rate $\delta$, we assume a proportion ($1-\phi)$ will move back to the active TB states and that $\phi$ move to the recently treated recovered state (post-treatment short term). Here $\phi$ represents the efficacy of partial/incomplete treatment, and we set this based on previous studies. Based on what was observed in a retrospective cohort study assessing treatment outcomes (78), among those who defaulted during TB treatment, ~35% developed TB disease within two years. Using these results, we assume that of those discontinue TB treatment, 65% would be cured and move to the recovered state (78).

Lastly, patients on treatment die at a rate of $\mu.$ One of the problems with TB deaths on treatment is under-reporting which is usually due to deaths being classified as treatment discontinuation (a loss to follow-up/default) (79). In the context of HIV and ART, under-ascertainment of deaths in HIV-infected individuals due to loss to follow-up has been corrected using factors ranging between 1.64 and 2.19 (80). In South Africa, it has been shown that only 35% (95% CI 34.2 - 35.8%) of ART patient deaths recorded in the vital registration system were also captured in patient records at health facilities (81), although higher rates of ascertainment might be expected in the case of TB given the DOTS requirements for regular patient contact. Given the similar challenges of under-reporting of TB deaths in patients on treatment, we use this observation to adjust for TB deaths. As such, we assume the true mortality rate is double the reported mortality rate. More details on how these parameters are set and adjusted follow in the next sub-sections below.

Table 12: Electronic Tuberculosis Register Treatment outcomes by year (2004 – 2016) sex and HIV-status expressed as proportions

|  |  | **HIV-infected** | | | | | **HIV-uninfected** | | | | | **HIV-unknown** | | | | |
| --- | --- | --- | --- | --- | --- | --- | --- | --- | --- | --- | --- | --- | --- | --- | --- | --- |
| **Sex** | **Year** | **Completed** | **Cured** | **Died** | **Discontinued** | **Failed** | **Completed** | **Cured** | **Died** | **Discontinued** | **Failed** | **Completed** | **Cured** | **Died** | **Discontinued** | **Failed** |
|  |  | $m$ | $c$ | $d_{1}$ | $d_{2}$ | $f$ | $m$ | $c$ | $d_{1}$ | $d_{2}$ | $f$ | $m$ | $c$ | $d_{1}$ | $d_{2}$ | $f$ |
| Female | 2004 | 0.468 | 0.245 | 0.060 | 0.220 | 0.008 | 0.396 | 0.445 | 0.022 | 0.128 | 0.009 | 0.452 | 0.202 | 0.085 | 0.256 | 0.006 |
|  | 2005 | 0.454 | 0.259 | 0.086 | 0.192 | 0.008 | 0.314 | 0.538 | 0.021 | 0.117 | 0.010 | 0.458 | 0.220 | 0.093 | 0.222 | 0.007 |
|  | 2006 | 0.455 | 0.266 | 0.092 | 0.176 | 0.010 | 0.325 | 0.522 | 0.025 | 0.117 | 0.011 | 0.475 | 0.225 | 0.098 | 0.196 | 0.007 |
|  | 2007 | 0.458 | 0.248 | 0.105 | 0.179 | 0.009 | 0.372 | 0.455 | 0.031 | 0.129 | 0.013 | 0.466 | 0.234 | 0.100 | 0.192 | 0.007 |
|  | 2008 | 0.465 | 0.237 | 0.116 | 0.174 | 0.009 | 0.433 | 0.380 | 0.041 | 0.137 | 0.009 | 0.477 | 0.221 | 0.098 | 0.197 | 0.007 |
|  | 2009 | 0.472 | 0.243 | 0.109 | 0.167 | 0.009 | 0.463 | 0.359 | 0.040 | 0.129 | 0.009 | 0.500 | 0.228 | 0.095 | 0.171 | 0.006 |
|  | 2010 | 0.472 | 0.235 | 0.100 | 0.185 | 0.008 | 0.465 | 0.338 | 0.040 | 0.149 | 0.008 | 0.477 | 0.227 | 0.093 | 0.195 | 0.007 |
|  | 2011 | 0.482 | 0.264 | 0.095 | 0.149 | 0.010 | 0.477 | 0.351 | 0.040 | 0.123 | 0.009 | 0.505 | 0.232 | 0.087 | 0.168 | 0.007 |
|  | 2012 | 0.479 | 0.275 | 0.095 | 0.140 | 0.011 | 0.485 | 0.351 | 0.043 | 0.113 | 0.008 | 0.499 | 0.248 | 0.080 | 0.167 | 0.007 |
|  | 2013 | 0.510 | 0.262 | 0.086 | 0.134 | 0.008 | 0.532 | 0.315 | 0.040 | 0.107 | 0.006 | 0.502 | 0.227 | 0.080 | 0.185 | 0.006 |
|  | 2014 | 0.594 | 0.153 | 0.073 | 0.170 | 0.009 | 0.603 | 0.210 | 0.038 | 0.143 | 0.006 | 0.522 | 0.117 | 0.083 | 0.264 | 0.015 |
|  | 2015 | 0.685 | 0.121 | 0.079 | 0.107 | 0.007 | 0.695 | 0.175 | 0.037 | 0.088 | 0.005 | 0.639 | 0.086 | 0.081 | 0.178 | 0.017 |
|  | 2016 | 0.641 | 0.161 | 0.078 | 0.114 | 0.006 | 0.630 | 0.237 | 0.037 | 0.090 | 0.006 | 0.627 | 0.114 | 0.073 | 0.180 | 0.006 |
| Male | 2004 | 0.415 | 0.238 | 0.074 | 0.258 | 0.014 | 0.342 | 0.487 | 0.019 | 0.134 | 0.019 | 0.410 | 0.218 | 0.079 | 0.285 | 0.008 |
|  | 2005 | 0.417 | 0.264 | 0.093 | 0.213 | 0.012 | 0.271 | 0.530 | 0.027 | 0.161 | 0.011 | 0.422 | 0.230 | 0.089 | 0.251 | 0.009 |
|  | 2006 | 0.390 | 0.285 | 0.102 | 0.214 | 0.010 | 0.275 | 0.500 | 0.032 | 0.178 | 0.015 | 0.435 | 0.236 | 0.095 | 0.225 | 0.009 |
|  | 2007 | 0.422 | 0.249 | 0.110 | 0.208 | 0.012 | 0.321 | 0.444 | 0.042 | 0.178 | 0.015 | 0.428 | 0.240 | 0.100 | 0.223 | 0.009 |
|  | 2008 | 0.429 | 0.238 | 0.124 | 0.200 | 0.010 | 0.381 | 0.384 | 0.051 | 0.172 | 0.012 | 0.433 | 0.232 | 0.100 | 0.227 | 0.008 |
|  | 2009 | 0.439 | 0.240 | 0.121 | 0.190 | 0.009 | 0.407 | 0.373 | 0.051 | 0.158 | 0.012 | 0.442 | 0.243 | 0.097 | 0.209 | 0.008 |
|  | 2010 | 0.436 | 0.236 | 0.111 | 0.208 | 0.009 | 0.408 | 0.352 | 0.051 | 0.179 | 0.011 | 0.420 | 0.250 | 0.094 | 0.227 | 0.008 |
|  | 2011 | 0.444 | 0.259 | 0.111 | 0.175 | 0.011 | 0.417 | 0.363 | 0.052 | 0.157 | 0.010 | 0.443 | 0.253 | 0.089 | 0.208 | 0.008 |
|  | 2012 | 0.447 | 0.275 | 0.108 | 0.160 | 0.011 | 0.422 | 0.370 | 0.054 | 0.145 | 0.010 | 0.425 | 0.282 | 0.087 | 0.198 | 0.008 |
|  | 2013 | 0.478 | 0.267 | 0.094 | 0.152 | 0.009 | 0.463 | 0.342 | 0.049 | 0.136 | 0.009 | 0.440 | 0.266 | 0.077 | 0.207 | 0.009 |
|  | 2014 | 0.562 | 0.161 | 0.082 | 0.185 | 0.010 | 0.547 | 0.230 | 0.047 | 0.167 | 0.009 | 0.480 | 0.149 | 0.077 | 0.278 | 0.015 |
|  | 2015 | 0.655 | 0.130 | 0.083 | 0.124 | 0.008 | 0.626 | 0.202 | 0.047 | 0.118 | 0.008 | 0.579 | 0.123 | 0.087 | 0.197 | 0.013 |
|  | 2016 | 0.611 | 0.176 | 0.080 | 0.125 | 0.008 | 0.566 | 0.263 | 0.046 | 0.117 | 0.008 | 0.569 | 0.158 | 0.069 | 0.198 | 0.007 |

Table 13: Electronic Tuberculosis Register Treatment outcomes by year (2004 – 2016), sex and HIV-status expressed as rates

|  |  | **HIV-infected** | | | |  | **HIV-uninfected** | | | |  | **HIV-unknown** | | | |
| --- | --- | --- | --- | --- | --- | --- | --- | --- | --- | --- | --- | --- | --- | --- | --- |
| **Sex** | **Year** | **Failed**  **($\boldsymbol{\gamma}$)** | **Cure ($\boldsymbol{\kappa}$)** | **Discontinuation ($\boldsymbol{\delta}$)** | **Death ($\boldsymbol{\mu}$)** |  | **Failed**  **($\boldsymbol{\gamma}$)** | **Cure ($\boldsymbol{\kappa}$)** | **Discontinuation ($\boldsymbol{\delta}$)** | **Death ($\boldsymbol{\mu}$)** |  | **Failed**  **($\boldsymbol{\gamma}$)** | **Cure ($\boldsymbol{\kappa}$)** | **Discontinuation ($\boldsymbol{\delta}$)** | **Death ($\boldsymbol{\mu}$)** |
|  |  |  |  |  |  |  |  |  |  |  |  |  |  |  |  |
| **Female** | 2004 | 0.066 | 1.934 | 0.444 | 0.331 |  | 0.039 | 1.961 | 0.249 | 0.104 |  | 0.054 | 1.946 | 0.518 | 0.513 |
|  | 2005 | 0.062 | 1.938 | 0.294 | 0.476 |  | 0.037 | 1.963 | 0.223 | 0.097 |  | 0.061 | 1.939 | 0.378 | 0.541 |
|  | 2006 | 0.075 | 1.925 | 0.228 | 0.505 |  | 0.043 | 1.957 | 0.215 | 0.116 |  | 0.059 | 1.941 | 0.278 | 0.552 |
|  | 2007 | 0.072 | 1.928 | 0.207 | 0.586 |  | 0.055 | 1.945 | 0.233 | 0.149 |  | 0.061 | 1.939 | 0.262 | 0.563 |
|  | 2008 | 0.070 | 1.930 | 0.164 | 0.651 |  | 0.046 | 1.954 | 0.235 | 0.199 |  | 0.057 | 1.943 | 0.279 | 0.558 |
|  | 2009 | 0.068 | 1.932 | 0.159 | 0.604 |  | 0.051 | 1.949 | 0.213 | 0.194 |  | 0.054 | 1.946 | 0.206 | 0.518 |
|  | 2010 | 0.069 | 1.931 | 0.237 | 0.562 |  | 0.046 | 1.954 | 0.269 | 0.198 |  | 0.058 | 1.942 | 0.287 | 0.523 |
|  | 2011 | 0.072 | 1.928 | 0.144 | 0.504 |  | 0.050 | 1.950 | 0.199 | 0.189 |  | 0.060 | 1.940 | 0.216 | 0.469 |
|  | 2012 | 0.074 | 1.926 | 0.116 | 0.499 |  | 0.043 | 1.957 | 0.167 | 0.203 |  | 0.052 | 1.948 | 0.231 | 0.424 |
|  | 2013 | 0.062 | 1.938 | 0.123 | 0.440 |  | 0.040 | 1.960 | 0.157 | 0.188 |  | 0.054 | 1.946 | 0.284 | 0.437 |
|  | 2014 | 0.107 | 1.893 | 0.256 | 0.389 |  | 0.059 | 1.941 | 0.256 | 0.184 |  | 0.224 | 1.776 | 0.553 | 0.508 |
|  | 2015 | 0.114 | 1.886 | 0.068 | 0.391 |  | 0.060 | 1.940 | 0.115 | 0.171 |  | 0.336 | 1.664 | 0.263 | 0.435 |
|  | 2016 | 0.070 | 1.930 | 0.090 | 0.386 |  | 0.047 | 1.953 | 0.121 | 0.170 |  | 0.096 | 1.904 | 0.287 | 0.392 |
| **Male** | 2004 | 0.112 | 1.888 | 0.549 | 0.445 |  | 0.074 | 1.926 | 0.271 | 0.088 |  | 0.073 | 1.927 | 0.647 | 0.498 |
|  | 2005 | 0.090 | 1.910 | 0.346 | 0.539 |  | 0.042 | 1.958 | 0.328 | 0.134 |  | 0.076 | 1.924 | 0.493 | 0.536 |
|  | 2006 | 0.065 | 1.935 | 0.330 | 0.594 |  | 0.057 | 1.943 | 0.371 | 0.162 |  | 0.070 | 1.930 | 0.383 | 0.559 |
|  | 2007 | 0.093 | 1.907 | 0.287 | 0.644 |  | 0.065 | 1.935 | 0.350 | 0.214 |  | 0.073 | 1.927 | 0.363 | 0.590 |
|  | 2008 | 0.080 | 1.920 | 0.225 | 0.731 |  | 0.062 | 1.938 | 0.310 | 0.263 |  | 0.069 | 1.931 | 0.378 | 0.594 |
|  | 2009 | 0.076 | 1.924 | 0.202 | 0.699 |  | 0.061 | 1.939 | 0.271 | 0.257 |  | 0.066 | 1.934 | 0.323 | 0.560 |
|  | 2010 | 0.074 | 1.926 | 0.286 | 0.650 |  | 0.058 | 1.942 | 0.334 | 0.263 |  | 0.059 | 1.941 | 0.392 | 0.557 |
|  | 2011 | 0.081 | 1.919 | 0.179 | 0.622 |  | 0.055 | 1.945 | 0.266 | 0.265 |  | 0.062 | 1.938 | 0.339 | 0.504 |
|  | 2012 | 0.076 | 1.924 | 0.142 | 0.587 |  | 0.053 | 1.947 | 0.228 | 0.268 |  | 0.056 | 1.944 | 0.311 | 0.486 |
|  | 2013 | 0.067 | 1.933 | 0.153 | 0.500 |  | 0.052 | 1.948 | 0.215 | 0.239 |  | 0.069 | 1.931 | 0.364 | 0.433 |
|  | 2014 | 0.118 | 1.882 | 0.281 | 0.449 |  | 0.071 | 1.929 | 0.306 | 0.240 |  | 0.185 | 1.815 | 0.625 | 0.480 |
|  | 2015 | 0.112 | 1.888 | 0.102 | 0.421 |  | 0.078 | 1.922 | 0.171 | 0.223 |  | 0.198 | 1.802 | 0.307 | 0.489 |
|  | 2016 | 0.083 | 1.917 | 0.114 | 0.400 |  | 0.059 | 1.941 | 0.169 | 0.219 |  | 0.081 | 1.919 | 0.351 | 0.374 |

Adjustment: Applied a factor of 2 to the ETR TB deaths and assume that unrecorded deaths are incorrectly classified as a loss to follow-up. We then subtracted from the proportion of those lost-to-follow-up for the fraction that gets added to deaths

### Modelling cure and failure

To estimate cure and failure rates, we assume that the ratio of failures to cures is the same in the treatment completers with missing outcomes as in those with recorded cure or failure outcomes.

We estimate the annual rate at which treatment is completed in cured patients as:

$$\kappa=\left( \frac{c}{c+f} \right)/0.5$$

and the rate at which treatment is completed in patients who are failing as:

$$\gamma=\left( \frac{f}{c+f} \right)/0.5$$

We thus have $\kappa+\gamma=2$ (implying a treatment duration of half a year among those who complete treatment) and assume that the ratio of failures to cures is the same in the treatment completers with missing outcomes as in the completers with recorded cure or failure outcomes. Then we define $d=d_{1}+d_{2}$ as the proportion of patients who have died or have discontinued their treatment. From $\mu$, the annual mortality rate on treatment, and $\delta$, the annual treatment discontinuation rate, we define the rate $D=\mu+\delta$. From the above, we have $d_{1}+d_{2}=\frac{\mu+\delta}{\mu+\delta+ \kappa+\gamma}$ so $d=\frac{D}{D+2}$*.* $D$ can then be expressed as $D=\frac{2d}{1-d}$. In the absence of any correction for under-ascertainment of mortality, we would have $\mu$ and $\delta$ expressed as $\mu=\frac{d_{1}D}{d}$ and $\delta=\left( 1-\frac{d_{1}}{d} \right)D$. If we assume the true mortality rate is double the reported mortality rate, we have $\mu=\frac{2d_{1}D}{d}$ and $\delta=\left( 1-\frac{2d_{1}}{d} \right)D$. We used the ETR data and set the cure rate at $\kappa=1.92$, roughly the average across all years (Table 13) for those who complete the six months treatment course.

### Modelling treatment discontinuation

In the model, we specify a fixed parameter for the annual rate of treatment discontinuation. Based on the ETR data, males have higher treatment discontinuation rates than females (Table 14). This treatment discontinuation parameter is calculated as the average discontinuation rates across years and HIV strata, and we set it at 0.309 in males and 0.237 for females. Using the ETR data, the ratio of male to female average discontinuation rate aggregated across age and HIV strata is 1.307 (= 0.309/0.237). Berry *et al.'s* study using the ETR data for Gauteng Province (Ekurhuleni Metropolitan Municipality and the City of Johannesburg), estimated a similar relative risk of treatment discontinuation for males compared to females, 1.299 (82).

Table 14: Average treatment discontinuation and death rates by HIV status, based on the ETR

|  | **HIV-positive** | | **HIV-negative** | | **HIV-unknown** | | **Overall** | |
| --- | --- | --- | --- | --- | --- | --- | --- | --- |
|  | **discontinuation** | **death** | **discontinuation** | **death** | **discontinuation** | **death** | **discontinuation** | **death** |
| Female | 0.195 | 0.486 | 0.204 | 0.166 | 0.311 | 0.495 | 0.236 | 0.382 |
| Male | 0.246 | 0.560 | 0.276 | 0.218 | 0.406 | 0.512 | 0.309 | 0.430 |
| Male: Female ratio | 1.263 | 1.151 | 1.354 | 1.311 | 1.305 | 1.035 | 1.308 | 1.166 |

### Modelling tuberculosis deaths on treatment

Based on the ETR data, we set the mortality rates on TB treatment to be at 0.192 (average for both males and females, Table 14), averaged across years in the HIV-negative strata. The subsequent sections describe the effects of disease severity, age, HIV and ART on TB mortality in treated individuals.

#### Modelling the effect of changes in tuberculosis disease severity on mortality

We allow for treated TB mortality rates to change over time, since changes in delays between disease incidence and treatment should imply changes in the average severity of treated TB cases. Due to limited empirical evidence, it is difficult to assess how smear grades have changed over time as levels of diagnosis and treatment have improved. In the model we define (as in the earlier section: 4.1) the average smear-positive treatment delay in year $t$, $S\left( t \right)$, as $U\left( t \right)/R(t)$, where $U\left( t \right)$ is the number of untreated smear-positive TB cases at the start of year *t* and $R(t)$ is the number of smear-positive TB patients who are treated in year $t$. As $S\left( t \right)$ declines towards zero, we assume a corresponding decline in mortality toward a theoretical minimum that might be expected if all smear-positive TB cases were graded scanty or 1+ at treatment initiation. To simplify, we assume that TB diagnosis and treatment levels in South Africa were low during the period before 2000 and that any improvements that occurred before 2000 were minor.

We suppose $\mu_{g}\left( t \right)$ represents the mortality rate in smear-positive HIV-negative treated TB patients of sex in year $t$, and that $\mu_{g}\left( 0 \right)$ represents the corresponding mortality rate in the period before 2000. We also suppose that $S\left( 0 \right)$ is the value of $S\left( t \right)$ in 1999, which we take to represent the period before 2000. We assume that for $t \geq2000$,

$$\mu_{g}\left( t \right)= \mu_{g}\left( 0 \right) (1 - (1-r_{m})[1 - S(t - 1)/S(0)]),$$

where $r_{m}$ is the ratio of the minimum mortality (when the treatment delay is zero) to the baseline mortality (given the treatment delay in the period before 2000). Thus, $\mu_{g}\left( t \right)$ will be close to $\mu_{g}\left( 0 \right)$ when the average treatment delay is close to that before 2000, while $\mu_{g}\left( t \right)$ will be close to ${\mu_{g}\left( 0 \right) r}_{m}$ when the treatment delay is close to zero. We use $S\left( t-1 \right)$ rather than $S\left( t \right)$ in the above equation because $S\left( t \right)$ is only calculated at the end of year *t*.

We estimated the $r_{m}$ parameter using random-effects meta-analysis results based on studies that reported the relative mortality levels in patients with different smear grades (24,83–87) (Table 15). The resulting pooled odds ratio for smear-grade 2+ vs smear-grade <2+ was 1.30 (95% CI: 1.02-1.67) and the odds ratio for smear-grade 3+ vs smear-grade <2+ was 1.68 (24,25,83–89).

Table 15: Odds ratios for mortality in the 2+ and 3+ categories, relative to the <2+ category

| **Study** | **2+ versus <2+** | **3+ versus <2+** |
| --- | --- | --- |
|  | **(Odds ratio, 95% CI)** | **(Odds ratio, 95% CI)** |
| Singla *et al* (24) | 1.28 (0.52-3.17) | 1.97 (0.98-4.30) |
| Vree *et al* (83) | 1.07 (0.58-1.89) | 0.96 (0.43-1.94) |
| Kayigamba *et al* (84) | 2.04 (0.28-22.9) | 4.35 (0.80-43.7) |
| Osawa *et al* (85) | 2.75 (0.97-7.61) | 1.08 (0.38-2.96) |
| Muttath *et al* (86) | 2.33 (0.03-186.4) | 9.16 (1.18-408.6) |
| Kolappan *et al* (87) | 1.27 (0.94-1.73) | 1.81 (1.38-2.37) |
| **Meta-analysis** | **1.30 (1.02-1.67)** | **1.68 (1.26-2.23)** |

We assumed a ‘baseline’ smear grade distribution corresponding to that observed by Singla *et al*. in India (24). In this study, the proportions of TB patients in different smear grades were 0.27 for smear-grade <2+; 0.25 for smear-grade 2+ and 0.48 for smear-grade 3+ (24). We assume the Indian data would be most representative of what might be expected in a resource-limited setting, i.e., with limited screening. We then calculate

$r_{m}=1/(0.27 + 0.25 \times1.30 + 0.48 \times1.68) = 0.71$,

based on the assumption that if there was no treatment delay, all newly treated smear-positive TB cases would have a smear grade <2+.

#### Modelling the effect of age on tuberculosis mortality

Age is an independent predictor of TB mortality both in HIV-infected and HIV-uninfected individuals (71,90–92). The Cape Town-based study by Kaplan *et al*. reported that age is independently associated with TB mortality (adjusted HR 1.28, 95% CI 1.17–1.40, for every 10 year increase) (90). Pepper *et al*. reported similar relative hazards, of approximately 1.5 for every ten year increase of age (71). In most studies assessing TB mortality by age, the higher risk of death is in the 50+ years age categories (71,93,94). In this analysis, we fixed this age effect parameter at a mean of 1.4, where the baseline age to which these effects apply is 55 years. Studies assessing the effect of age on TB mortality were conducted in patients on treatment, and due to the lack of similar studies in untreated TB patients we rely on these same studies in setting assumed age effects for untreated patients.

#### Modelling the effect of human immunodeficiency virus and the use of antiretroviral therapy on tuberculosis mortality

Here we describe how we incorporated the effect of HIV on both treated and untreated TB mortality rates. There are limited studies on the natural history of TB in people living with HIV and most of the studies on TB mortality rates in people living with HIV have been conducted among patients receiving TB treatment. As such, for the purpose of setting the assumptions about the effect of HIV on untreated TB mortality we rely on the literature from treated TB patients.

We first define the relative rate of TB mortality per 50 cell increase in CD4 count in HIV-positive individuals, and estimate this parameter based on the findings from Kaplan *et al.* (90). The study sought to determine changes in TB treatment outcomes among HIV-positive TB individuals; and relied on data of adult TB patients newly registered on the electronic TB register in Cape Town (2009-2013). From this analysis, an increase in 50 CD4 cells/μl was associated with a decrease in mortality risk (HR 0.87, 95% C1 0.84–0.89). A Beta (38.49; 5.75) prior was previously specified for the relative risk of death per 50 cell increase in CD4 count; and standard deviation of 0.05. In a previous calibration analysis, we estimated the posterior mean to be 0.949 (95% CI 0.944 – 0.954). In this analysis, we fixed this parameter at 0.949.

Secondly, we assume there is an effect of HIV viremia on TB mortality (95), which is independent of CD4 count. We estimate this effect of viremia by comparing ART patients to untreated patients, on the assumption that treated patients would mostly be virally suppressed. We define the relative rate of TB mortality if on ART, based on the average of estimates from two studies that reported the effect of ART on TB mortality, Kaplan *et al*. (RR 0.53, 95% CI 0.46 – 0.60) and Pepper *et al*. (RR 0.60, 95% CI 0.50 – 0.70) (71,91). Both studies relied on the electronic tuberculosis treatment register data from the Western Cape, Pepper *et al.* using 2007-2009 data and Kaplan *et al.* using 2010-2011 data (71,91). Taking the average of the estimates from these studies, we specify a Beta (20.72; 16.95) prior with a mean of 0.55 and standard deviation of 0.08.

In the model, mortality for HIV-infected individuals who are not on ART is represented by

$$M\left( x,s \right)={\mu\alpha^{(x-55)/10}\gamma}^{-1}\vartheta^{(s-a)/50},$$

and the mortality for those on ART is represented as by

$$M\left( x,s,d \right)={\mu\alpha^{(x-55)/10}\vartheta}^{(s_{d}-a)/50}$$

where

$x$: age in years

$\mu:$ the mortality rate in HIV-negative individuals

$\alpha:$ increase in TB mortality per 10-year increase in age

$\vartheta:$ relative rate of TB death per 50 cell/μl CD4 increase

$\gamma:$ relative rate of TB mortality for those on ART vs not on ART

$a$: average CD4 count in HIV-uninfected individuals

$s$: CD4 count if untreated, or baseline CD4 count if treated,

$d$: duration on ART

$s_{d}$: current CD4 count for those on ART, with baseline CD4 count s, at a given year of treatment duration $d$.

## Tuberculosis recurrence

Recurrent TB is a TB episode that occurs after the previous TB episode has been considered cured. True relapse occurs when tuberculosis bacilli persist even though bacteriological tests suggested a cure at treatment completion (96). This relapse is viewed as endogenous reactivation of the previous TB strain (96). Factors that contribute to relapse include inadequate regimen and poor adherence. According to most studies, relapse is time-dependent, and the highest risk is within the first 3 – 6 months following successful treatment (97–99).

On the other hand, TB recurrence due to reinfection results from re-exposure to TB and occurs at a relatively constant risk over time (97). The risk of recurrent TB due to reinfection depends on ongoing TB transmission in the community and prevalent factors that increase the risk of fast progression to active TB disease. We note that reinfection in re-treatment individuals is usually measured as the number of new active TB disease cases rather than latent TB infection, thus reflecting fast progression in those experiencing a second (or subsequent) episode of active TB disease (97,99).

Korenromp *et al*. showed that recurrence rates decreased with an increase in follow-up time after treatment completion (97). Among HIV-positive individuals, there were 4.5 recurrences (95% CI 3.2–5.8) per 100-person years, whereas there were 1.9 recurrences (95% CI 1.2–2.7) per 100-person years among HIV uninfected individuals (97). The time trend in reinfection and relapse is more apparent among those without HIV than in those with HIV (97).

### Short-term post-treatment

As evidence suggests that after treatment, the risk of relapse is highest within the first six months following treatment completion (96–98,100); for the model, we set the average time spent in the first post-treatment state to be six months. That is, cured and recovered individuals will move out of the state at a rate of 1/6 per month. We then assume that relapse occurs at an annual rate of 0.1 during the short-term post-treatment state, based on previous Korenromp *et al*. study (97). Due to limited data on the effect of HIV on relapse, we assume no differences in relapse rates between HIV-infected and HIV-uninfected individuals.

### Long-term post-treatment

We handle individuals in this long-term post-treatment state the same way as treatment naïve, latent TB individuals in that they are at risk of both reactivation and reinfection. However, to allow the effect of treatment history, we apply an adjustment factor to the rate of TB incidence and estimate it through calibration. Based on epidemiological studies that have estimated the relative odds/risks of developing TB in treatment-experienced compared to treatment naïve individuals, as a start, we assume this factor is greater than 1.0 and likely to lie in the range 1.8 – 5.9 (101,102). This is based on two studies: firstly Marx *et al.*'s study, which reported a TB prevalence of 3.81% and 2.13% in previously treated and treatment-naïve individuals, respectively, suggesting a relative risk of 1.8 (102). Secondly, Den Boon *et al.*'s study found the prevalence of TB was 10/338 (2.96%) in people who had previously been treated, compared to 16/3145 (0.51%) that do not have the previous TB, suggesting a relative risk of 5.8 (101). We previously specified a Gamma (5.444; 1.556) prior distribution with a mean of 3.5 and a standard deviation of 1.5 for this effect of previous TB. In a previous calibration analysis, we estimated the posterior mean at 3.03 (95% CI 2.74 – 3.23) for this parameter. In this current analysis, we fixed this parameter at 3.03.

For this long-term post-treatment state, the effect of HIV on reinfection in HIV-infected individuals is modelled in the same way as it is applied on fast progression to active TB. Similarly, for reactivation, we apply the HIV effects described earlier.

## Modelling the effect of isoniazid preventative therapy

To incorporate the effect of IPT in the model, we define additional variables. We let $k$ represent the following groups: 0 = uninfected individuals, 1 = latently infected individuals with no TB history, and 2 = previously treated individuals (we do not consider individuals with active TB as IPT would not be recommended for such individuals). We also define $\lambda(t,s,d,k)$ to represent the annual rate of IPT initiation in HIV-positive individuals with CD4 count *s* and ART duration *d* (0 if ART-naïve, 1 for duration <1 year, 2 for duration >1 year), in year $t$. Lastly, define $R\left( k \right)$ as the relative rate of IPT initiation for individuals in TB state $k$ relative to latently infected individuals. The model assumptions on IPT duration and the associated guidelines are shown in Table 16.

Table 16: Isoniazid Preventative Therapy eligibility, requirement, and duration

| **Year** | **Isoniazid Preventative Therapy** **eligibility, requirement, and duration** | **Model assumption** | **Guideline** |
| --- | --- | --- | --- |
| 2010 – 2012 | Eligibility: all HIV-infected individuals with no signs or symptoms suggestive of active TB  TST requirement: TST no longer essential prior to IPT, IPT can be started at the first visit if the patient is asymptomatic.  IPT duration: 6 months of continuous treatment (can be completed over 9 months). | Duration: 6 months (0.5 years), the average IPT treatment completion rate = 1/0.5 years = 2 per year. | 2010, (103) |
| 2013 – present | Eligibility: All people living with HIV with negative symptom-based TB screening.  TST requirement: Use TST if available  IPT duration   - TST positive: 36 months (if pre-ART (CD4>350) / on ART) - TST negative: 6 months (if pre-ART) and 12 months (if on ART) - No TST: 6 months (if pre-ART / on ART) | Duration: 36 months (3 years), then average IPT treatment completion rate = 1/3 years = 0.33 per year. | 2013, (104) |

### Isoniazid preventative therapy initiation by LTBI/TST status

There are limited studies that show the rates of initiation of IPT by TST status among those eligible. Van Ginderdeuren *et al*. reported low rate of TST testing ranging between 0 – 5% at various health facilities (105). The relative rate of IPT initiation in uninfected individuals is highly uncertain. As a start, we set $R\left( 0 \right)=0.5$. We set this assumption as a compromise between the highly optimistic assumption of $R\left( 0 \right)=$ 0 (perfect screening, which accurately distinguishes between LTBI and no LTBI) and the highly pessimistic assumption of $R\left( 0 \right)=$ 1 (no screening or TST produces high rates of false positivity due to BCG exposure). For simplicity, we assume rates of IPT initiation are the same in individuals who have never been treated for TB and those who have previously been treated (i.e*., $R\left( 1 \right)$=* $R\left( 2 \right)=1).$ We also assume that there is no IPT initiation in individuals on TB treatment and individuals with active TB.

### Isoniazid preventative therapy initiation by CD4 count

The second parameter we define is $H(s),$ the relative rate of IPT initiation in CD4 category *s*, relative to that at CD4 counts <200 cells/μl. A study conducted in Gauteng found that rates of IPT initiation at CD4 counts ≥500 cells/μl were significantly lower than those at CD4 counts <500 cells (adjusted OR 0.46, 95% CI: 0.24-0.82) (105). Therefore, to be consistent with this study and the Thembisa HIV model assumptions on relative rates of ART initiation in different CD4 categories (4), we set the relative rates of IPT initiation to be 0.4 at CD4 counts ≥500, 0.5 at CD4 counts 350-499 and 0.7 at CD4 counts 200-349 (in all cases, relative to CD4 <200 cells/μl).

### Isoniazid preventative therapy initiation by ART status

The third parameter that we define is $P(d),$ the relative rate of IPT initiation at ART duration *d* when compared to ART-naïve individuals (by definition,$P(0)=1)$. The Gauteng study cited previously found that individuals on ART had a higher rate of IPT initiation (OR 2.03, 95% CI: 0.88-5.87) and that approximately half of all IPT initiations after ART initiation occurred in the first year of ART (the median time between ART initiation and IPT initiation was 374 days) (105). The Thembisa model for Gauteng estimates that over the 2015-16 period (the period when the study was conducted), approximately 14% of adult ART patients had started ART in the last year. The finding that 50% of IPT in ART patients was initiated in the first year of ART thus implies that $0.5=0.14 \times P(1) / [0.14 \times P(1)+0.86 \times P(2)].$ Solving this equation, we get a $P(1)/P(2)$ ratio of 0.16. The finding of an OR of 2.03, in turn, implies that $2.03\approx0.14\times P(1)+0.86\times P(2)$. Substituting the $P(2)/P(1)$ ratio into this equation, we get $P(1)=7.3$ and $P(2)=1.2$.

Having defined the intermediate variables, we calculate

$\lambda(t,s,d,k) = \lambda(t) H(s) P(d) R(k)$*,*

where $\lambda(t)$ is the rate of IPT initiation in year *t* in the ‘base’ category (i.e. ART-naïve, HIV diagnosed adults with latent TB infection). We estimate this base rate from the reported numbers of IPT initiations in year *t*, $T(t)$. If $N(t,s,d,k)$ is the model estimates of the number of HIV-diagnosed adults at the start of year *t*, in CD4 category *s*, with ART duration *d*, in TB state *k* (excluding individuals with active TB and treated for TB, and excluding individuals who are already on IPT), then

$$T\left( t \right)=\lambda\left( t \right)\sum_{s} \sum_{d} \sum_{k} N\left( t,s,d,k \right)H\left( s \right)P\left( d \right)R\left( k \right)$$

$\lambda(t)$ is solved for, by rearranging the terms in this equation above. $T(t)$ values are taken from the District Health Information System (DHIS) (Table 17). Due to the frequent changes in guidelines and barriers to implementation of IPT policies at health care facilities, data on IPT uptake has been limited, and the quality may be affected as well, particularly in the earlier years (before 2010). A significant shift in the guidelines was made in 2010 where the strict requirement for a positive TST to be eligible to initiate IPT was removed (106). We assume zero IPT uptake before 2010, as the DHIS data suggest minimal IPT before 2010; these data were available until 2016-17. For years 2017 and beyond, IPT uptake in the baseline category ($\lambda(t)$) was linearly interpolated between the 2016-17 rate and a rate of 1.2% in 2021.

Table 17: Number of HIV-infected new eligible individuals initiated on isoniazid preventative therapy by province

| Year | 2010-11 | 2011-12 | 2012-13 | 2013-14 | 2014-15 | 2015-16 | 2016-17 |
| --- | --- | --- | --- | --- | --- | --- | --- |
| Number of patients initiating IPT | 244888 | 379063 | 385338 | 427336 | 422541 | 385007 | 396915 |

Source: District Health Information System

### Isoniazid preventative therapy completion/drop-out

Based on the Southern African studies assessing completion over six months of treatment and the reported completion proportions, IPT drop-out ranged between 6% - 37% (107–111). By taking the median of the five studies, 13.2%, the drop-out rate over six months is roughly ~0.024 per month. The recommendations for IPT eligibility have changed over time. For the years between 2010 and 2012, we assume six months; from 2013, we assume 36 months. Using the 0.024 dropout rate per month, the average net time on IPT is then 5.2 months = (1/(0.024 + 1/6)) in the period up to 2012. Similarly, after 2012 we assume the average time on IPT is 19.3 months (1/(0.024 + 1/36)).

### Effectiveness of isoniazid preventative therapy

We set the effectiveness of IPT at 52%, based on the estimated RR of 0.48 (95% CI 0.29 – 0.82) among LTBI individuals (those with a positive TST) in the meta-analysis by Ayele *et al.* (112). However, among individuals with no LTBI (negative TST), the effect of IPT was inconclusive (RR 0.79 [95% CI 0.58 – 1.08]) (112); as such, we assume 0% effectiveness for this group. In addition, we assume that when an individual stops taking IPT, there will be no effect of IPT, as shown by Churchyard *et al.* (113).

## Modelling the effect of tuberculosis risk factors on tuberculosis incidence

To explore factors that could be important in explaining age and sex differences in TB incidence, we considered the following risk factors: poorly controlled diabetes, undernutrition/underweight, tobacco smoking and alcohol abuse. These risk factors are selected based on their established effect of increasing the risk of developing TB disease and their relatively high prevalence in the South African population (Table 18). In computing the cumulative effect of these risk factors, we assume the effects are independent.

Table 18: Age- and sex-specific prevalence (%) of risk factors: HbA1c > 6.5%, underweight, alcohol abuse and tobacco smoking

|  | HbA1c > 6.5% | | Underweight (BMI < 18.5 kg/m²)) | | Alcohol abuse | | Current smoking | |
| --- | --- | --- | --- | --- | --- | --- | --- | --- |
| Age | Males | Females | Males | Females | Males | Females | Males | Females |
| 15-24 | 2.0 | 0.9 | 22.05 | 9.7 | 20.7 | 5.1 | 29.5 | 4.95 |
| 25-34 | 3.4 | 4.5 | 6.4 | 3.3 | 36.1 | 6.1 | 43.50 | 7.80 |
| 35-44 | 6.6 | 11.8 | 11.4 | 2.8 | 31.8 | 6.0 | 43.90 | 7.00 |
| 45-54 | 11.7 | 20.8 | 8.2 | 2.1 | 27.8 | 4.4 | 45.00 | 11.0 |
| 55-64 | 23.0 | 28.7 | 12.4 | 2.9 | 25.7 | 3.7 | 37.80 | 11.3 |
| 65-90 | 21.1 | 30.1 | 6.0 | 3.7 | 20.9 | 2.0 | 24.90 | 7.80 |

BMI=body mass index. HbA1c= Glycated hemoglobin. Data sources. For underweight (BMI < 18.5) SANHANES-1 survey (114); For HbA1c > 6.5%, alcohol abuse, and current smoking: 2016 SADHS (115).

### Poorly controlled diabetes (HbA1c > 6.5%)

Individuals with diabetes have a 3.59-fold increased risk of TB disease compared to those without diabetes, as estimated by Al-Rifai *et al*. in a meta-analysis and systematic review (116). This estimate is based on studies which defined uncontrolled diabetes as HbA1c > 6.5% or HbA1c > 7.0% or Fasting Blood Glucose > 120 mg/dl. We use the 2016 SADHS data for HbA1c > 6.5% prevalence to be relatively consistent with these definitions (115). In the model, we defined the effect of diabetes as an increase in TB incidence due to having diabetes (HbA1c > 6.5%), set at $\theta_{1}$= 2.59 for (risk factor $i=1)$. Given the age- ($x$) and sex- ($g$) specific prevalence ($p_{1gx}$) of poorly controlled diabetes, the multiplicative increase in TB incidence due to diabetes in individuals of age x and sex g (relative to a hypothetical population in which there is no diabetes) is calculated as:

$RR_{1gx}=1.0 +p_{1gx}\theta_{1}$.

### Underweight (BMI <18.5 kg/m²)

The meta-analysis of Lönnroth *et al*. (117) suggests that TB incidence declines steadily as BMI increases. We set the effect of low BMI on the risk of developing TB disease based on Leung *et al.*, which was a cohort study of 42 116 individuals who were 65 years or older enrolled in health centres across Hong Kong, China (118). The relative risk for culture-confirmed TB was 2.21 when not excluding other potential TB risk factors (this is likely to be an upper bound on the true effect of low BMI, due to confounding with smoking and other factors); and 1.39 when excluding TB risk factors (probably a lower bound because baseline is defined as normal BMI, not all BMI >18.5 kg/m²); 1.8 is the midpoint between these two (105). Although this study may have been conducted in a relatively older population, they adjusted for most confounders, including smoking, alcohol, diabetes, sex and age (unlike the Lönnroth *et al.* meta-analysis).

Based on Leung *et al.* we fixed the parameter for the increase in TB risk due to low BMI ($\theta_{2}$) at 0.8 (118). The age- ($x$) and sex- ($g$) specific prevalence ${(p}_{2gx})$ for underweight is based on the SANHANES-1 survey (Table 18) (114). In the model, the age- and sex-specific multiplicative increase in TB incidence due to underweight is calculated as

$RR_{2gx}=1.0 +p_{2gx}\theta_{2}$.

### Tobacco smoking

Given that the effect of tobacco smoking on the risk of developing active TB depends on current exposure and duration of smoking, we incorporate both effects (current exposure and duration) in the model. The effects were estimated from a case-control study in India (119). In the study, men aged 20-50 years with TB were randomly matched by age with non-TB controls. The estimated odds ratios of active TB for smokers with <10 years, 11–20 years, and >20 years of smoking were 1.72, 2.45, and 3.23, respectively, compared to non-smokers (119).

Using this study by Kolappan and Gopi, we estimate the effect of current exposure to smoking (risk factor $i=3$) is a 1.47-fold increase in TB risk, and the TB risk increases by a factor of 1.38 per ten years of smoking. In the model, we set the increase in TB risk if currently smoking at $\theta_{3,1}$= 0.47 and the increase in TB risk per 10-year increase in the duration of smoking at $\theta_{3,2}$= 0.38. The multiplicative increase in TB incidence due to smoking is calculated as follows:

$$RR_{3gx}=\left( 1.0 +\theta_{3,1}p_{3gx} \right)\left( 1+\theta_{3,2} \right)^{smd\left( g,x \right)/10}$$

$p_{3gx}$ is represents the current smoking prevalence and $smd(g,x)$ is the average smoking duration calculated for each sex $(g$) and age $(x$). We used the age-specific prevalence of current smoking to generate the distribution of duration of smoking. We assumed there is no smoking before the age of 15 years. The $smd(g,x)$ was calculated as follows:

- First, note that current smoking prevalence ($p_{3gx}$) is given in age categories as obtained from the SADHS (Table 18) (115).
- We assume that the prevalence of current smoking represents the prevalence at the median age of each category.
  - e.g., for females aged 20-24 years, the prevalence of 5.8% represents current smoking for females aged 22 years.
- Then we linearly interpolated between the mid-points in each age category to get prevalence estimates for the other ages.
- The overall average smoking duration at each subsequent age is calculated as the cumulative smoking prevalence for all previous years to the age 15, e.g.

$$smd(1,23) = \sum_{x=15}^{22} p_{3,1,x}$$

### Alcohol abuse

The estimated relative effect of alcohol abuse on developing TB disease is almost 3-fold (relative risk 2.94, 95% CI: 1.89-4.59) (120). In this meta-analysis (120), alcohol use disorder/abuse was defined as drinking at least 40g alcohol per day (121), equivalent to four drinks. The 2016 South African DHS defined risky alcohol consumption as consuming at least five drinks on one occasion in the past 30 days (115). In this survey, 28% males and 5% females reported risky drinking habits (Table 18) (115).

We specified the effect of heavy alcohol consumption on TB incidence to be $\theta_{4}=$1.94, based on Lonroth *et al*.'s review (120). In addition, we assume the age- ($x$) and sex- ($g$) specific prevalence ${(p}_{4gx})$ of alcohol abuse (binge drinking) as presented in the SADHS Table 18 (115). The age- and sex-specific multiplicative increase in TB incidence due to alcohol abuse is calculated as:

$$RR_{4gx}=1.0 +p_{4gx}\theta_{4}$$

### Combined effect of TB risk factors

The overall adjustment in the model (relative to a hypothetical population in which there are no TB risk factors) is computed as the cumulative multiplicative effect of the risk factors ($i=1,\ldots, 4$) described above (${RR}_{igx}$):

$$F_{gx}=\prod_{i=1}^{4} RR_{igx}$$

$F_{gx}$ represents the relative risk of TB in people of sex *g* and age *x* due to diabetes, underweight, smoking, and alcohol abuse compared to individuals of the same age and sex with none of these risk factors. These $F_{gx}$ are applied to the rate of progression from infection to active TB disease in the model.

Table 19 shows examples of $F_{gx}$ factors for select ages 15 to 40 years, calculated from the means of the prior distributions specified previously.

Table 19: Cumulative age- and sex- effect of selected tuberculosis risk factors HbA1c > 6.5%, underweight, smoking and alcohol abuse

|  | HbA1c > 6.5 $(\theta_{1}=2.59)$ | | Underweight (BMI < 18.5) $(\theta_{2}=0.8)$ | | Current smoking  (  ) | | Alcohol abuse  ($\theta_{4}=1.94)$ | | Overall | |
| --- | --- | --- | --- | --- | --- | --- | --- | --- | --- | --- |
| Age | Male | Females | Male | Females | Male | Females | Male | Females | Male | Females |
| 15 | 1.052 | 1.023 | 1.479 | 1.281 | 1.031 | 1.007 | 1.229 | 1.031 | 1.970 | 1.361 |
| 16 | 1.052 | 1.023 | 1.479 | 1.281 | 1.063 | 1.014 | 1.229 | 1.031 | 2.032 | 1.371 |
| 17 | 1.052 | 1.023 | 1.479 | 1.281 | 1.098 | 1.022 | 1.229 | 1.031 | 2.100 | 1.382 |
| 18 | 1.052 | 1.023 | 1.327 | 1.073 | 1.128 | 1.025 | 1.229 | 1.031 | 1.935 | 1.161 |
| 19 | 1.052 | 1.023 | 1.327 | 1.073 | 1.160 | 1.028 | 1.229 | 1.031 | 1.989 | 1.164 |
| 20 | 1.052 | 1.023 | 1.327 | 1.073 | 1.193 | 1.032 | 1.592 | 1.167 | 2.652 | 1.322 |
| 21 | 1.052 | 1.023 | 1.327 | 1.073 | 1.230 | 1.035 | 1.592 | 1.167 | 2.732 | 1.326 |
| 22 | 1.052 | 1.023 | 1.327 | 1.073 | 1.268 | 1.039 | 1.592 | 1.167 | 2.818 | 1.331 |
| 23 | 1.052 | 1.023 | 1.327 | 1.073 | 1.287 | 1.042 | 1.592 | 1.167 | 2.860 | 1.335 |
| 24 | 1.052 | 1.023 | 1.327 | 1.073 | 1.306 | 1.045 | 1.592 | 1.167 | 2.902 | 1.339 |
| 25 | 1.088 | 1.117 | 1.117 | 1.060 | 1.326 | 1.048 | 1.700 | 1.118 | 2.740 | 1.388 |
| 26 | 1.088 | 1.117 | 1.117 | 1.060 | 1.346 | 1.052 | 1.700 | 1.118 | 2.781 | 1.393 |
| 27 | 1.088 | 1.117 | 1.117 | 1.060 | 1.366 | 1.056 | 1.700 | 1.118 | 2.823 | 1.398 |
| 28 | 1.088 | 1.117 | 1.117 | 1.060 | 1.387 | 1.059 | 1.700 | 1.118 | 2.866 | 1.402 |
| 29 | 1.088 | 1.117 | 1.117 | 1.060 | 1.408 | 1.063 | 1.700 | 1.118 | 2.910 | 1.407 |
| 30 | 1.088 | 1.117 | 1.117 | 1.060 | 1.430 | 1.067 | 1.700 | 1.118 | 2.955 | 1.413 |
| 31 | 1.088 | 1.117 | 1.117 | 1.060 | 1.450 | 1.069 | 1.700 | 1.118 | 2.997 | 1.416 |
| 32 | 1.088 | 1.117 | 1.117 | 1.060 | 1.471 | 1.071 | 1.700 | 1.118 | 3.040 | 1.419 |
| 33 | 1.088 | 1.117 | 1.117 | 1.060 | 1.492 | 1.074 | 1.700 | 1.118 | 3.084 | 1.421 |
| 34 | 1.088 | 1.117 | 1.117 | 1.060 | 1.514 | 1.076 | 1.700 | 1.118 | 3.128 | 1.424 |
| 35 | 1.171 | 1.306 | 1.208 | 1.051 | 1.535 | 1.078 | 1.617 | 1.116 | 3.247 | 1.652 |
| 36 | 1.171 | 1.306 | 1.208 | 1.051 | 1.557 | 1.080 | 1.617 | 1.116 | 3.294 | 1.655 |
| 37 | 1.171 | 1.306 | 1.208 | 1.051 | 1.580 | 1.082 | 1.617 | 1.116 | 3.341 | 1.658 |
| 38 | 1.171 | 1.306 | 1.208 | 1.051 | 1.602 | 1.084 | 1.617 | 1.116 | 3.666 | 1.661 |
| 39 | 1.171 | 1.306 | 1.208 | 1.051 | 1.625 | 1.086 | 1.617 | 1.116 | 3.719 | 1.665 |
| 40 | 1.171 | 1.306 | 1.208 | 1.051 | 1.649 | 1.089 | 1.617 | 1.116 | 3.772 | 1.668 |

BMI=body mass index. HbA1c= Glycated hemoglobin.

## Calibration data sources and defining likelihoods

We used a Bayesian approach to calibrate the model and estimate various parameters. Because our model is slow to run, and because the Bayesian calibration process is particularly slow to converge when there are many parameters being included in the uncertainty analysis, we calibrated the model through a series of steps. We first (in a previous calibration analysis) estimated the TB transmission and natural history parameters that gave the best fit to South African TB data, using the same likelihood definitions as given below. In this present analysis, we considered the parameters that determine the impact of TB interventions. The prior means and standard deviations are summarized in Table 20, with the posterior means and 95% confidence intervals for the parameters varied and estimated in the previous calibration analysis. We also indicate the parameters varied in this present analysis.

Table 20: Summary of model parameters with prior means and standard deviations, posterior means and 95% confidence intervals from the previous calibration analsyis, and summary of those varied in this present analysis

| **Parameter description** | **Mean** | **Standard deviation** | **Posterior means (95% CI) from previous calibration** | **Parameters varied in the current analysis** |
| --- | --- | --- | --- | --- |
| TB transmission probability per contact per day (if infectious individual is smear-positive) | 0.0025 | 0.0025 | 0.0030 (0.0026–0.0034) | ✓ |
| The annual rate of reactivation in HIV-negative individuals | 0.0024 | 0.0012 | 0.00148 (0.0014–0.00155) |  |
| Relative rate of TB incidence per 100 cell increase in CD4 | 0.71 | 0.085 | 0.703 (0.693–0.712) |  |
| Annual recovery rate in smear-positive TB, HIV-negative individuals | 0.09 | 0.02 | 0.075 (0.067–0.081) |  |
| Annual recovery rate in smear-negative TB, HIV-negative individuals | 0.24 | 0.05 | 0.224 (0.198–0.247) |  |
| Relative infectivity of smear-negative TB compared to smear-positive individuals | 0.22 | 0.03 | 0.206 (0.196–0.218) |  |
| Increase in TB risk if previously experienced TB | 3.50 | 1.5 | 3.03 (2.55–3.53) |  |
| Smear-negative TB mortality (untreated) | 0.061 | 0.012 | 0.049 (0.046–0.052) |  |
| Smear-positive TB mortality (untreated) | 0.212 | 0.042 | 0.196 (0.174–0.221) |  |
| The relative rate of TB mortality per 50 cell increase in CD4 count if HIV+ | 0.87 | 0.05 | 0.949 (0.944–0.954) |  |
| Proportion with cough >2 weeks in individuals with smear-negative TB | 0.2 | 0.1 | 0.198 (0.149–0.263) |  |
| The proportion of incident TB cases in HIV-negative adults that are smear-positive | 0.52 | 0.1 | 0.51 (0.48–0.54) |  |
| Relative ratio of symptoms in patients with smear-positive TB, compared to smear-negative TB | 2.2 | 0.5 | 3.03 (2.74–3.23) |  |
| Relative rate of TB incidence for those on ART (controlling for CD4) | 0.81 | 0.05 |  | ✓ |
| Relative rate of TB mortality if on ART | 0.55 | 0.08 |  | ✓ |
| The annual rate of health-seeking in males with smear-negative TB | 2.14 | 0.49 |  | ✓ |
| The annual rate of health-seeking in males in the general population | 1.15 | 0.5 |  | ✓ |
| The annual rate of health-seeking in males due to TB-like symptoms | 0.22 | 0.15 |  | ✓ |
| The proportion of active TB cases seeking treatment who are treated empirically before any microbiological test is done | 0.125 | 0.144 |  | ✓ |
| The proportion of smear-negative TB cases which are treated empirically if they initially screened negative on a smear test | 0.33 | 0.236 |  | ✓ |
| Relative rate of empirical treatment if not seeking treatment because of TB symptoms | 0.5 | 0.289 |  | ✓ |
| Relative rate of empirical treatment if symptoms are not due to TB | 0.5 | 0.289 |  | ✓ |
| Relative rate of health-seeking in women, compared to men | 1.55 | 0.17 |  | ✓ |
| Relative rate of health-seeking in HIV-positive compared to HIV-negative individuals | 3 | 1 |  | ✓ |
| Relative rate of screening in TB patients seeking treatment for TB symptoms, compared to those seeking treatment for other conditions |  |  |  |  |
| Initial (up to 2005) | 8.71 | 2.5 |  | ✓ |
| Ultimate (after 2012) | 4 | 1.2 |  | ✓ |

ART = antiretroviral therapy; TB=tuberculosis. Ticks indicate the parameters which were varied in the respective steps.

In this section we describe the likelihood functions to represent the goodness of fit to the calibration targets. The model calibration targets included the numbers of recorded TB deaths; numbers of TB cases initiated on TB treatment; the proportion of cases in the electronic TB register that are HIV-positive; the proportion patients dying during TB treatment recorded in the electronic TB register; the number of laboratory tuberculosis tests (52) and the prevalence of TB (46).

### The likelihood for recorded number of tuberculosis deaths

#### Cleaning the mortality data

To get the number of recorded deaths on the South African vital register, we considered deaths where TB is the underlying cause of death. These TB deaths are broadly classified using the codes A15-19. We also included deaths for which HIV was recorded as the broad underlying cause of death (codes B20-B24), and TB is listed as a contributing cause of death (i.e., HIV underlying cause AND TB is either first, second, third, or fourth contributing cause of death). For TB deaths with unknown/unspecified age and sex, we adjusted by proportionally distributing the unknown age and sex deaths to the age and sex categories in which TB deaths were recorded most frequently.

We also adjusted for 1) incomplete reporting of deaths (the deaths that do not get documented) and 2) ill-defined causes of deaths. To adjust for the incompleteness of reported deaths, we applied age-specific completeness proportions previously computed by Johnson et al., which change over time (4). ICD codes R00-R99 represents missing and garbage codes. For each sex and 5-year age group, the total adjusted number of TB deaths was computed using the following expression:

$$\frac{T+H}{\left( 1-\frac{M}{N} \right)C}$$

where T is the number of deaths for which TB (A15-19) is the recorded underlying cause of death. H is the total number of deaths for which HIV (B20-B24) is recorded as the underlying cause of death and TB is recorded as a contributing cause of death (i.e. TB (A15-19) is Cause A or Cause B or Cause C or Cause D). N represents all the deaths recorded in the vital register. M represents the missing and garbage code. C represents completeness, which is the proportion of deaths that get recorded. This completeness ratio was computed as a ratio of the recorded SA deaths (N) and the total number of deaths produced by the Thembisa 4.1 model (O) (Table 21) (4). These adjustments are shown in Table 21, and the last column (Y) represents the total adjusted deaths.

Figure 5: Flow for cleaning mortality data from the vital register

**Overall adjustment**

$$Y=\frac{T+H}{\left( 1-\frac{M}{N} \right)C}$$

**Extracted mortality data from Stats SA database**

<http://nesstar.statssa.gov.za:8282/webview/>

**Variables extracted**

**T:** TB (ICD codes A15-A19) deaths, the main cause of death.

**H:** HIV (ICD codes B20-B24) is the underlying cause of death, TB listed as 1^st^, 2^nd^, 3^rd^ or 4^th^ contributing cause.

**M:** missing and garbage code (ICD codes R00-R99).

Additional fields: age, sex, years: 1997 – 2016

**Proportional distribution of unspecified age and sex.**

**Estimated completeness (C = N/O)**

**N:** total (annual) number of deaths recorded in the vital register

**O**: estimated (annual) *total* number of deaths (Thembisa)

Table 21: Number of tuberculosis deaths and adjustments

| **Death year** | **TB Underlying cause of death**  **(T)** | **HIV is the underlying cause, and TB contributing**  **(H)** | **Missing/garbage code (R00-R99)**  **(M)** | **Total recorded deaths**  **(N)** | **Thembisa Total deaths output**  **(O)** | **Completeness: % total deaths that get recorded**  **(C)** | **Overall adjusted TB deaths**  **(Y)** |
| --- | --- | --- | --- | --- | --- | --- | --- |
| 1997 | 22152 | 1801 | 42399 | 323854 | 369395 | 0,88 | 31437 |
| 1998 | 28656 | 2164 | 51165 | 374351 | 408861 | 0,95 | 38990 |
| 1999 | 34377 | 2858 | 47436 | 394656 | 423352 | 0,93 | 45399 |
| 2000 | 42581 | 2945 | 52334 | 429825 | 456393 | 0,94 | 55042 |
| 2001 | 51444 | 2605 | 58221 | 470502 | 493844 | 0,95 | 64742 |
| 2002 | 60715 | 3168 | 63448 | 516700 | 528490 | 0,98 | 74487 |
| 2003 | 68251 | 3301 | 70762 | 573139 | 570371 | 1,00 | 81236 |
| 2004 | 71034 | 3985 | 71547 | 594299 | 616324 | 0,96 | 88447 |
| 2005 | 74767 | 4731 | 75078 | 613506 | 652376 | 0,94 | 96322 |
| 2006 | 77775 | 5240 | 83818 | 628632 | 658129 | 0,96 | 100281 |
| 2007 | 77292 | 4974 | 84855 | 620595 | 652047 | 0,95 | 100126 |
| 2008 | 75542 | 5765 | 82084 | 613239 | 635166 | 0,97 | 97229 |
| 2009 | 70226 | 7222 | 80675 | 598291 | 616844 | 0,97 | 92295 |
| 2010 | 63668 | 7633 | 74955 | 566712 | 595311 | 0,95 | 86316 |
| 2011 | 55489 | 7153 | 70690 | 531840 | 567092 | 0,94 | 77033 |
| 2012 | 48825 | 8290 | 67285 | 509994 | 536539 | 0,95 | 69220 |
| 2013 | 42041 | 10075 | 60746 | 492261 | 525201 | 0,94 | 63431 |
| 2014 | 39695 | 9083 | 59117 | 492048 | 520086 | 0,95 | 58598 |
| 2015 | 34042 | 7165 | 58289 | 487607 | 517478 | 0,94 | 49669 |
| 2016 | 29513 | 6576 | 60335 | 468573 | 514123 | 0,91 | 45450 |

C = N/O; Y= (T + H)/((1 – M/N)C).

#### The likelihood for recorded number of tuberculosis death

We consider adult deaths at ages 20 years and older, disaggregated by sex. We assume the number of recorded TB deaths follows a Log-Normal distribution and specify the likelihood of observing the recorded number of deaths if the model represents the expected number of deaths. We assume the log-normal distribution as it is appropriate in modelling non-negative values, and the data may have large variance due to both random and systematic biases in the data reporting/handling.

We let $D_{g,t}$ represent the number of recorded deaths from the vital register (after adjustment, as described in the previous section), in individuals of sex $g$, each year $t$. Let $M_{g,t}\left( \phi\right)$ represent the model estimates for the numbers of deaths for individuals with sex $g$ per year $t$. We let $\boldsymbol{\phi}$ represent the set of input parameter values.

A review and meta-analysis assessing causes of deaths using autopsies, reported that TB accounted for 37.2% (95% CI 25.7–48.7%) of HIV-related deaths and that 45.8% (95% CI 32.6–59.1%) of TB remains undiagnosed at the time of death. Among African studies, the pooled estimate was 43.2% (95% CI 38.0–48.3) (122). In the model we introduced the parameter $\gamma$(a ratio of model the estimated (‘true’) tuberculosis cases to the number of recorded deaths classified as TB) to correct for potential bias in the recorded TB death data. By this, we are assuming that the bias is relatively stable over time, and similar for males and females.

We estimated $\gamma$ as

$$\gamma=\frac{\sum_{g} \sum_{t} M_{g,t}}{\sum_{g} \sum_{t} D_{g,t}}$$

Then we specify the likelihood function for TB deaths as

$$L\left( \boldsymbol{D} | \phi\right)= \prod_{g} \prod_{t=1997}^{2016} \frac{1}{\sqrt{2\pi\sigma^{2}}}\exp\left( - \frac{\left[ \ln\left( D_{g,t}\gamma\right)-\ln\left( M_{g,t}\left( \phi\right)) \right) \right]^{2}}{2\sigma^{2}} \right)$$

And the variance ($\sigma^{2}$) was set at 0.01.

### The likelihood for expected tuberculosis deaths in people living with HIV

##### Estimating the number of HIV deaths that are due to tuberculosis

In the model, we approximate the expected number of HIV deaths due to TB using the proportion of 0.43 estimated by the Gupta *et al.* meta-analysis for African settings (122) (described in the previous section 11.1). Then, the proportion 0.43 is applied to the total AIDS deaths produced by the Thembisa 4.4 model (5). (The Thembisa model has been calibrated to all-cause mortality data, stratified by age and sex, and has been validated against estimates of AIDS deaths from the National Burden of Disease study (123), and these estimates of total AIDS deaths are produced independently of the TB model.) These expected numbers are shown in Table 22.

##### Definition of the likelihood for expected tuberculosis deaths in people living with HIV

Let $D_{t}$ represent the number of TB deaths in adults living with HIV, in year $t$. These $D_{t}$ are not pure data obtained from a specific source; we approximated them by applying the 0.43 proportion of expected TB deaths in HIV individuals estimated by the Gupta *et al.* meta-analysis (122) to the total AIDS deaths produced by the Thembisa 4.4 model (Table 22). We consider only deaths over the 1997-2010 period, (a) because Thembisa is not calibrated to vital registration data before 1997, and (b) because the meta-analysis of African studies only covers data collected up to 2010 and it is possible that the proportion of deaths in PLHIV that are due to TB might be different in the post-2010 period because of greater ART uptake.

Then let $E_{t}(\phi)$ be model estimate of the number of TB deaths in adults who are HIV positive, where $\phi$ represents the set of model input parameters. Then we specify a Log-Normal likelihood function as follows

$$L\left( \boldsymbol{D} | \phi\right)=\prod_{t=1997}^{2010} \frac{1}{\sqrt{2\pi\sigma_{t}^{2}}}\exp\left( - \frac{\left[ \ln\left( D_{t} \right)-\ln\left( E_{t}(\phi)) \right) \right]^{2}}{2\sigma_{t}^{2}} \right)$$

The variance ($\sigma_{t}^{2}$) was estimated[[2]](#footnote-3) from the confidence intervals of AIDS deaths estimated in Thembisa 4.4 (5) and from the confidence intervals for the proportion of expected TB deaths in HIV individuals (0.43 (95% CI: 0.38 – 0.48)) (122), all converted to the log scale.

Table 22: Expected number of tuberculosis deaths in people living with HIV

| **Year** | **Thembisa 4.4 AIDS deaths** | **TB deaths in HIV-positive ($D_{t}$)** | **Standard errors*** |
| --- | --- | --- | --- |
| 1997 | 52 149 | 22424 | 0.1733 |
| 1998 | 71 563 | 30772 | 0.1725 |
| 1999 | 94 202 | 40507 | 0.1714 |
| 2000 | 119 458 | 51367 | 0.1710 |
| 2001 | 143 624 | 61758 | 0.1704 |
| 2002 | 168 832 | 72598 | 0.1701 |
| 2003 | 193 813 | 83339 | 0.1698 |
| 2004 | 220 214 | 94692 | 0.1696 |
| 2005 | 228 122 | 98092 | 0.1697 |
| 2006 | 215 892 | 92833 | 0.1705 |
| 2007 | 192 528 | 82787 | 0.1718 |
| 2008 | 172 663 | 74245 | 0.1719 |
| 2009 | 157 785 | 67847 | 0.1711 |
| 2010 | 142 992 | 61487 | 0.1706 |

*On log scale, estimated from the upper and lower limits of the proportion of expected TB deaths (Gupta estimate) and Thembisa AIDS deaths.

### The likelihood for recorded number of tuberculosis cases initiated on treatment

We use the number of notified TB deaths from the ETR, accessed through and cleaned by the Desmond Tutu TB/HIV Centre (Table 23) (94). The database relies on data from TB Blue cards - the primary medical record for people who have been diagnosed with TB and have initiated treatment (124). This information gets transcribed into the TB register, and is then fed into the sub-district, district, provincial and national TB registers. Some of the challenges of the ETR include the under-reporting of TB cases, duplicates of patient data, and losses to follow-up due to deaths or transfers of patients to other facilities (124).

Table 23: Number of tuberculosis cases initiated on treatment by year, sex and HIV status

| **Year** | **Sex** | **HIV-positive** | **HIV-negative** | **Total** |
| --- | --- | --- | --- | --- |
| 2004 | Female | 91 112 | 31 645 | 280 611 |
|  | Male | 99 295 | 58 559 |  |
| 2005 | Female | 93 769 | 43 727 | 308 483 |
|  | Male | 90 448 | 80 539 |  |
| 2006 | Female | 97 209 | 52 545 | 328 756 |
|  | Male | 94 345 | 84 657 |  |
| 2007 | Female | 114 842 | 46 414 | 350 339 |
|  | Male | 114 409 | 74 674 |  |
| 2008 | Female | 134 549 | 49 640 | 392 695 |
|  | Male | 130 061 | 78 445 |  |
| 2009 | Female | 142 000 | 51 989 | 414 277 |
|  | Male | 137 783 | 82 505 |  |
| 2010 | Female | 139 652 | 51 977 | 408 213 |
|  | Male | 135 793 | 80 791 |  |
| 2011 | Female | 131 416 | 54 730 | 400 317 |
|  | Male | 129 912 | 84 259 |  |
| 2012 | Female | 115 005 | 51 266 | 366 730 |
|  | Male | 119 516 | 80 943 |  |
| 2013 | Female | 102 440 | 51 371 | 348 674 |
|  | Male | 112 391 | 82 472 |  |
| 2014 | Female | 93 737 | 48 878 | 332 352 |
|  | Male | 108 452 | 81 285 |  |
| 2015 | Female | 81 389 | 44 769 | 299 883 |
|  | Male | 97 592 | 76 133 |  |
| 2016 | Female | 70 298 | 38 361 | 265 917 |
|  | Male | 88 696 | 68 562 |  |

##### Likelihood for tuberculosis cases recorded on the electronic tuberculosis treatment register

We assume that the number of TB cases initiated on treatment follows a Log-Normal distribution. The analysis is restricted to the years 2004 to 2016 as we were granted access to the cleaned dataset (2004-2016). In recent years, the National Department of Health of South Africa has made a shift from using the ETR system to the district health information system (DHIS) (125). We calibrated only to sex-stratified data; although the data includes age, we did not calibrate to age-stratified recorded TB cases.

We let $E_{g,t}$ represent the number of people initiated on TB treatment as recorded in the ETR, for individuals of sex *g*, and each year $t$ (Table 23). These ETR data are subject to biases. For instance, there may be under-reporting because in most cases the ETR does not include TB cases in tertiary care, and, the ETR only includes drug-sensitive TB (124,126). Also, the ETR does not include TB cases treated in the private sector; it is estimated that approximately 8% of symptomatic TB cases who seek treatment, seek in the private sector (46). We therefore apply an adjustment factor $(\gamma)$ defined as the ratio of the true number of TB cases receiving treatment to the number of TB cases recorded in the ETR. We let $D_{g,t}\left( \phi\right)$ represent the model estimate for the numbers of people initiated on TB treatment for individuals with sex $g$, in year $t$; where $\boldsymbol{\phi}$ represents the set of input parameters values. We then estimated $\gamma$ as follows

$$\gamma= \frac{\sum_{g} \sum_{t} D_{g,t}}{\sum_{g} \sum_{t} E_{g,t}}$$

We restricted the factor $\gamma$ to range between 1.0 and 1.3 based on the studies which have attempted to estimate the ETR data bias (124,127). From the model, we estimated $\gamma$ to be 1.08 (95% CI 1.01 – 1.18).

Then the likelihood function is given by

$$L\left( \boldsymbol{E} | \phi\right)= \prod_{g} \prod_{t=2004}^{2016} \frac{1}{\sqrt{2\pi\sigma^{2}}}\exp\left( - \frac{\left[ \ln\left( E_{g,t}\gamma\right)-\ln\left( D_{g,t}\left( \phi\right) \right) \right]^{2}}{2\sigma^{2}} \right)$$

and the variance $(\sigma^{2})$ is set at 0.01.

### The likelihood for tuberculosis deaths in the electronic tuberculosis treatment register

We rely on the ETR to calibrate the model to the proportion of TB deaths while on treatment. We let $R_{g,t}$ represent the proportion of TB deaths recorded in the ETR, for individuals of sex g in year $t$ (obtained from the outcomes data, deaths on treatment, Table 12). We define exp(γ) as the ratio of the *true* odds of death to the odds of a death recorded on ETR of deaths in TB patients get recorded on the treatment register (allowing for the possibility that the recorded number of deaths is less than the true number of deaths). The model produces estimates of the proportion of deaths in TB patients on treatment of sex in year $t$, $D_{g,t}\left( \phi\right)$, where $\phi$ is the set of input parameters. Then $\gamma$ is estimated as:

$$\gamma=\frac{1}{13\times2}\sum_{t=2004}^{2016} \sum_{g=0}^{1} \mathrm{logit} \left( D_{g,t} \right)-\mathrm{logit} \left( R_{g,t} \right)$$

We set a lower limit of zero (i.e., assuming deaths are not over-reported).

Then, we also apply a logit transformation to the proportions ($D_{g,t}$ and $R_{g,t}$) and specify the likelihood as follows:

$$L\left( \boldsymbol{R} | \phi\right)= \prod_{g} \prod_{t} \frac{1}{\sqrt{2\pi\sigma^{2}}}\exp\left( \frac{\left[ \mathrm{logit} \left( R_{g,t} \right)-\mathrm{logit} \left( D_{g,t}\left( \phi\right) \right)-\gamma\right]^{2}}{2\sigma^{2}} \right)$$

$\sigma^{2}$ is assumed to be at 0.01.

### The likelihood for HIV prevalence in the electronic tuberculosis register

We let $M_{t}\left( \phi\right)$ be the estimated prevalence of HIV in people who are on TB treatment in the model, where $\boldsymbol{\phi}$ represents the set of model input parameters values; and $H_{t}$ represent the prevalence of HIV in people recorded on the treatment register (based on the ETR data, Table 23). We restricted the calibration to HIV prevalence between 2009-2016 because in earlier years, HIV status information was incompletes and there was a high proportion of unknown/unspecified HIV status. We have thus decided to include data from 2009 in which HIV testing coverage was at least 50% (128).

We then define the likelihood as follows

$$L\left( \boldsymbol{H} | \phi\right)=\prod_{t=2009}^{2016} \frac{1}{\sqrt{2\pi\sigma^{2}}}\exp\left( \frac{\left[ \mathrm{logit} \left( M_{t}(\phi) \right)-logit \left( H_{t} \right) \right]^{2}}{2\sigma^{2}} \right)$$

The variance is set to be 0.01, equivalent to a 95% confidence interval of 55-64% if prevalence is 60%.

### The likelihood for the numbers of microbiological tuberculosis tests performed

We also calibrated our model to the recorded number of microbiological tests performed by the South African National Health Laboratory Service (NHLS). We relied on the Nanoo *et al* study for these data which were available for the years 2004 to 2011(Table 24) (52). Although we requested more recent data from the NHLS, we were not able access the data. These data are based on the samples submitted to the NHLS for TB testing and have unique patient identifiers. In the absence of the unique patient identifiers, a probabilistic record-linking process is used to match multiple specimen records to individual patients, which may be subject to bias (52). We apply a factor of 0.9 to these recorded number of laboratory tests to account for a 10% over-estimation in NHLS due to under-linking (informed by personal commutation with Harry Moultrie, National Institute for Communicable Diseases).

We assume that the number of microbiological tests performed follows a Log-Normal distribution and specify a Log-Normal likelihood function for the years 2004 to 2011. We let $D_{t}$ represent the recorded number of microbiological tests in year $t$ as reported by Nanoo *et al.* (Table 24). Then let $M_{t}(\phi)$ represents the model estimates for the number of tests performed in year $t$ where $\phi$ represents the set of input parameters.

Table 24: Recorded numbers of microbiological tuberculosis test performed by year

|  | 2004 | 2005 | 2006 | 2007 | 2008 | 2009 | 2010 | 2011 |
| --- | --- | --- | --- | --- | --- | --- | --- | --- |
| Total microbiological tests (adjusted) | 1259747 | 1571753 | 1844658 | 2214799 | 2555539 | 2713728 | 3061440 | 3175857 |

Data source: Nanoo et al (52).

We then specify the likelihood as follows:

$$L\left( \boldsymbol{D} | \phi\right)=\prod_{t=2004}^{2011} \frac{1}{\sqrt{2\pi\sigma^{2}}}\exp\left( - \frac{\left[ \ln\left( D_{t}\gamma\right)-\ln\left( M_{t}\left( \phi\right)) \right) \right]^{2}}{2\sigma^{2}} \right)$$

The variance is set to be 0.01 and $\gamma=0.9$.

### The likelihood for the prevalence of bacteriologically confirmed active tuberculosis

In the 2018 national TB prevalence survey, the prevalence of bacteriologically confirmed pulmonary TB in individuals who were 15 years and older was 1 094 (95% CI 835 – 1 352) per 100 000 and 675 (95% CI 494 – 855) per 100 000 for males and females, respectively (46).

We let $P_{g}$ be the prevalence of bacteriologically confirmed TB for individuals of sex $g$, as reported for the 1^st^ South African National Prevalence Survey (2018) (46). Then let $M_{g}\left( \phi\right)$ represent the model estimates of the prevalence of pulmonary TB, of sex $g$; where ϕ is the set of input parameter values.

Based on the South African studies by Pepper *et al* and Gupta *et al*, the estimated proportion of TB cases that are exclusively extra-pulmonary tuberculosis (EPTB) in HIV-negative individuals was 13.5% and 8.2%, respectively (22,71). The South African TB prevalence survey report assumed that the proportion of TB cases that were exclusively extra-pulmonary TB was 9.7% (46). In our model we assumed the proportion of TB cases that are exclusively EPTB ($p_{s=0})$ for those with HIV-negative status ($s=0$) to be 10%. Among HIV-positive individuals, the proportion of TB cases that are exclusively EPTB is slightly higher, estimated at 26.3% and 17.6% by Pepper *et al* and Gupta *et al*, respectively (22,71). We therefore assumed 22%, the average from these two studies (22,71) to represent the proportion of TB cases that are exclusively EPTB in HIV-positive ($s=1$) individuals ($p_{s=1}$).

Then, for $H_{g}$ the total number of active TB cases in HIV-positive individuals; $N_{g}$ the total number of active TB cases in HIV-negative individuals; and $T_{g}$ the total number of people in the population, for sex $g$, we estimated the prevalence of pulmonary TB as

$M_{g}=\left( N_{g}\left( 1.0-p_{0} \right)+ H_{g}\left( 1.0-p_{1} \right) \right)/T_{g}$.

We applied a logit transformation to these prevalence proportions from the prevalence survey ${(P}_{g}$) and model $(M_{g}\left( \phi\right)$). Then the likelihood is represented by:

$$L\left( \boldsymbol{P} | \phi\right)= \prod_{g} \frac{1}{\sqrt{2\pi\sigma_{g}^{2}}}\exp\left( - \frac{\left[ \mathrm{logit} \left( P_{g} \right)-\mathrm{logit} \left( M_{g}\left( \phi\right)) \right) \right]^{2}}{2\sigma_{g}^{2}} \right)$$

and the variance is calculated as:

$$\sigma_{g}^{2}= \left( \frac{0.1s_{g}}{P_{g}\left( 1-P_{g} \right)} \right)^{2}$$

where $P_{g}$ is the point prevalence and $s_{g}$ is the survey standard error estimated from the confidence intervals. We multiplied the survey standard error by a factor of 0.1 to ensure the model produced a better fit to the TB prevalence survey data (as the TB prevalence survey would otherwise get very little weight relative to other data sources and our initial attempts to fit the model did not give good fits to the survey prevalence without the 0.1 adjustment).

### Generating posterior distributions

The posterior distributions were simulated numerically by implementing the Incremental Mixture Importance Sampling (IMIS) algorithm (129) in the following steps:

1. $N_{0}=10 000$ input parameters were randomly drawn from the prior distributions in Table 20. Here $\phi_{i}$, for ${i=1, 2, 3,\ldots, N}_{0}$, is the set of different parameter combinations.
2. For each parameter set $\phi_{i}$, a likelihood $L_{i}$ was calculated, by multiplying together the likelihood expressions in sections 11.1-11.7.
3. Weights $w_{i}^{0}$ were calculated as a ratio of the likelihood $L_{i}$ over the sum of all likelihoods.

$$w_{i}^{0}=\frac{L_{i}}{\sum_{j=0}^{N_{0}} L_{j}}$$

4. Importance sampling was then performed to concentrate sampling in regions of parameter spaces that yield the highest likelihood values. The following steps were repeated (*k*-times) until a stopping criterion was met.
   - The weights were sorted, and the maximum weight was found and set as the centre of the new sampling distribution $\phi_{i}^{(k)}$.
   - Mahalanobis distances between the centre $\phi_{i}^{(k)}$ and other prior points $\phi_{i}$ were calculated. These were sorted, and the smallest distances were recorded. Finally, the Mahalanobis distances were calculated with respect to the covariance of prior distributions.
   - B prior points $\phi_{i}$ with the smallest distance to $\phi_{i}^{(k)}$ were selected as a set. Then a weighted covariance of these points in B was calculated.
   - New inputs were sampled from a Gaussian distribution. (These points are those with the smallest distance from the max weight $\phi_{i}^{(k)}$).
   - Then a new likelihood was calculated using the new inputs. The new inputs were then combined with previous inputs from prior distributions and used to calculate new weights

$$w_{i}^{k}=cL_{i}\times\frac{p(\phi_{i})}{q^{\left( k \right)}(\phi)}$$

Where c is chosen so that the weights add to 1 and $q^{\left( k \right)}$ is the mixture sampling distribution:

$$q^{\left( k \right)}= \frac{N_{0}}{N_{k}}p+\frac{B}{N_{k}}\sum_{s=1}^{(k)} H_{s}$$

And $N_{k}$ is the total number of inputs up to the k-th iteration.

1. From the posterior sample, J = 1 000 parameter combinations were resampled. The posterior means for the model estimates were calculated as the average of all outputs generated over 1000 samples. 95% intervals were calculated by taking the 0.25^th^ and the 0.975^th^ percentiles of the outputs.

The stopping criterion was set to be reached when the expected fraction of unique parameter combinations in the posterior sample is at least $0.4$. The expected number of unique parameter combinations is calculated as

$$\frac{1}{N_{k}}\sum_{i=1}^{N_{k}} \left( 1-\left( 1-w_{i} \right)^{J} \right)$$

$N_{k}$ is the total number of inputs up to the k-th iteration, and J is the number of resamples.

## Results from model calibration

### Comparison of prior and posterior distributions

As indicated in Table 20, most of the TB natural history parameters were estimated in a separate analysis and fixed in this current analysis. Table 25 below shows the prior and posterior distributions for parameters varied in this current analysis. Most of the prior and posterior distributions means are similar and the 95% confidence intervals overlap. However, there were some differences with other parameters, in particular the parameters representing empirical treatment and relative rate of screening in TB patients seeking treatment for TB symptoms, compared to those seeking treatment for other conditions (in earlier years). This reflects the uncertainty associated with the parameter due to limited empirical evidence to inform the prior distributions.

Table 25: Comparison of prior and posterior distributions for model parameters

| **Parameter description** | **Prior mean (95% confidence interval)** | **Posterior mean (95% confidence interval)** |
| --- | --- | --- |
| TB transmission probability per contact per day (if infectious individual is smear-positive) | 0.0025 (0.00013 – 0.0184) | 0.0034 (0.0031 - 0.0037) |
| Relative rate of TB incidence for those on ART (controlling for CD4) | 0.81 (0.703 - 0.898) | 0.840 (0.822 - 0.862) |
| Relative rate of TB mortality if on ART | 0.55 (0.392 - 0.703) | 0.498 (0.469 - 0.536) |
| The annual rate of health-seeking in males with smear-negative TB | 2.14 (1.29 - 3.202) | 1.07 (0.903 - 1.212) |
| The annual rate of health-seeking in males in the general population | 1.15 (0.389 - 2.317) | 1.0 (0.76 - 1.3) |
| The annual rate of health-seeking in males due to TB-like symptoms | 0.22 (0.030 - 0.596) | 0.196 (0.163 - 0.224) |
| The proportion of active TB cases seeking treatment who are treated empirically before any microbiological test is done | 0.125 (0.00625 - 0.244) | 0.068 (0.046 - 0.092) |
| The proportion of smear-negative TB cases which are treated empirically if they initially screened negative smear test | 0.33 (0.0168 - 0.653) | 0.28 (0.222 - 0.353) |
| Relative rate of empirical treatment if not seeking treatment because of TB symptoms | 0.5 (0.0 - 1.0) | 0.031 (0.017 - 0.051) |
| Relative rate empirical treatment if symptoms are not due to TB | 0.5 (0.0 - 1.0) | 0.0014 (0.0005 - 0.0029) |
| Relative rate of health-seeking in women, compared to men | 1.55 (1.235 - 1.901) | 1.376 (1.2884 - 1.475) |
| Relative rate of health-seeking in HIV-positive compared to HIV-negative individuals | 3 (1.372 - 5.254) | 4.27 (3.72 - 5.12) |
| Relative rate of screening in TB patients seeking treatment for TB symptoms, compared to those seeking treatment for other conditions |  |  |
| Initial (up to 2005) | 8.71 (4.520 - 14.248) | 11.10 (9.73 - 12.47) |
| Ultimate (after 2012) | 4 (2.005 - 6.671) | 3.84 (3.27 - 4.52) |

ART = antiretroviral therapy; TB=tuberculosis. The ratio of model the estimated (‘true’) tuberculosis cases to the number of recorded deaths classified as TB (described in section 11.1)) was estimated at 1.26 (95% CI 1.22 – 1.29).

### Calibration graphs

The figures below show the comparison of model estimates and data sources data described in Figure 6 to Figure 16. In all figures, model estimates are represented by the solid black lines and the dashed lines represent the 95% confidence intervals for model estimates. The data points are represented by the black dots.

- Figure 7 and Figure 8 show the model fit to adjusted recorded mortality data. Overall, the model resulted in a good fit to the data; however, the model slightly over-estimated the number of TB deaths in 2016 onwards. This may be due to reporting delays in the death data (late recording) because when 2016 data were released, not all the 2016 deaths had been processed. The model slightly under-estimates male TB deaths over the 2008-2012 period. This is consistent with the Thembisa model slightly under-estimating all-cause mortality in men over the 2008-2012 period (130).
- Figure 9 shows the expected tuberculosis deaths in people living with HIV and model estimated tuberculosis deaths in HIV-positive individuals. The model was fairly consisted with the expected TB deaths in HIV-positive individuals but slightly overestimated the deaths from 2007 onwards.
- Figure 10 and Figure 11 show the model fit to adjusted recorded ETR treatment initiations for male and females respectively. The model resulted in an earlier peak in treatment initiations than the ETR data suggest; this could be because the earlier ETR data were less complete. The model was also limited and did not perfectly capture the sex differences in treatment initiations.
- Figure 12 and Figure 13 show the proportions of TB deaths recorded in the ETR for males and females. The model did not match the observed data well. Although we adjusted for incomplete recording of deaths, we did so on the assumption that completeness levels remained constant over time, which might be unrealistic. In ART programmes it has been noted that the recording of mortality in patient record systems has become significantly less complete over time (81), which might explain why we do not match the significant reduction in the recorded deaths in the more recent years.
- Figure 14 shows the model fit to the prevalence of HIV in individuals on treatment. The model did not to fit the ETR HIV prevalence well (underestimating the observed data) before 2014. This is possibly because over the period 2014-2016, 95% of people in the ETR were tested (had known HIV status), while testing was less complete in the earlier years (e.g., in 2009, only 53% people were tested). Thus, it is possible that the earlier (less complete) data overstated the true TB prevalence if HIV testing was biased towards people suspected of being at HIV risk, or if there was a bias due to people on ART being known HIV-positive cases.
- Figure 15 shows the model fit to the number of microbiological TB tests performed. The model captured in the increasing trend for numbers of TB tests but slightly underestimated the reported data in recent years.
- Figure 16 and Figure 17 show the model fit to the prevalence of TB for males and females. Overall, the model resulted in a good fit for the observed prevalence data.

Figure 7: Recorded number of tuberculosis deaths (adjusted) and model estimated deaths in males 15+

Figure 8: Recorded number of tuberculosis deaths (adjusted) and model estimated deaths in females 15+ years

Figure 9: Expected tuberculosis deaths in people living with HIV and model estimated tuberculosis deaths in HIV-positive individuals

Figure 10: Recorded number of tuberculosis cases initiated on treatment (adjusted) and model estimated tuberculosis cases on treatment in males 15+ years

Figure 11: Recorded number of tuberculosis cases initiated on treatment (adjusted) and model estimated tuberculosis cases on treatment in females 15+ years

Figure 12: Proportion of tuberculosis deaths recorded in the electronic tuberculosis treatment register and model estimates for proportion of tuberculosis deaths on treatment for males 15+ years

Figure 13: Proportion of tuberculosis deaths recorded in the electronic tuberculosis treatment register and model estimates for proportion of tuberculosis deaths for females 15+ years on treatment

Figure 14: HIV prevalence in the electronic tuberculosis register and model estimated HIV prevalence in adults on treatment

Figure 15: The numbers of microbiological tuberculosis tests performed and model estimates for microbiological tests performed

Figure 16: The prevalence of bacteriologically confirmed active tuberculosis and model estimated prevalence of tuberculosis in males 15+ years

Figure 17: The prevalence of bacteriologically confirmed active tuberculosis and model estimated prevalence of tuberculosis in females 15+ years

## Comparison with other model estimates

The World Health Organization (WHO) (131) and the Institute for Health Metrics and Evaluation (IHME) (132) are two main agencies that produce global, regional, and national TB burden estimates, and South Africa relies on their reports for TB burden estimates. In this section, the TB burden estimates produced by our model (Thembisa TB/HIV) will be compared to the 2019 South African TB burden estimates produced by the WHO and IHME to assess the overall consistency between the models.

*Overall tuberculosis burden*

Overall, our model had major differences with the number of new TB cases estimated by the IMHE and WHO: WHO and IHME estimates were 32% and 55% higher respectively than Thembisa estimates (Table 26), but the models were closer in their estimates of total TB deaths. We note that the WHO and IMHE estimates include all ages whereas we only consider the adult (15+ years) population in our model. The proportion of TB of all forms in children (<15 years) is estimated at approximately 11% in South Africa (28). As such, we would expect our estimates to be relatively lower than both the WHO and IMHE estimates (Table 26).

Table 26: Comparison of 2019 tuberculosis disease burden estimates by the three models: Thembisa, IHME and WHO

|  | **Thembisa TB/HIV** | **IMHE**⁋ | **WHO*** |
| --- | --- | --- | --- |
| **New tuberculosis cases** |  |  |  |
| **HIV-positive** | 156 000 | 203 700 | 209 000 |
| **HIV-negative** | 117 000 | 218 800 | 151 000 |
| **% new TB in HIV-positive** | 57% | 48% | 58% |
| **Totals** | 273 000 | 422 500 | 360 000 |
| **Tuberculosis deaths** |  |  |  |
| **HIV-positive** | 34 000 | 40 100 | 36 000 |
| **HIV-negative** | 18 000 | 19 820 | 22 000 |
| **% TB deaths in HIV-positive** | 65% | 67% | 62% |
| **Totals** | 52 000 | 59 920 | 58 000 |

Source: Author’s own estimates (Thembisa). Ledesma *et al* 2021 (132); 2020 WHO TB report (131). Note: both WHO and IHME report for all ages, whereas for Thembisa, we report for adults only (15+ years). ⁋IMHE figures obtained from summing HIV-sex-disaggregated data. *WHO sex-specific data obtained from reading a graph so may not be the exact.

Another apparent difference between our estimates and the IHMEs was that the IMHE estimated a relatively low proportion of TB incidence in HIV-positive individuals (48%). In contrast, the WHO and Thembisa TB/HIV estimated a higher proportion of TB cases in HIV-positive individuals, with proportions of 58% and 57%, respectively. For mortality, all models estimated a similarly high proportion of TB deaths in HIV-positive individuals (62-67%).

*Sex differences*

For 2019, the WHO and Thembisa TB/HIV model estimated a high proportion of TB cases in males with 59% and 60%, respectively, whereas the IMHE estimated a lower proportion of TB cases in males, 42% (Table 27). There was however some consistency between our model and the IHME’s estimates for mortality, with both models estimating a higher proportion of TB deaths in males than in females. The WHO did not report mortality by sex.

Table 27: Comparison of 2019 tuberculosis disease burden estimates by the three models, by sex: Thembisa, IHME and WHO

|  | **Thembisa TB/HIV** | **IMHE**⁋ | **WHO*** |
| --- | --- | --- | --- |
| **New tuberculosis cases** |  |  |  |
| **Males** | 164 000 | 179 500 | 214 000 |
| **Females** | 109 000 | 243 000 | 146 000 |
| **% of TB in males** | 60% | 42% | 59% |
| **Totals** | 273 000 | 422 500 | 360 000 |
| **Tuberculosis mortality** |  |  |  |
| **Males** | 31 000 | 32 800 |  |
| **Females** | 21 500 | 27 120 |  |
| **% of TB deaths in males** | 60% | 54% |  |
| **Totals** | 52 000 | 59 920 |  |

Sources: Ledesma *et al* 2021 (132); 2020 WHO TB reported (131). ⁋IMHE figures obtained from summing HIV-sex-disaggregated data. *WHO sex-specific data obtained from reading a graph so may not be the exact.

The notable differences described above may be driven by differences in the methodological approaches for estimating the tuberculosis burden and the data sources used for inputs (Table 28). The IMHE relies largely on mortality data and indirectly estimates incidence from case fatality ratios estimated from a regression model (132). The WHO on the other hand relied on the 2018 prevalence survey and assumptions about TB disease duration to estimate TB incidence (131). The WHO’s estimates of disease duration are based on literature reviews and a simple dynamic model of three compartments (susceptible, untreated TB, and treated TB) (133). Prior to 2019 (i.e., before the release of the South African TB prevalence survey), to estimate incidence for South Africa, the WHO mainly relied on TB notification data combined with expert opinion about case detection gaps (133,134).

We have used a dynamic transmission model which considers the tuberculosis natural history (i.e., disease progression, recovery), transmission dynamics, health-seeking behaviours and diagnostic algorithms, and intervention impacts, whereas the IHME estimates are not based on dynamic modelling. To simulate the effects of HIV on tuberculosis incidence and mortality in our model, we used available evidence to estimate relative risks (which varied by CD4 count and ART status) of TB incidence and mortality for HIV positive individuals. Additionally, we performed a formal Bayesian calibration process to ensure the estimates are consistent with epidemiological data, including the recorded mortality data, notified cases initiating treatment and the latest 2018 TB prevalence survey.

To estimate mortality in HIV-negative individuals the WHO uses the vital register data; however, the data were adjusted for TB/HIV miscoding (the WHO obtains these adjusted data from the IMHE) (133,134). For the HIV-positive population, the WHO applies HIV-specific case-fatality ratios to the estimated TB incidence (131). The IMHE uses a mixed-effects regression model to estimate the proportion of HIV-TB cases among all TB cases, then they estimate relative risks of TB deaths in HIV-positive individuals (132). Based on this, they estimate the proportions of TB deaths attributable to HIV (132).

To further understand what influences the differences between these models, a systematic analysis involving a comparison of the methods and data sources would be required. Garcıa-Basteiro *et al*, performed an analysis which compared the 2015 TB mortality estimates produced by the WHO and IHME, and explored the factors that drove the observed differences (135). Overall, it seems the differences in estimation approaches and data sources used led to these differences. The authors suggested that differences in the use of prevalence survey data and case detection rates may explain most of the observed differences in the mortality estimates produced by the WHO and IHME (135).

Table 28: General differences between Thembisa TB/HIV, the Institute for Health Metrics and Evaluation and the World Health Organization for 2019 TB burden estimates

|  | Thembisa TB/HIV | IMHE | WHO |
| --- | --- | --- | --- |
| Modelling and approach | Dynamic transmission model  Includes: TB natural history natural history (i.e., disease progression, recovery), transmission dynamics, health-seeking behaviours, and diagnostic algorithms, and impacts of interventions. | Static model.  Meta-regression approach including covariates such as smoking prevalence, diabetes, indoor air pollution, alcohol, and health system access. | Simple dynamic model to estimate TB disease duration accounting the effect of HIV and ART.  Use case fatality ratios estimated in the literature. |
| Data sources | 2018 national TB prevalence survey Recorded deaths from the vital registry cleaned and adjusted (1997-2016), electronic tuberculosis register (2004-2019). Literature review on model parameters. | Recorded deaths from the vital registry and verbal autopsies | Recorded deaths – vital register, national TB prevalence survey. Case fatality ratios estimated in the literature |
| Strata | HIV, CD4 stage, ART,  Sex, age | HIV, sex, age | HIV, sex, age |
| Modelling TB incidence | Depends on fast progression, reactivation, relapse. Incorporated the effect of HIV, ART and CD4 count. | Used meta-regression to estimate mortality-to-incidence ratios. Then use mortality-to-incidence ratios and cause-specific mortality estimates to compute incidence.  (Did not use the 2018 South African TB prevalence - based on communication with Hmwe Kyu) | TB prevalence surveys combined with estimates of the duration of disease. |
| Modelling TB mortality | Assumed death rates for TB treated and untreated cases. Incorporated the effect of HIV, ART and CD4 count. | Use vital registry and verbal autopsy data. Use mixed-effects regression models to estimate the proportion of HIV-TB cases among all TB cases. Estimated relative risks of TB deaths in HIV-positive individuals, then use a population attributable fraction approach to estimated deaths attributable to HIV. | For HIV-negative individuals: used vital registry data cleaned and analyzed by the IHME.  For HIV-positive individuals, apply case fatality ratios to TB incidence, accounting for antiretroviral treatment's protective effect. |

ART: antiretroviral therapy; IHME: Institute for Health Metrics and Evaluation; TB=Tuberculosis; WHO: World Health Organization

## Additional figures: impact of interventions on tuberculosis mortality

Figure 18: The impact of programmatic interventions on tuberculosis deaths.

a) DOTS, b) IPT, c) ART, d) scaled-up screening, and e) Xpert MTB/RIF. Solid lines represent the estimated mean reductions in tuberculosis deaths. f) The impact of combined interventions on tuberculosis deaths. The black lines represent the estimated mean tuberculosis deaths in the baseline scenario with all existing interventions included, and the grey represents the counterfactual scenario with no intervention included. All dashed lines represent the 95% confidence intervals.

## Additional figures: effect of Xpert MTB/RIF on diagnostic pathway outcomes

Table 29: The effect of Xpert MTB/RIF on laboratory diagnoses and treatment initiations

|  | **False positive laboratory diagnoses in adults** | **Adults without TB starting TB treatment** | **Adults starting TB treatment without a positive lab diagnosis** |
| --- | --- | --- | --- |
| **Baseline** |  |  |  |
| **2011** | 41886 | 38317 | 89683 |
| **2012** | 35361 | 32932 | 74313 |
| **2013** | 27724 | 26436 | 63470 |
| **2014** | 25376 | 24445 | 57424 |
| **2015** | 22826 | 22240 | 52607 |
| **2016** | 22418 | 21897 | 50578 |
| **2017** | 22984 | 22440 | 48811 |
| **2018** | 23390 | 22836 | 47130 |
| **2019** | 23071 | 22568 | 46005 |
| **Counterfactual, No Xpert MTB/RIF** | |  |  |
| **2011** | 50163 | 45113 | 97887 |
| **2012** | 52179 | 46899 | 88527 |
| **2013** | 54062 | 48577 | 82904 |
| **2014** | 56024 | 50323 | 78265 |
| **2015** | 57000 | 51206 | 73975 |
| **2016** | 55979 | 50341 | 70929 |
| **2017** | 57393 | 51603 | 68782 |
| **2018** | 58408 | 52516 | 66597 |
| **2019** | 57614 | 51844 | 64578 |

## Sensitivity analysis for latent TB prevalence assumptions

- There is substantial uncertainty around the initial LTBI prevalence in 1985, and using the CORTIS data to set the age-specific initial assumptions in 1985 might not be ideal. There are two important sources of bias in the CORTIS data. The first is that the data relate to 2016-2018 (8) whereas we are trying to set assumptions for the population in 1985. Although we use age-specific data to avoid problems with the sampled age distribution not matching the true age distribution in 1985, our model simulations suggest that the prevalence of LTBI has been declining over time (see Figure 19 pane d below), in line with international trends (136). This would suggest that the age-specific CORTIS data may understate the true LTBI prevalence estimates in 1985.
- The second source of bias is that the CORTIS data are collected from five sites in low-income communities with high TB prevalence, and these are unlikely to give us a nationally-representative picture of LTBI prevalence. This would suggest the CORTIS data may overstate the true LTBI prevalence at a national level. It is likely that these two sources of bias offset one another to some extent, and in the absence of better data, the CORTIS data are probably a reasonable starting point when setting the initial LTBI prevalence in the model.
- To assess the sensitivity of the model results to this parameter, we re-calibrated the model allowing for the uncertainty in the effect of age on the initial LTBI prevalence. (This parameter accounts for most of the variation in initial LTBI prevalence.) We assigned a gamma prior with a mean of 0.064 (the same as the value used in the original model calibration) and a standard deviation of 0.016. After refitting the model, the posterior mean for this parameter was 0.090 (95% CI: 0.082-0.099), i.e. substantially higher than the prior mean, and implying a much higher initial LTBI prevalence than previously estimated. Figure 19 (panels a-d) below compares the model outputs in the original calibration (‘Fixed LTBI’) with the model outputs when we vary the LTBI age parameter in the calibration (‘Varied LTBI’). Although the higher initial LTBI prevalence estimate does not appear to have much impact on the modelled trends in TB prevalence and mortality (panels a and c), the modelled annual number of new TB cases in adults are substantially lower when the initial LTBI prevalence is higher (panel b), and as expected, overall prevalence of LTBI prevalence is also higher (panel d).

Figure 19: Adult TB trends under different assumptions about the effect of age on initial LTBI prevalence

In panel (d), the ‘crude’ CORTIS data are the prevalence estimates for South African adults (15+) after re-weighting the age-specific prevalence estimates using the 2017 South African population age distribution. The ‘adjusted’ CORTIS data are based on the same age standardization, but in addition we adjust for the sensitivity and specificity of the IGRA assay used in the CORTIS trial (assumed to be 0.78 and 0.96 respectively (9)).

- There are a number of reasons to be suspicious of the results obtained in this sensitivity analysis, when allowing the age effect of the initial LTBI to vary in the calibration process. Firstly, the annual TB incidence in adults is implausibly low; in most of the posterior simulations, the modelled number of treated TB cases is *less* than the number of cases recorded in the ETR. (As discussed in section 11.3 of the supplementary material, we would expect the ETR to understate the true number of treated TB cases.) Secondly, the estimated LTBI prevalence estimates seem extremely high when compared against regional averages (136). We therefore do not consider these as an acceptable ‘alternative’ set of model outputs, but present them here for the sake of understanding the sensitivity of the model outputs to the initial LTBI assumptions.

## Additional figures: TB incidence and mortality by different model experiments

Figure 20: TB incidence and mortality by different counterfactual scenarios

- With the removal of the DOTS, Xpert MTB/RIF and IPT interventions, the TB incidence and mortality trends remain roughly the same as in the baseline scenario, so these trends in these scenarios are not shown here.
- Without increased screening and ART, the TB incidence and mortality would have peaked much later, and would have declined much more slowly than in the baseline scenario.

## Additional figures: Sensitivity analysis – correlation coefficients for model input parameters and model outcomes

We assessed the correlation between each parameter varied in the calibration process, and the model estimated new TB cases and deaths in 2019 (Table 30); we also assessed the correlation between the input parameters (Table 31). The model outputs (new TB cases and deaths) were generated for each of the 1000 parameter combinations in the posterior sample. We then generated scatter plots to show the relationship between the model parameters and new TB cases and deaths for 2019 (Figures 21 and 22, respectively).

- - As expected, the TB transmission probability was positively associated with incidence (r=0.82) and mortality (r=0.45).
  - The negative association (r=-0.43) between the relative rate of TB mortality on ART and TB mortality is unexpected. However, we also see that the parameter is negatively associated with other parameters that likely influence mortality (e.g., health-seeking rates in HIV-positive individuals (r=-0.34) and women (r=-0.19)). We also observed that the change in the relative rates of symptom screening between 2005 and 2012 were positively correlated with the relative rate of TB mortality on ART (r=-0.42 in 2005 and 0.30 in 2012). This may be because there is a trade-off in how much of the observed TB mortality reduction up to 2019 can be explained by ART versus increased TB screening. That is, if a lot of the reduction is due to ART (low relative rate of TB mortality on ART), then a relatively small amount of the change is attributable to changes in screening (i.e., low values of the change in the relative rate of symptom screening between 2005 and 2012).
  - If empirical treatment were beneficial, we would expect a negative correlation between the empirical treatment parameters and TB incidence and mortality. However, we observed a positive association between 1) the proportions of empirical treatment in active TB cases who are treated empirically before any microbiological test is done (r=0.76 for incidence; r=0.35 for mortality), and 2) those treated empirically if they initially screened negative smear test (r=0.38 for incidence; r=0.22 for mortality). We fitted our model to the total number of people treated in the South African electronic treatment register (ETR) to estimate these parameters. The positive associations with the empirical treatment parameters suggest that more people were likely misdiagnosed (i.e., empirical treatment is not as impactful in reducing incidence and mortality due to treating false-positive individuals).
  - If a particular health-seeking parameter (rate) is beneficial, we expect a negative relationship between TB incidence and mortality outcomes. We observed a positive association between the outcomes and the relative rate of health-seeking in HIV-positive individuals (r=0.55 for incidence; r=0.42 for mortality). This is possibly because as this parameter increases, the fraction of treated TB cases who are HIV-positive cases increases. As HIV-positive individuals are less infectious than HIV-negative individuals, increasing the proportion of treated active TB individuals who are HIV-positive increases the level of TB transmission. We also observed that the health-seeking relative rate in HIV-positive versus HIV-negative parameter is positively correlated with the transmission probability, r=0.41.

Table 30: Correlation coefficients for model input parameters varied in the calibration process, and specified outcomes

| **Model parameters** | **TB incidence, 2019** | **TB mortality, 2019** |
| --- | --- | --- |
| TB transmission probability per contact per day (if infectious individual is smear-positive) | 0.82 | 0.45 |
| Relative rate of TB incidence for those on ART (controlling for CD4) | 0.48 | 0.30 |
| Relative rate of TB mortality if on ART | -0.28 | -0.43 |
| The annual rate of health-seeking in males with smear-negative TB | -0.13 | -0.15 |
| The annual rate of health-seeking in males in the general population | -0.07 | -0.21 |
| The annual rate of health-seeking in males due to TB-like symptoms | 0.11 | 0.10 |
| The proportion of active TB cases seeking treatment who are treated empirically before any microbiological test is done | 0.76 | 0.35 |
| The proportion of smear-negative TB cases which are treated empirically if they initially screened negative smear test | 0.38 | 0.22 |
| Relative rate of empirical treatment if not seeking treatment because of TB symptoms | -0.21 | -0.27 |
| Relative rate empirical treatment if symptoms are not due to TB | -0.17 | -0.16 |
| Relative rate of health-seeking in women, compared to men | 0.12 | 0.05 |
| Relative rate of health-seeking in HIV-positive compared to HIV-negative individuals | 0.55 | 0.42 |
| Relative rate of screening in TB patients seeking treatment for TB symptoms, compared to those seeking treatment for other conditions* |  |  |
| Initial (up to 2005) | -0.20 | 0.09 |
| Ultimate (from 2012) | -0.29 | -0.26 |

ART=antiretroviral therapy. TB=tuberculosis. *The initial rate applies up to 2005, the ultimate rate applies from 2012, with linear interpolation over the intervening years (2006-2011).

## Additional figures: Sensitivity analysis – pairwise correlations between model parameters

Table 31: Pair-wise correlation coefficients between model parameters

|  | Transmission probability | RR TB on ART | RR TB mort on ART | Health seeking active | Health seeking general population | Health seeking TB-like symptoms | Empirical treatment, no screen | Empirical treatment, negative screen | RR empiric, no symptoms | RR empiric, no TB | RR health seeking in women | RR health seeking in HIV+ vs HIV- | RR screening symptomatic initial | RR screening symptomatic ultimate |
| --- | --- | --- | --- | --- | --- | --- | --- | --- | --- | --- | --- | --- | --- | --- |
| Transmission probability | 1.00 |  |  |  |  |  |  |  |  |  |  |  |  |  |
| RR TB on ART | 0.52 | 1.00 |  |  |  |  |  |  |  |  |  |  |  |  |
| RR TB mort on ART | -0.43 | -0.24 | 1.00 |  |  |  |  |  |  |  |  |  |  |  |
| Health seeking active | 0.08 | -0.34 | -0.19 | 1.00 |  |  |  |  |  |  |  |  |  |  |
| Health seeking general population | 0.01 | -0.01 | 0.38 | -0.32 | 1.00 |  |  |  |  |  |  |  |  |  |
| Health seeking TB-like symptoms | 0.10 | -0.08 | -0.06 | 0.76 | -0.44 | 1.00 |  |  |  |  |  |  |  |  |
| Empirical treatment, no screen | 0.88 | 0.53 | -0.16 | -0.32 | 0.21 | -0.20 | 1.00 |  |  |  |  |  |  |  |
| Empirical treatment, negative screen | 0.25 | 0.29 | 0.18 | -0.41 | 0.28 | -0.15 | 0.31 | 1.00 |  |  |  |  |  |  |
| RR empiric, no symptoms | -0.12 | 0.06 | 0.19 | 0.35 | -0.41 | 0.24 | -0.21 | -0.38 | 1.00 |  |  |  |  |  |
| RR empiric, no TB | -0.23 | -0.02 | 0.31 | -0.49 | 0.21 | -0.40 | 0.04 | 0.13 | -0.13 | 1.00 |  |  |  |  |
| RR health seeking in women | 0.21 | 0.26 | -0.19 | -0.03 | 0.02 | 0.17 | 0.15 | 0.27 | -0.15 | 0.16 | 1.00 |  |  |  |
| RR health seeking in HIV+ vs HIV- | 0.41 | 0.50 | -0.34 | -0.20 | -0.39 | 0.10 | 0.37 | 0.30 | -0.15 | -0.12 | 0.21 | 1.00 |  |  |
| RR screening symptomatic initial | -0.18 | -0.33 | -0.42 | 0.32 | -0.32 | 0.29 | -0.35 | -0.21 | -0.01 | -0.11 | 0.06 | -0.12 | 1.00 |  |
| RR screening symptomatic ultimate | -0.30 | 0.04 | 0.30 | -0.18 | 0.44 | -0.26 | -0.15 | -0.20 | -0.11 | 0.37 | -0.43 | -0.22 | -0.16 | 1.00 |

RR=relative rate. TB=tuberculosis.

## Additional figures: Sensitivity analysis – scatter plots of estimated number of new TB cases and deaths and model parameters

Figure 21: Scatter plots to show the relationship between estimated new TB cases and model parameters

Figure 22: Scatter plots to show the relationship between estimated TB mortality and model parameters

## References

1. Vynnycky E, Fine PE. The natural history of tuberculosis: the implications of age-dependent risks of disease and the role of reinfection. Epidemiol Infect. 1997;119(2):183–201.

2. Dye C, Floyd K. Chapter 16. Tuberculosis. In: In Disease Control Priorities in Developing Countries. 2006. p. 289–309.

3. Kasaie P, Andrews JR, Kelton WD, Dowdy DW. Timing of tuberculosis transmission and the impact of household contact tracing: An agent-based model. Am J Respir Crit Care Med. 2014;189(7):845–52.

4. Johnson L, Dorrington R. Thembisa version 4.1: A model for evaluating the impact of HIV/AIDS in South Africa. 2018.

5. Johnson L, Dorrington R. Thembisa version 4.4: A model for evaluating the impact of HIV/AIDS in South Africa. 2021.

6. Johnson L. THEMBISA version 1.0: A model for evaluating the impact of HIV / AIDS in South Africa. 2014.

7. Williams BG, Korenromp EL, Gouws E, Schmid GP, Auvert B, Dye C. HIV Infection, Antiretroviral Therapy, and CD4 + Cell Count Distributions in African Populations. J Infect Dis. 2006;194(10):1450–8.

8. Scriba TJ, Fiore-Gartland A, Penn-Nicholson A, Mulenga H, Kimbung Mbandi S, Borate B, et al. Biomarker-guided tuberculosis preventive therapy (CORTIS): a randomised controlled trial. Lancet Infect Dis. 2021;21(3):354–65.

9. Pai M, Zwerling A, Menzies D. Systematic review: T-cell-based assays for the diagnosis of latent tuberculosis infection: an update. Ann Intern Med. 2008;149(3):177–84.

10. Wood R, Liang H, Wu H, Middelkoop K, Oni T. Changing prevalence of TB infection with increasing age in high TB burden townships in South Africa. Int J Tuberc Lung Dis. 2010;14(4):406–12.

11. Mahomed H, Hawkridge T, Verver S, Geiter L, Hatherill M, Abrahams D, et al. Predictive factors for latent tuberculosis infection among adolescents in a high-burden area in South Africa. Int J Tuberc Lung Dis. 2011;15(3):331–6.

12. Hanifa Y, Grant AD, Lewis J, Corbett EL, Fielding K, Churchyard G. Prevalence of latent tuberculosis infection among gold miners in South Africa. Int J Tuberc Lung Dis. 2009;13(1):39–46.

13. Ncayiyana JR, Bassett J, West N, Westreich D, Musenge E, Emch M, et al. Prevalence of latent tuberculosis infection and predictive factors in an urban informal settlement in Johannesburg, South Africa: a cross-sectional study. BMC Infect Dis. 2016;16(1):661.

14. Samson M, Porter N, Orekoya O, Hebert JR, Adams SA, Bennett CL, et al. Incidence of occupational latent tuberculosis infection in South African healthcare workers. Eur Respir J. 2015;45(5):1364–73.

15. Global Tuberculosis Report 2014. World Health Organization. Geneva, Switzerland; 2014.

16. Wood R, Lawn SD, Caldwell J, Kaplan R, Middelkoop K, Bekker LG. Burden of new and recurrent tuberculosis in a major South African city stratified by age and HIV-status. PLoS One. 2011;6(10).

17. Nhamoyebonde S, Leslie A. Biological differences between the sexes and susceptibility to tuberculosis. J Infect Dis. 2014;209(SUPPL. 3).

18. WHO. Global tuberculosis report. Geneva: World Health Organization; 2019.

19. Horton KC, Macpherson P, Houben RMGJ, White G, Corbett EL. Sex Differences in Tuberculosis Burden and Notifications in Low- and Middle-Income Countries : A Systematic Review and Meta-analysis. PLoS Med. 2016;21:1–23.

20. Austin JF, Dick JM, Zwarenstein M. Gender disparity amongst TB suspects and new TB patients according to data recorded at the South African Institute of Medical Research laboratory for the Western Cape Region of South Africa. Int J Tuberc Lung Dis. 2004;8(4):435–9.

21. Department of Health. South African Demographic and Health Survey 1998. Department of Health. Pretoria, South Africa; 1998.

22. Gupta RK, Lawn SD, Bekker L-G, Caldwell J, Kaplan R, Wood R. Impact of HIV and CD4 count on tuberculosis diagnosis: analysis of citywide data from Cape Town, South Africa. Int J Tuberc Lung Dis. 2013;17(8):1014–22.

23. Icky, Looker C, Dodd PJ, Plumb ID, Shanaube K, Muyoyeta M, et al. Comparison of indoor contact time data in Zambia and Western Cape, South Africa suggests targeting of interventions to reduce Mycobacterium tuberculosis transmission should be informed by local data. BMC Infect Dis. 2016;16(1):71.

24. Singla R, Singla N, Sarin R, Arora VK. Influence of pre-treatment bacillary load on treatment outcome of pulmonary tuberculosis patients receiving DOTS under revised national tuberculosis control programme. Indian J Chest Dis Allied Sci. 2005;47(1).

25. Acuña-Villaorduña C, Ayakaka I, Schmidt-Castellani LG, Mumbowa F, Marques-Rodrigues P, Gaeddert M, et al. Host determinants of infectiousness in smear-positive patients with pulmonary tuberculosis. Open Forum Infect Dis. 2019;6(6):1–9.

26. Behr MA, Warren SA, Salamon H, Hopewell PC, Ponce De Leon A, Daley CL, et al. Transmission of Mycobacterium tuberculosis from patients smear-negative for acid-fast bacilli. Lancet. 1999;353(9151):444–9.

27. Dodd PJ, Looker C, Plumb ID, Bond V, Schaap A, Shanaube K, et al. Age- and Sex-Specific Social Contact Patterns and Incidence of Mycobacterium tuberculosis Infection. Am J Epidemiol. 2016;183(2):156–66.

28. Dodd PJ, Gardiner E, Coghlan R, Seddon JA. Burden of childhood tuberculosis in 22 high-burden countries: A mathematical modelling study. Lancet Glob Heal. 2014;2(8).

29. Johnstone-Robertson SP, Mark D, Morrow C, Middelkoop K, Chiswell M, Aquino LDH, et al. Social mixing patterns within a South African township community: Implications for respiratory disease transmission and control. Am J Epidemiol. 2011;174(11):1246–55.

30. de WarouxO le P, Cohuet S, Ndazima D, Kucharski A., Juan-Giner AJ, Flasche S, et al. Characteristics of human encounters and social mixing patterns relevant to infectious diseases spread by close contact: A survey in Southwest Uganda. BMC Infect Dis. 2018;18(1):172.

31. Mossong J, Hens N, Jit M, Beutels P, Auranen K, Mikolajczyk R, et al. Social contacts and mixing patterns relevant to the spread of infectious diseases. PLoS Med. 2008;5(3):0381–91.

32. Bacaër N, Ouifki R, Pretorius C, Wood R, Williams B. Modeling the joint epidemics of TB and HIV in a South African township. J Math Biol. 2008;57(4):557–93.

33. Houben RMGJ, Lalli M, Sumner T, Hamilton M, Pedrazzoli D, Bonsu F, et al. TIME Impact - a new user-friendly tuberculosis (TB) model to inform TB policy decisions. BMC Med. 2016;14(1):56.

34. Menzies NA, Cohen T, Lin HH, Murray M, Salomon JA. Population Health Impact and Cost-Effectiveness of Tuberculosis Diagnosis with Xpert MTB/RIF: A Dynamic Simulation and Economic Evaluation. PLoS Med. 2012;9(11).

35. Ellis PK, Martin WJ, Dodd PJ. CD4 count and tuberculosis risk in HIV-positive adults not on ART: a systematic review and meta-analysis. PeerJ. 2017;5:e4165.

36. Van Rie A, Westreich D, Sanne I. Tuberculosis in patients receiving antiretroviral treatment: Incidence, risk factors, and prevention strategies. J Acquir Immune Defic Syndr. 2011;56(4):349–55.

37. Fenner L, Atkinson A, Boulle A, Fox MP, Prozesky H, Zürcher K, et al. HIV viral load as an independent risk factor for tuberculosis in South Africa: collaborative analysis of cohort studies. J Int AIDS Soc. 2017;20(1):21327.

38. Andrews JR, Noubary F, Walensky RP, Cerda R, Losina E, Horsburgh CR. Risk of progression to active tuberculosis following reinfection with Mycobacterium tuberculosis. Clin Infect Dis. 2012;54(6):784–91.

39. Tiemersma EW, van der Werf MJ, Borgdorff MW, Williams BG, Nagelkerke NJD. Natural history of tuberculosis: Duration and fatality of untreated pulmonary tuberculosis in HIV negative patients: A systematic review. PLoS One. 2011;6(4).

40. National Tuberculosis Management Guidelines 2014. 2014.

41. National Tuberculosis Management Guidelines. Department of Health. Pretoroia, South Africa; 2008.

42. Onozaki I, Law I, Sismanidis C, Zignol M, Glaziou P, Floyd K. National tuberculosis prevalence surveys in Asia, 1990-2012: An overview of results and lessons learned. Trop Med Int Heal. 2015;

43. Claassens MM, Van Schalkwyk C, Floyd S, Ayles H, Beyers N. Symptom screening rules to identify active pulmonary tuberculosis: Findings from the Zambian South African Tuberculosis and HIV/AIDS Reduction (ZAMSTAR) trial prevalence surveys. PLoS One. 2017;12(3):1–12.

44. Den Boon S, White NW, Van Lill SWP, Borgdorff MW, Verver S, Lombard CJ, et al. An evaluation of symptom and chest radiographic screening in tuberculosis prevalence surveys. Int J Tuberc Lung Dis. 2006;10(8):876–82.

45. Corbett EL, Charalambous S, Moloi VM, Fielding K, Grant AD, Dye C, et al. Human immunodeficiency virus and the prevalence of undiagnosed tuberculosis in African gold miners. Am J Respir Crit Care Med. 2004;170(6):673–9.

46. Department of Health. The First National Tuberculosis Prevalence Survey: South Africa 2018. Pretoria, South Africa; 2021.

47. Kweza PF, Van Schalkwyk C, Abraham N, Uys M, Claassens MM, Medina-Marino A. Estimating the magnitude of pulmonary tuberculosis patients missed by primary health care clinics in South Africa. Int J Tuberc Lung Dis. 2018;22(3):264–72.

48. Claassens MM, Jacobs E, Cyster E, Jennings K, James A, Dunbar R, et al. Tuberculosis cases missed in primary health care facilities: Should we redefine case finding? Int J Tuberc Lung Dis. 2013;17(5):608–14.

49. Chihota VN, Ginindza S, McCarthy K, Grant AD, Churchyard G, Fielding K. Missed opportunities for TB investigation in primary care clinics in South Africa: Experience from the XTEND trial. PLoS One. 2015;10(9):1–11.

50. South Africa Demographic and Health Survey 2003. Department of Health Pretoria, South Africa; 2007.

51. Use of health facilities and levels of selected health conditions in South Africa: Findings from the General Household Survey, 2011. Statistics South Africa. Pretoria, South Africa;

52. Nanoo A, Izu A, Ismail NA, Ihekweazu C, Abubakar I, Mametja D, et al. Nationwide and regional incidence of microbiologically confirmed pulmonary tuberculosis in South Africa, 2004-12: A time series analysis. Lancet Infect Dis. 2015;15(9):1066–76.

53. National Tuberculosis Management Guidelines. Department of Health. Pretoroia, South Africa; 2009.

54. Hippner P, Sumner T, Houben RMGJ, Cardenas V, Vassall A, Bozzani F, et al. Application of provincial data in mathematical modelling to inform sub-national tuberculosis program decision-making in South Africa. PLoS One. 2019;14(1):1–11.

55. Sumner T, Bozzani F, Mudzengi D, Hippner P, Houben RM, Cardenas V, et al. Estimating the Impact of Tuberculosis Case Detection in Constrained Health Systems: An Example of Case-Finding in South Africa. Am J Epidemiol. 2019;188(6):1155–64.

56. Department of Health. National Tuberculosis Management Guidelines 2008. Department of Health, Republic of South Africa 2014; 2008.

57. Vassall A, Siapka M, Foster N, Cunnama L, Ramma L, Fielding K, et al. Cost-effectiveness of Xpert MTB/RIF for tuberculosis diagnosis in South Africa: a real-world cost analysis and economic evaluation. Lancet Glob Heal. 2017;5(7):e710–9.

58. McCarthy KM, Grant AD, Chihota V, Ginindza S, Mvusi L, Churchyard GJ, et al. What happens after a negative test for tuberculosis? Evaluating adherence to TB diagnostic algorithms in South African primary health clinics. J Acquir Immune Defic Syndr. 2016;71(5):e119–26.

59. Naidoo P, Dunbar R, Lombard C, Du Toit E, Caldwell J, Detjen A, et al. Comparing tuberculosis diagnostic yield in smear/culture and xpert1 MTB/RIF-based algorithms using a non-randomised stepped-wedge design. PLoS One. 2016;11(3):1–13.

60. Boehme CC, Nabeta P, Hillemann D, Nicol MP, Shenai S, Krapp F, et al. Rapid Molecular Detection of Tuberculosis and Rifampin Resistance. N Engl J Med. 2010;225–37.

61. Keflie TSS, Ameni G. Microscopic examination and smear negative pulmonary tuberculosis in ethiopia. Pan Afr Med J. 2014;19:1–10.

62. Cattamanchi A, Dowdy DW, Davis JL, Worodria W, Yoo S, Joloba M, et al. Sensitivity of direct versus concentrated sputum smear microscopy in HIV-infected patients suspected of having pulmonary tuberculosis. BMC Infect Dis. 2009;9:1–8.

63. Horne D, Kohli M, Zifodya J, I S, N D, D T, et al. Xpert MTB/RIF and Xpert MTB/RIF Ultra for pulmonary tuberculosis and rifampicin resistance in adults. Cochrane Database Syst Rev. 2019;(6).

64. Parker RA. Implications of tuberculosis sputum culture test sensitivity on accuracy of other diagnostic modalities. Am J Respir Crit Care Med. 2019;199(5):664.

65. Steingart KR, Ng V, Henry M, Hopewell PC, Ramsay A, Cunningham J, et al. Sputum processing methods to improve the sensitivity of smear microscopy for tuberculosis: a systematic review. Lancet Infect Dis. 2006;6(10):664–74.

66. Naidoo P, Theron G, Rangaka MX, Chihota VN, Vaughan L, Brey ZO, et al. The South African Tuberculosis Care Cascade: Estimated Losses and Methodological Challenges. J Infect Dis. 2017;216(Suppl 7):S702–13.

67. MacPherson P, Houben RM, Glynn JR, Corbett EL, Kranzer K. Pre-treatment loss to follow-up in tuberculosis patients in low- and lower-middle-income countries and high-burden countries: a systematic review and meta-analysis. Bull World Health Organ. 2014;92(2):126–38.

68. Botha E, Den Boon S, Lawrence KA, Reuter H, Verver S, Lombard CJ, et al. From suspect to patient: Tuberculosis diagnosis and treatment initiation in health facilities in South Africa. Int J Tuberc Lung Dis. 2008;12(8):936–41.

69. Improving the diagnosis and treatment of smear-negative pulmonary and extra-pulmonary tuber- culosis among adults and adolescents: Recommendations for HIV-prevalent and resource-constrained settings. Geneva, Switzerland; 2007.

70. Purohit M, Mustafa T. Laboratory diagnosis of extra-pulmonary tuberculosis (EPTB) in resource-constrained setting: State of the art, challenges and the need. J Clin Diagnostic Res. 2015;9(4):EE01–6.

71. Pepper DJ, Schomaker M, Wilkinson RJ, Azevedo V, Maartens G. Independent predictors of tuberculosis mortality in a high HIV prevalence setting: A retrospective cohort study. AIDS Res Ther. 2015;12(1):1–9.

72. Pronyk RM, Makhubele MB, Hargreaves JR, Tollman SM, Hausler HP. Assessing health seeking behaviour among tuberculosis patients in rural South Africa. Int J Tuberc Lung Dis. 2001;

73. Gupta A, Wood R, Kaplan R, Bekker L, Lawn SD. Tuberculosis Incidence Rates during 8 Years of Follow-Up of an Antiretroviral Treatment Cohort in South Africa : Comparison with Rates in the Community. PLoS One. 2012;7(3):1–10.

74. Hanrahan CF, Selibas K, Deery CB, Dansey H, Clouse K, Bassett J, et al. Time to Treatment and Patient Outcomes among TB Suspects Screened by a Single Point-of-Care Xpert MTB/RIF at a Primary Care Clinic in Johannesburg, South Africa. PLoS One. 2013;8(6).

75. Hermans S, Caldwell J, Kaplan R, Cobelens F, Wood R. The impact of the roll-out of rapid molecular diagnostic testing for tuberculosis on empirical treatment in Cape Town, South Africa. Bull World Health Organ. 2017;95(8):554–63.

76. Van’t Hoog AH, Cobelens F, Vassall A, Van Kampen S, Dorman SE, Alland D, et al. Optimal triage test characteristics to improve the cost-effectiveness of the Xpert MTB/RIF assay for TB diagnosis: A decision analysis. PLoS One. 2013;8(12).

77. Rees K, Muditambi N, Maswanganyi M, Railton J, McIntyre JA, Struthers HE, et al. The impact of implementing a Xpert MTB/RIF algorithm on drug-sensitive pulmonary tuberculosis: A retrospective analysis. Epidemiol Infect. 2018;146(2):246–55.

78. Marx FM, Dunbar R, Enarson DA, Beyers N. The Rate of Sputum Smear-Positive Tuberculosis after Treatment Default in a High-Burden Setting: A Retrospective Cohort Study. PLoS One. 2012;7(9):1–9.

79. Edginton ME, Wong ML, Phofa R, Mahlaba D, Hodkinson HJ. Tuberculosis at Chris Hani Baragwanath Hospital: Numbers of patients diagnosed and outcomes of referrals to district clinics. Int J Tuberc Lung Dis. 2005;9(4):398–402.

80. Anderegg N, Johnson LF, Zaniewski E, Althoff KN, Balestre E, Law M, et al. All-cause mortality in HIV-positive adults starting combination antiretroviral therapy: Correcting for loss to follow-up. Aids. 2017;31:S31–40.

81. Johnson LF, Dorrington RE, Laubscher R, Hoffmann CJ, Wood R, Fox MP, et al. A comparison of death recording by health centres and civil registration in South Africans receiving antiretroviral treatment. J Int AIDS Soc. 2015;18(1):1–7.

82. Berry KM, Rodriguez CA, Berhanu RH, Ismail N, Mvusi L, Long L, et al. Treatment outcomes among children, adolescents, and adults on treatment for tuberculosis in two metropolitan municipalities in Gauteng Province, South Africa. BMC Public Health. 2019;19(1):1–17.

83. Vree M, Huong NT, Duong BD, Co N V., Sy DN, Cobelens FG, et al. High mortality during tuberculosis treatment does not indicate long diagnostic delays in Vietnam: A cohort study. BMC Public Health. 2007;7:1–7.

84. Kayigamba FR, Bakker MI, Mugisha V, de Naeyer L, Gasana M, Cobelens F, et al. Adherence to Tuberculosis Treatment, Sputum Smear Conversion and Mortality: A Retrospective Cohort Study in 48 Rwandan Clinics. PLoS One. 2013;8(9):1–10.

85. Osawa T, Watanabe M, Morimoto K, Okumura M, Yoshiyama T, Ogata H, et al. Serum procalcitonin levels predict mortality risk in patients with pulmonary tuberculosis: A single-center prospective observational study. J Infect Dis. 2020;222(10):1651–4.

86. Muttath R, Andrews M, Prabhu D. Treatment outcome in new smear positive pulmonary tuberculosis patients with and without immunosuppression on RNTCP regimen: a comparative observational study. Int J Res Med Sci. 2017;5(2):384.

87. Kolappan C, Subramani R, Kumaraswami V, Santha T, Narayanan PR. Excess mortality and risk factors for mortality among a cohort of TB patients from rural south India. Int J Tuberc Lung Dis. 2008;12(1):81–6.

88. Lienhardt C, Manneh K, Bouchier V, Lahai G, Milligan PJM, McAdam KPWJ. Factors determining the outcome of treatment of adult smear-positive tuberculosis cases in The Gambia. Vol. 2, International Journal of Tuberculosis and Lung Disease. 1998. p. 712–8.

89. Saunders MJ, Wingfield T, Datta S, Montoya R, Ramos E, Baldwin MR, et al. A household-level score to predict the risk of tuberculosis among contacts of patients with tuberculosis: a derivation and external validation prospective cohort study. Lancet Infect Dis. 2020;20(1):110–22.

90. Kaplan R, Hermans S, Caldwell J, Jennings K, Bekker LG, Wood R. HIV and TB co-infection in the ART era: CD4 count distributions and TB case fatality in Cape Town. BMC Infect Dis. 2018;18(1):1–9.

91. Kaplan R, Caldwell J, Middelkoop K, Bekker L-G, Wood R. Impact of ART on TB case fatality stratified by CD4 count for HIV-positive TB patients in Cape Town. South Africa (2009-2011). J Acquir Immune Defic Syndr. 2014;66(5):487–94.

92. Van den Broek J, Mfinanga S, Moshiro C, O’Brien R, Mugomela A, Lefi M. Impact of human immunodeficiency virus infection on the outcome of treatment and survival of tuberculosis patients in Mwanza, Tanzania. Int J Tuberc Lung Dis. 1998;2(7):547–52.

93. Heunis JC, Kigozi NG, Chikobvu P, Botha S, Van Rensburg HD. Risk factors for mortality in TB patients: A 10-year electronic record review in a South African province. BMC Public Health. 2017;17(1):1–7.

94. Osman M, van Schalkwyk C, Naidoo P, Seddon JA, Dunbar R, Dlamini SS, et al. Mortality during tuberculosis treatment in South Africa using an 8-year analysis of the national tuberculosis treatment register. Sci Rep. 2021;11(1):1–10.

95. Mugusi FM, Mehta S, Villamor E, Urassa W, Saathoff E, Bosch RJ, et al. Factors associated with mortality in HIV-infected and uninfected patients with pulmonary tuberculosis. BMC Public Health. 2009;9:1–8.

96. Lambert ML, Hasker E, Van Deun A, Roberfroid D, Boelaert M, Van der Stuyft P. Recurrence in tuberculosis: Relapse or reinfection? Lancet Infect Dis. 2003;3(5):282–7.

97. Korenromp EL, Scano F, Williams BG, Dye C, Nunn P. Effects of Human Immunodeficiency Virus Infection on Recurrence of Tuberculosis after Rifampin-Based Treatment: An Analytical Review. Clin Infect Dis. 2003;37(1):101–12.

98. Marx FM, Dunbar R, Enarson DA, Williams BG, Warren RM, Van Der Spuy GD, et al. The temporal dynamics of relapse and reinfection tuberculosis after successful treatment: A retrospective cohort study. Clin Infect Dis. 2014;58(12):1676–83.

99. Naidoo K, Dookie N. Insights into Recurrent Tuberculosis: Relapse Versus Reinfection and Related Risk Factors, Tuberculosis. IntechOpen. 2018;

100. Sonnenberg P, Murray J, Glynn JR, Shearer S, Kambashi B, Godfrey-Faussett P. HIV-1 and recurrence, relapse, and reinfection of tuberculosis after cure: A cohort study in South African mineworkers. Lancet. 2001;358(9294):1687–93.

101. den Boon S, Bateman ED, Borgdorff MW, de Villiers C, Enarson D a, Irusen E, et al. High prevalence of tuberculosis in previously treated patients, Cape Town, South Africa. Emerg Infect Dis. 2007;13(8):1189–94.

102. Marx FM, Floyd S, Ayles H, Godfrey-Faussett P, Beyers N, Cohen T. High burden of prevalent tuberculosis among previously treated people in Southern Africa suggests potential for targeted control interventions. Eur Respir J. 2016;(48):1227–30.

103. National Department of Health. Guidelines for Tuberculosis Preventive Therapy among HIV Infected Individuals in South Africa. 2010.

104. Department of Health. The South African Antiretroviral Treatment Guidelines 2013. 2013;

105. Van Ginderdeuren E, Bassett J, Hanrahan C, Mutunga L, Van Rie A. Health system barriers to implementation of TB preventive strategies in South African primary care facilities. PLoS One. 2019;14(2):1–12.

106. Wood R, Bekker LG. Isoniazid preventive therapy for tuberculosis in South Africa: An assessment of the local evidence base. South African Med J. 2014;104(3):174–7.

107. Maharaj B, Gengiah TN, Yende-Zuma N, Gengiah S, Naidoo A, Naidoo K. Implementing isoniazid preventive therapy in a tuberculosis treatment-experienced cohort on ART. Int J Tuberc Lung Dis. 2017;21(5):537–43.

108. Takarinda KC, Choto RC, Harries AD, Mutasa-Apollo T, Chakanyuka-Musanhu. Routine implementation of isoniazid preventive therapy in HIV-infected patients in seven pilot sites in Zimbabwe. Public Heal Action. 2017;7(1):55–60.

109. Nyathi S, Dlodlo RA, Satyanarayana S, Takarinda KC, Tweya H, Hove S, et al. Isoniazid preventive therapy: Uptake, incidence of tuberculosis and survival among people living with HIV in Bulawayo, Zimbabwe. PLoS One. 2019;14(10):1–12.

110. Okoli EI, Roets L. Health system challenges: An obstacle to the success of isoniazid preventive therapy. South African Med J. 2016;106(11):1079–81.

111. Dhungana GP, Thekkur P, Chinnakali P, Bhatta U, Pandey B, Zhang WH. Initiation and completion rates of isoniazid preventive therapy among people living with HIV in Far-Western Region of Nepal: A retrospective cohort study. BMJ Open. 2019;9(5):1–9.

112. Ayele HT, Van Mourik MSM, Debray TPA, Bonten MJM. Isoniazid prophylactic therapy for the prevention of tuberculosis in HIV infected adults: A systematic review and meta-analysis of randomized trials. PLoS One. 2015;10(11):1–16.

113. Churchyard GJ, Fielding KL, Lewis JJ, Coetzee L, Corbett EL, Godfrey-Faussett P, et al. A trial of mass isoniazid preventive therapy for tuberculosis control. N Engl J Med. 2014;370(4):301–10.

114. Shisana O, Labadarios D, Rehle T, Simbayi L, Zuma K, Dhansay A, Reddy P, Parker W, Hoosain E, Naidoo P, Hogoro C, Mchiza Z, Steyn NP, Dwane N, Makoae M, Maluleke T, Ramlagan S, Zungu N, Evans MG, Jacobs L FM. The South African National health and Nutrition Examination Survey SANHANES-1. Cape Town: HSRC Press; 2013.

115. South Africa Demographic and Health Survey 2016. Pretoria, South Africa; 2019.

116. Al-Rifai RH, Pearson F, Critchley JA, Abu-Raddad LJ. Association between diabetes mellitus and active tuberculosis: A systematic review and meta-analysis. PLoS One. 2017;12(11):1–26.

117. Lönnroth K, Williams BG, Cegielski P, Dye C. A consistent log-linear relationship between tuberculosis incidence and body mass index. Int J Epidemiol. 2010;39(1):149–55.

118. Leung CC, Lam TH, Chan WM, Yew WW, Ho KS, Leung G, et al. Lower risk of tuberculosis in obesity. Arch Intern Med. 2007;167(12):1297–304.

119. Kolappan C, Gopi P. Tobacco smoking and pulmonary tuberculosis. Thorax. 2002;(57):964–6.

120. Lönnroth K, Williams BG, Stadlin S, Jaramillo E, Dye C. Alcohol use as a risk factor for tuberculosis – a systematic review. BMC Public Health. 2008;8(8):289.

121. Rehm J, Samokhvalov A V, Neuman MG, Room R, Parry C, Lönnroth K, et al. The association between alcohol use, alcohol use disorders and tuberculosis (TB). A systematic review. BMC Public Health. 2009;9:450.

122. Gupta RK, Lucas SB, Fielding KL, Lawn SD. Prevalence of tuberculosis in post-mortem studies of HIV-infected adults and children in resource-limited settings: A systematic review and meta-analysis. Aids. 2015;29(15):1987–2002.

123. Johnson LF, May MT, Dorrington RE, Cornell M, Boulle A, Egger M, et al. Estimating the impact of antiretroviral treatment on adult mortality trends in South Africa : A mathematical modelling study. PLoS Med. 2017;1–17.

124. Podewils LJ, Bantubani N, Bristow C, Bronner LE, Peters A, Pym A, et al. Completeness and Reliability of the Republic of South Africa National Tuberculosis (TB) Surveillance System. BMC Public Health. 2015;15(1):1–11.

125. Myburgh H, Peters RPH, Hurter T, Grobbelaar CJ, Hoddinott G. Transition to an in-facility electronic tuberculosis register: Lessons from a South African pilot project. South Afr J HIV Med. 2020;21(1):1–7.

126. Mlotshwa M, Smit S, Williams S, Reddy C, Medina-Marino A. Evaluating the electronic tuberculosis register surveillance system in Eden District, Western Cape, South Africa, 2015. Glob Health Action [Internet]. 2017;10(1). Available from: https://doi.org/10.1080/16549716.2017.1360560

127. Dreyer AW, Mbambo D, Machaba M, Oliphant CEM, Claassens MM. Tuberculosis cure rates and the ETR.Net: Investigating the quality of reporting treatment outcomes from primary healthcare facilities in Mpumalanga province, South Africa. BMC Health Serv Res. 2017;17(1):1–6.

128. Johnsona LF, Rehlea TM, Joosteb S, Bekkerc LG. Rates of HIV testing and diagnosis in South Africa: Successes and challenges. AIDS. 2015;29(11):1401–9.

129. Raftery AE, Bao L. Estimating and Projecting Trends in HIV/AIDS Generalized Epidemics Using Incremental Mixture Importance Sampling. Biometrics. 2010;66(4):1162–1173.

130. Johnson LF, Dorrington RE. Thembisa version 4.3: A model for evaluating the impact of HIV/AIDS in South Africa. 2020.

131. Global Tuberculosis Report 2020. Geneva: World Health Organization; 2020;

132. Ledesma JR, Ma J, Vongpradith A, Maddison ER, Novotney A, Biehl MH, et al. Global, regional, and national sex differences in the global burden of tuberculosis by HIV status, 1990–2019: results from the Global Burden of Disease Study 2019. Lancet Infect Dis. 2021;3099(21):1–20.

133. Glaziou P, Sismanidis C, Pretorius C, Timimi H, Floyd K. Global TB Report 2015 : Technical appendix on methods used to estimate the global burden of disease caused by TB. World Health Organisation. Geneva; 2015.

134. Philippe G, Dodd PJ, Dean A, Floyd K. Methods used by WHO to estimate the Global burden of TB disease. 2019.

135. Garcıa-Basteiro AL, Brew J, Williams B, Borgdorff M, Cobelens F. What is the true tuberculosis mortality burden? Differences in estimates by the World Health Organization and the Global Burden of Disease study. Int J Epidemiol. 2018;47(5):1549–60.

136. Houben RMGJ, Dodd PJ. The Global Burden of Latent Tuberculosis Infection: A Re-estimation Using Mathematical Modelling. PLOS Med. 2016;13(10):e1002152.

1. Suppose A is the number of TB cases who are tested microbiologically, B is the number who are treated empirically (without microbiological testing) and C are the number of symptomatic TB cases that don’t get treated. We define Y as B/(B+C), however, the Pepper and Pronyk studies report B/(A+B). If C > A, as one might expect, given the low rates of screening historically, then the Pronyk and Pepper studies are over-estimating the proportions we are interested in. [↑](#footnote-ref-2)
2. standard error = ((upper limit – lower limit) / 3.92) [↑](#footnote-ref-3)
